# Rh D blood group conversion using transcription activator-like effector nucleases

**DOI:** 10.1038/ncomms8451

**Published:** 2015-06-16

**Authors:** Young-Hoon Kim, Hyun O. Kim, Eun J. Baek, Ryo Kurita, Hyuk-Jin Cha, Yukio Nakamura, Hyongbum Kim

**Affiliations:** 1 Graduate School of Biomedical Science and Engineering/College of Medicine, Hanyang University, Seoul 133-791, South Korea; 2 Department of Pharmacology, Brain Korea 21 Plus Project for Medical Sciences, Graduate Program of Nano Science and Technology, Yonsei University College of Medicine, Seoul 120-752, South Korea; 3 Department of Laboratory Medicine, Yonsei University College of Medicine, Seoul 120-752, South Korea; 4 Department of Laboratory Medicine, Hanyang University College of Medicine, Seoul 133-791, South Korea; 5 Department of Research and Development, Central Blood Institute, Japanese Red Cross Society, Tokyo 135-8521, Japan; 6 Department of Life Sciences, College of Natural Sciences, Sogang University, Seoul 121-742, Korea; 7 Division of Cell Engineering, RIKEN BioResource Center, Tsukuba, Ibaraki 305-0074, Japan

## Abstract

Group O D-negative blood cells are universal donors in transfusion medicine and methods for converting other blood groups into this universal donor group have been researched. However, conversion of D-positive cells into D-negative is yet to be achieved, although conversion of group A or B cells into O cells has been reported. The Rh D blood group is determined by the *RHD* gene, which encodes a 12-transmembrane domain protein. Here we convert Rh D-positive erythroid progenitor cells into D-negative cells using *RHD*-targeting transcription activator-like effector nucleases (TALENs). After transfection of TALEN-encoding plasmids, *RHD*-knockout clones are obtained. Erythroid-lineage cells differentiated from these knockout erythroid progenitor cells do not agglutinate in the presence of anti-D reagents and do not express D antigen, as assessed using flow cytometry. Our programmable nuclease-induced blood group conversion opens new avenues for compatible donor cell generation in transfusion medicine.

## Abstract (web summary)

Group O/RhD− blood can be safely transfused to any recipient and methods for converting other blood groups into this group hold therapeutic potential. By using programmable nucleases, here the authors edit the gene that determines the RhD blood group and convert the RhD+ into RhD− erythroid progenitor cells.

## Graphical abstract

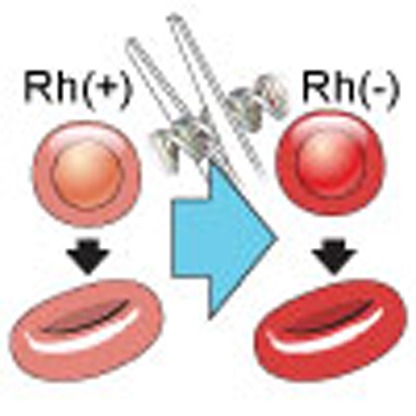

Blood group systems are important for blood transfusion. In clinical transfusion, the ABO and Rh D blood group systems are the most critical among 33 human blood group systems that have been identified (http://www.isbtweb.org/working-parties/red-cell-immunogenetics-and-blood-group-terminology/blood-group-terminology). Because type O Rh D-negative blood cells are universal donors for transfusion, attempts have been made to convert other blood groups into type O by enzymatically removing antigenic carbohydrates^1^,^2^,^3^,^4^. The ABO blood group type is determined by a glycosyltransferase activity that attaches α-N-acetylgalactosamine (for group A) or α-D-galactose (for group B) to the H antigen on the surface of red blood cells. Furthermore, specific exoglycosidases that cleave these bonds between carbohydrate moieties are available and can convert type A or B cells into type O cells^1^,^2^,^3^,^4^. However, because the Rh D group is determined by the expression of the RHD protein on blood cells rather than by the activity of certain enzymes, such enzymatic manipulations are not a desirable approach for the conversion of Rh D-positive into D-negative cells, which is yet to be achieved.

The Rh D blood system was discovered in 1940 (ref. 5) and, since then, a number of other Rh antigens have been identified. D is the most immunogenic and clinically important among 50 well-defined antigens in the Rh system^6^. D antigenicity is determined by the RHD protein, a 12-transmembrane domain protein encoded by *RHD*, a gene on chromosome 1 (refs 7, 8). Roughly 85%, 95% and more than 99.5% of Caucasians, black Africans and east Asians are D-positive, respectively^9^. The main cause of the D-negative phenotype in Caucasians is the homozygous deletion of the whole *RHD* gene^10^. In Africans, *RHD* deletion is also the most common mechanism of D-negativity, but ∼67% of D-negative Africans have at least one copy of RHD*Ψ, an *RHD* allele inactivated by a 37-bp duplication at the intron 3-exon 4 boundary, which may generate a frameshift and premature termination codon (PTC), and a nonsense mutation in exon 6 (ref. 11). Most people are D-positive or -negative, although a minor fraction of the population are D-variants, with phenotypes that include weak D, partial D and DEL^12^.

The D antigen poses a danger for Rh D-negative people. Because those who are Rh D-negative do not have naturally occurring antibodies against the D antigen, adverse effects may not occur when an Rh D-negative person is first exposed to Rh D-positive cells through blood transfusion or by giving birth to an Rh D-positive baby. After such an initial exposure, however, an Rh D-negative person can develop anti-Rh D antibodies, which can induce immune responses against Rh D-positive cells. When the Rh D-negative person is again exposed to Rh D-positive cells, these immune responses can cause adverse effects including haemolysis or abortion of subsequent D-positive babies. The ability to convert Rh D-positive into Rh D-negative cells could provide a starting point for the development of a potential therapeutic modality for these problems.

Programmable nucleases, which include zinc-finger nucleases (ZFNs), transcription activator-like effector nucleases (TALENs) and RNA-guided engineered nucleases (RGENs), enable targeted genetic modifications in cells and organisms^13^. The scope of programmable nuclease-based genome editing covers gene disruptions, insertions, point mutagenesis (or correction) and chromosomal rearrangements such as large deletions, inversions, duplications and translocations. Gene knockout or disruption is the simplest form of programmable nuclease-based genome editing and can be achieved by making a double-stranded break in a specific locus using only one or one pair of programmable nucleases in the absence of donor template. Programmable nuclease-induced double-stranded breaks can be repaired through error-prone nonhomologous-end joining, which often leads to the generation of small insertions or deletions, allowing gene disruption. We have previously used ZFNs, TALENs and RGENs to disrupt protein-coding genes in various human cells^14^,^15^,^16^,^17^,^18^.

We postulated that programmable nuclease-based editing of blood group-determining genes could lead to blood-group conversion. As a proof-of-concept study, here we disrupted *RHD* in Rh D-positive human erythroid progenitor cells using two different pairs of TALENs. *RHD*-knockout erythroid progenitor cells were obtained and gave rise to erythroid-lineage cells that show an Rh D-negative cell phenotype in flow cytometry and agglutination tests. These data provide evidence that blood group conversion can be achieved using programmable nuclease-based gene editing.

## Results

### Design and validation of TALENs targeting *RHD*

**Figure 1:**
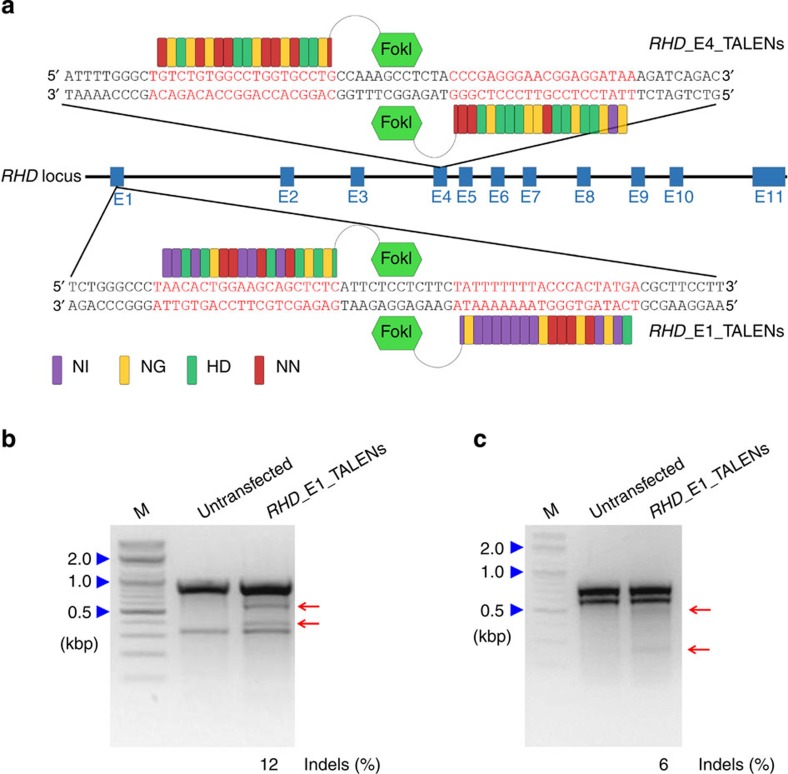
TALENs targeting the human *RHD* gene. (**a**) Schematic of the TALEN-targeting sites in the *RHD* gene. Blue boxes indicate exons. *RHD*_E1_TALENs and *RHD*_E4_TALENs represent the TALEN pairs that target sequences (shown in a red colour) in exon 1 and exon 4, respectively. The red, yellow, green and purple rectangular boxes in the TALENs symbolize the TALE repeat units that recognize guanine, thymine, cytosine and adenine, respectively. (**b**,**c**) T7E1 assay using 293T cells after transfection with plasmids encoding TALENs targeting *RHD* exon 1 (**b**, *RHD*_E1_TALENs) or exon 4 (**c**, *RHD*_E4_TALENs), respectively. The sizes of marker (M) bands are shown on the left (kbp, kilobase pairs). Arrows indicate the expected positions of DNA bands cleaved by T7E1. The numbers at the bottom of the gel indicate mutation percentages measured by band intensities.

To completely disrupt the *RHD* gene, we first obtained a TALEN pair that targets upstream of the protein-coding region; a TALEN pair targeting exon 1 was prepared (*RHD*_E1_TALENs; Fig. 1a). We next determined that this exon 1 target sequence is included in all transcript variants. Transcript information from NCBI and Ensembl showed that the human *RHD* gene has collectively 10 transcript variants including two that do not produce proteins (Supplementary Fig. 1). Exon 4 is included in all eight coding sequences, whereas exon 1 is included in seven coding sequences. Furthermore, exon 4 is the mutation locus of *RHD* in some Rh D-negative people^11^. Thus, we also designed TALENs that target exon 4 (*RHD*_E4_TALENs; Fig. 1a).

To validate the activity of the designed TALENs, we transfected plasmids encoding them into HEK293T cells and performed a T7E1 assay. The mutation frequencies at the target sites in *RHD* exons 1 and 4 were 12% and 6%, respectively (Fig. 1b,c), indicating that both pairs of TALENs have activity at the target sites.

### Generation of clones containing *RHD* mutations

**Figure 2:**
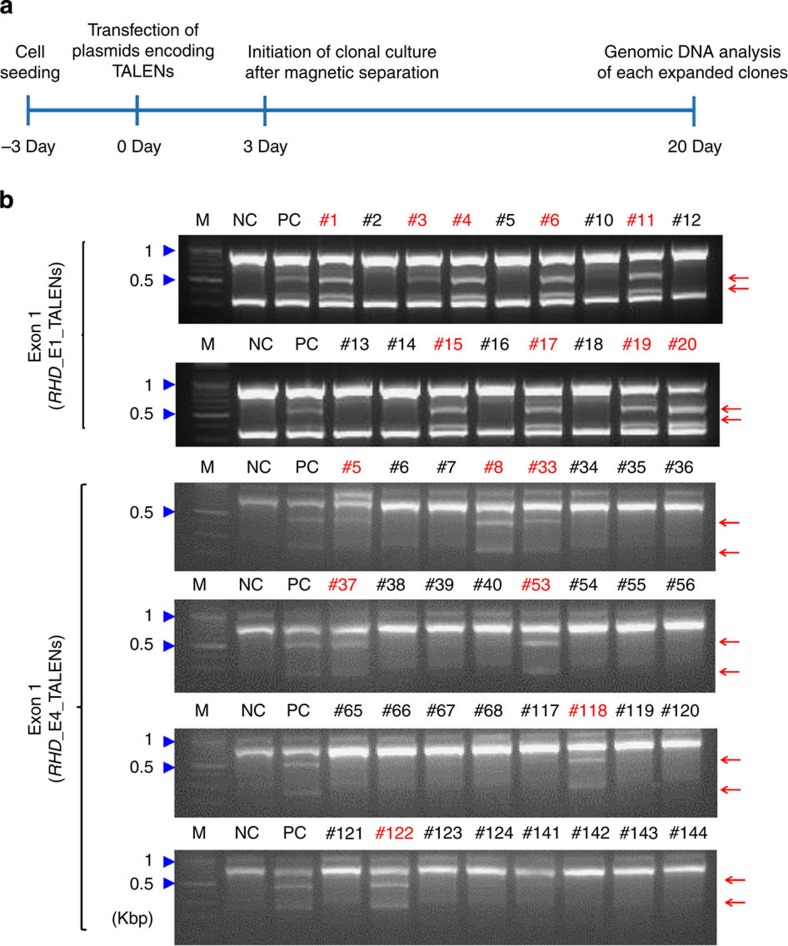
Generation of *RHD*-mutated erythroid progenitor cells. (**a**) Schematic representation illustrating the process of *RHD*-mutated clone generation. Clonal culture of HiDEP-1 erythroid progenitor cells was initiated 3 days after transfection with plasmids encoding TALENs that target *RHD*. Genomic DNA from each clone was analysed 17 days after the initiation of clonal culture. (**b**) T7E1-based clonal analysis. The genomic DNA isolated from each clone was subjected to the T7E1 assay. Arrows indicate the expected position of DNA bands cleaved by T7E1. The sizes of marker (M) bands are shown on the left (kbp, kilobase pairs). Clones containing mutations in the target sites were marked with red clone numbers. Untransfected cells and a cell population transfected with the TALEN plasmids were used as the negative control (NC) and positive control (PC), respectively. M: Markers

We next attempted to use these TALENs to make *RHD*-knockout cells from Rh D-positive erythroid progenitor cells. Our source of erythroid progenitor cells was the HiDEP-1 cell line, which is derived from induced pluripotent stem cells generated from fibroblasts from an Rh D-positive (DD) donor^19^. For efficient generation of knockout cells, we used a magnetic reporter plasmid that expresses H-2K^k^ in the presence of programmable nuclease activity at the target sequence as previously reported^15^,^17^,^20^ (Supplementary Fig. 2). Three days after co-transfection with the reporter plasmid and plasmids encoding *RHD*_E1_TALENs or *RHD*_E4_TALENs, H-2K^k+^ HiDEP-1 cells were magnetically separated^15^,^20^ and seeded into 96-well plates for dilution cloning at an average density of 0.25 cells per well (Fig. 2a). Individual clones were isolated 17 days after the clonal cell seeding; genomic DNA was isolated from each clone and analysed. For efficient screening of mutant clones, genomic DNA from three (*RHD*_E1_TALENs) or four (*RHD*_E4_TALENs) clones was grouped, mixed and subjected to the T7E1 assay. This group T7E1 assay showed that six out of seven clone groups from the *RHD*_E1_TALEN-transfected cells and nine out of thirty-seven clone groups from the *RHD*_E4_TALEN-transfected cells included at least one mutant clone (Supplementary Fig. 3). Each individual clone in these positive clone groups was then subjected to the T7E1 assay. In cells transfected with *RHD*_E1_TALENs, this screening identified 9 mutant clones out of 20 total (Supplementary Fig. 3; Fig. 2b). Subsequent sequencing analysis showed that these nine clones comprise seven monoallelic out-of-frame mutant clones, 1 monoallelic in-frame mutant clone (3 nucleotide deletion), and 1 biallelic mutant clone that contains one in-frame mutation (6 nucleotide deletion) and one out-of-frame mutation (2 nucleotide deletion)(Supplementary Fig. 4a). Because in-frame mutations can lead to incomplete gene knockout, we attempted to obtain biallelic out-of-frame mutant clones. For this purpose, we again co-transfected the reporter plasmid and the plasmids encoding *RHD*_E1_TALENs into one (#17) of the 7 monoallelic out-of-frame mutant clones and performed clonal culture after magnetic separation. Sequencing of DNA from 7 subclones derived from this monoallelic mutant clone showed that all the subclones contain the 2 nucleotide deletion that was initially observed in the parent clone. Among the 7 subclones, one subclone (#17-3), which contained biallelic out-of-frame mutations, was designated as E1_B (#17-3; Supplementary Fig. 4b) and expanded for additional study.

Similarly, in cells co-transfected with the reporter plasmid and the plasmid encoding *RHD*_E4_TALENs, the two-step T7E1 assay revealed that 7 out of 148 clones contain mutations near the target sites in exon 4 (Supplementary Fig. 3; Fig. 2b). Sequencing showed that these seven clones comprise four monoallelic in-frame mutant clones (#33, #37, #53 and #122), one monoallelic out-of-frame mutant clone (#8) and two biallelic out-of-frame mutant clones (#5, #118; Supplementary Fig. 4c). One of the two biallelic mutant clones and the monoallelic out-of-frame mutant clone were designated as E4_B (#5) and E4_M (#8), respectively, and expanded for further experiments.

**Figure 3:**
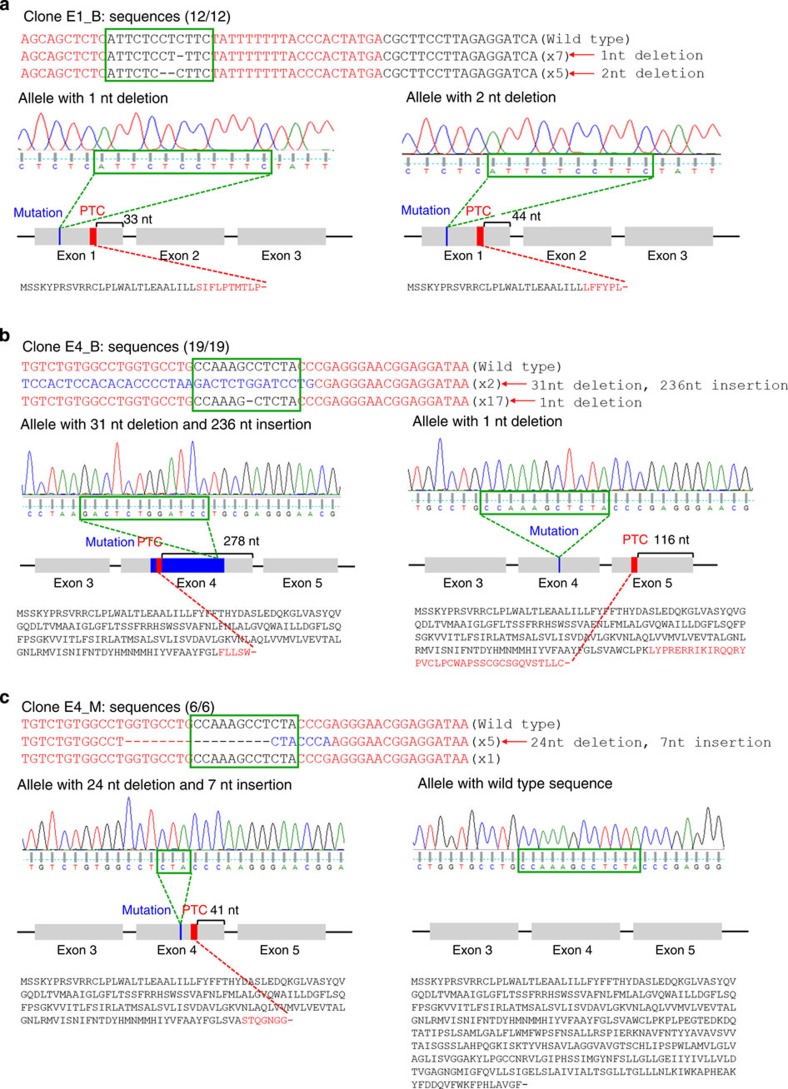
DNA sequences of *RHD*-mutated clones. The *RHD* gene DNA sequences from the parental cells, clones with biallelic mutations in exon 1 (E1_B; **a**) or exon 4 (E4_B; **b**), and a clone with a monoallelic mutation in exon 4 (E4_M; **c**). TALE-binding sites are in a red font and spacer regions are indicated with green boxes. Deleted bases are indicated by dashes and inserted bases are shown in a blue font. The number of occurrences is shown in parentheses (for example, × 7 and × 5 indicate the number of each sequence). The sequence and sequencing chromatogram for each allele are shown. The locus of each mutation, the PTC generated by the mutation and the distance between the PTC and the exon–intron junction are depicted in a schematic of the *RHD* gene. Expected protein sequence translated from each allele are displayed, such that mutated protein sequences generated by a nuclease-induced frameshifting mutation are shown in a red font and translation termination is indicated with a dash. nt, nucleotide.

We next examined the sequencing results to determine whether the selected mutant clones have PTCs in *RHD* exons. Both *RHD* alleles of clone E1_B had PTCs within exon 1 (Fig. 3a). In clone E4_B, PTCs were observed in exon 4 of one allele and in exon 5 of the other (Fig. 3b). In clone E4_M, a PTC was in exon 4 of one mutated allele (Fig. 3c). We have indicated the locations of the PTCs in a two-dimensional (2D) model of the RHD protein in Supplementary Fig. 5. Taken together, these results indicate that the RHD protein will be expressed in clone E4_M but not in E1_B or E4_B.

### Off-target mutations in the mutant clones

We investigated whether the *RHD*-mutant clones contain off-target mutations induced by the TALEN pairs. Potential off-target sites were predicted using a web-based programme called PROGNOS (Predicted Report Of Genome-wide Nuclease Off-target Sites)^21^ (Supplementary Data set 1) and the two top ranking sites for *RHD*_E1_TALENs and the three top ranking sites for *RHD*_E4_TALENs were chosen for further analysis. For both TALEN pairs, the highest ranking off-target sites were in the *RHCE* gene, which has high homology with the *RHD* gene^22^. The potential off-target site for the *RHD*_E1_TALENs in *RHCE* showed a perfect match with the target sequence; the T7E1 assay revealed mutations at that off-target site in the E1_B clone (Supplementary Fig. 6a). Sequencing of that region in the E1_B clone showed six sequences with a three-nucleotide deletion and four sequences with another three-nucleotide deletion, suggesting biallelic heterogeneous in-frame deletions at the *RHCE* locus (Supplementary Fig. 6b). The potential off-target site for the *RHD*_E4_TALENs in *RHCE* had a two base pair mismatch as compared with the target sequence; the T7E1 assay revealed mutations in E4_B but not E4_M (Supplementary Fig. 6c). Sequencing of that region in clone E4_B showed five wild-type sequences and five sequences with a 16-nucleotide deletion, indicating a monoallelic mutation (Supplementary Fig. 6d). Sequencing in clone E4 revealed 10 wild-type sequences, corroborating the absence of mutations in that region. In addition, in all three analysed clones, the T7E1 assay showed no mutations at the remaining other potential off-target sites outside of the *RHCE* gene (Supplementary Fig. 6a,c).

### *RHD* mRNA expression in mutant clones

**Figure 4:**
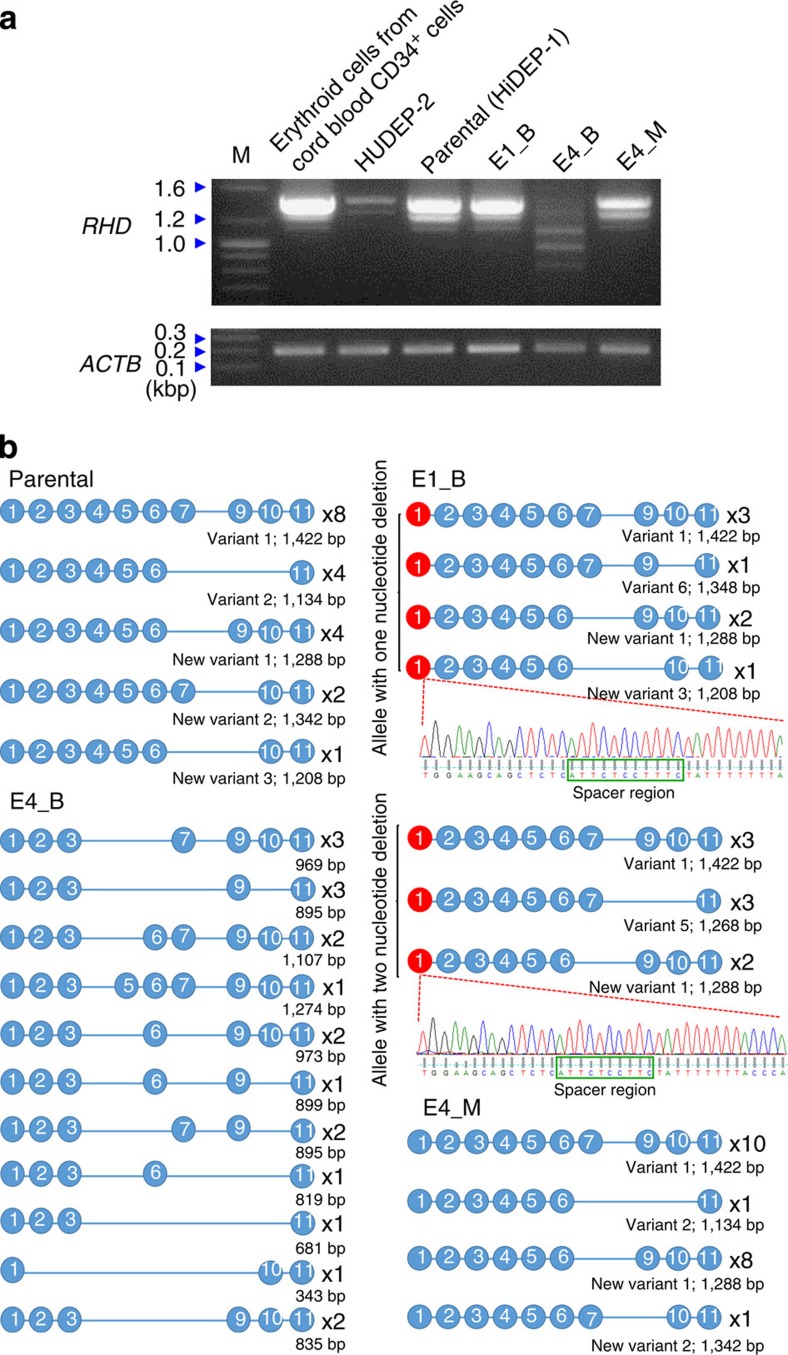
*RHD* mRNA in the mutated clones. RT–PCR was performed to detect *RHD* mRNA in each clone and the amplicons were subjected to electrophoresis (**a**) and sequencing (**b**). (**a**) Representative pictures of electrophoresis. *ACTB* was used as control. The sizes of marker (M) bands are shown on the left (kbp, kilobase pairs). (**b**) Schematic representation of *RHD* mRNA sequences. The number of occurrences is shown on the right of each transcript. Blue and red circles indicate normal and mutated exons, respectively. For some amplicons, the sequence and sequencing chromatogram are shown (spacer regions are indicated with green boxes).

We next examined *RHD* mRNA expression in parental, E1_B, E4_B and E4_M HiDEP-1 cells using RT–PCR. Electrophoresis of the RT–PCR products from parental HiDEP-1 cells showed a dense band at ∼1.4 kb, a medium-density band at ∼1.3 kb and a weak band at ∼1.1 kb (Fig. 4a). The two higher molecular weight bands were also clearly observed in E1_B and E4_M samples as well. To identify these bands, we cloned the RT–PCR products into T-vectors and performed capillary sequencing. The results showed that transcript variant 1, which lacks exon 8, and a new transcript variant 1, which lacks both exons 7 and 8, were observed at frequencies of 8/19 and 4/19 in parental cells, 6/15 and 4/15 in E1_B and 10/20 and 8/20 in E4_M, respectively, accounting for the two most frequently observed transcript variants in parental (8/19+4/19=63%), E1_B (6/15+4/15=67%) and E4_B (10/20+8/20=90%; Fig. 4b). Furthermore, the expected sizes of variant 1 and the new variant 1 are 1,422 and 1,288 bp, respectively. Taken together, these results indicate that the dense band at 1.4 kb and the medium-density band at 1.3 kb are variant 1 and new variant 1, respectively, and suggest that these two transcript variants are dominant in HiDEP-1 cells.

To determine whether these *RHD* mRNA variants are specific to HiDEP-1 cells, an erythroid cell line derived from induced pluripotent stem cells, we performed RT–PCR using RNA from two other kinds of erythroid cells: erythroid cells differentiated from primary human cord blood CD34^+^ cells^23^ and HUDEP-2 cells, an immortalized erythroid progenitor cell line derived from cord blood CD34^+^ cells^19^. Electrophoresis of the products from the two RT–PCRs showed the three bands, that is, a dense band at ∼1.4 kb, a medium-density band at ∼1.3 kb and a weak band at ∼1.1 kb, although the band at ∼1.1 kb was too faint to be observed in the HUDEP-2 mRNA (Fig. 4a). The similarity of the electrophoresis patterns of the RT–PCR products of the HiDEP-1 cells and the two different erythroid-lineage cells derived from CD34^+^ cells suggests that the *RHD* mRNA variants observed in the parental clone are not specific to HiDEP-1 cells. Furthermore, sequencing of these RT–PCR products showed that the *RHD* mRNA variants observed in the parental and E1_B and E4_M clones were also observed in both cell types derived from CD34^+^ cells at comparable frequencies (Supplementary Fig. 7), corroborating that *RHD* mRNA variants are not specific to cells derived from induced pluripotent stem cells.

These two bands, however, were not observed in the electrophoresis of E4_B RT–PCR products, which instead showed three weak, wide bands with a size range of ∼0.7∼1.1 kb. Sequencing of E4_B *RHD* transcripts revealed 11 transcript variants that were not observed in the other cells and have not been previously reported. Interestingly, all 11 observed transcript variants lack exon 4, the site where TALEN-induced mutations were induced. Taken together, the results indicate that the two mutations in exon 4 in clone E4_B lead to an alteration in *RHD* RNA splicing coupled with a reduced mRNA level.

In parental, E1_B, and E4_M HiDEP-1 cells, sequencing disclosed new transcript variants that have not been previously reported; these new variants are referred to as new transcript variants 1, 2 and 3, respectively, in this manuscript. In addition, sequencing also revealed that the TALEN-induced mutation in exon 1 of each *RHD* allele in E1_B was observed at frequencies of 8/15 and 7/15, respectively, indicating that the mRNAs are transcribed from both alleles and faithfully contain the mutation from each allele in the genomic DNA.

Electrophoresis showed that the RT–PCR products from E4_M (two weak bands at 1.4 and 1.3 kb and a very faint band at 1.1 kb) were similar in size to those from unmodified cells; however, all band intensities in the E4_M samples were proportionally weaker. Sequencing showed that the transcript variant expression pattern in E4_M was similar to that in unmodified cells and that the mutated sequence of one *RHD* allele in E4_M was not observed in mRNA. These results suggest that the majority, if not all, of the observed *RHD* mRNA in E4_M is expressed from the unmodified allele.

### Absence of D antigen in the *RHD*-knockout clones

**Figure 5:**
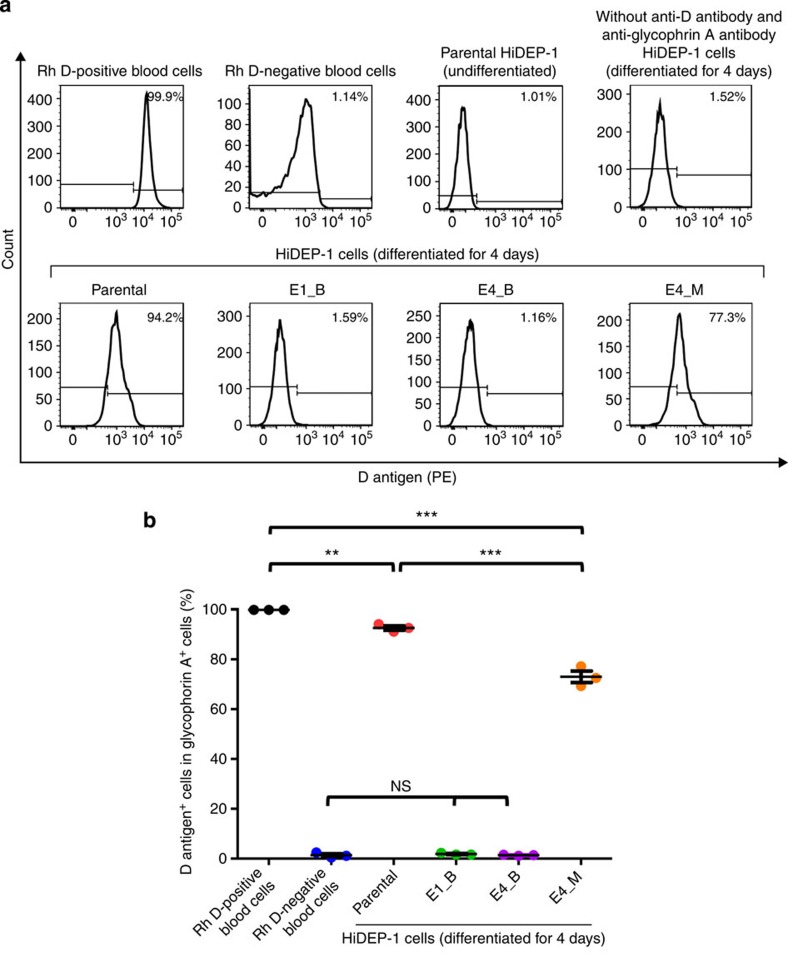
Flow cytometric analysis of D antigen expression in mutated cells. Parental and *RHD*-mutated (biallelic, E1_B, E4_B; monoallelic, E4_M) HiDEP-1 cells were induced for differentiation for 4 days and subjected to flow cytometry. D antigen expression was determined in glycophorin A^+^ cells. (**a**) Representative histograms. (**b**) The percentage of D antigen-positive cells in the population of glycophorin A-positive cells. ANOVA followed with Bonferroni's multiple comparison was performed (***P<0.001, **P<0.01, ns=not significant; *n*=3). Error bars represent the s.e.m.

We next evaluated D antigen protein expression on each HiDEP-1 cell clone using flow cytometry. As positive and negative controls for this analysis, we used peripheral blood cells isolated from Rh D-positive and -negative donors, respectively. Among glycophorin A^+^ cells, the D antigen expression rate was 99.96±0.004% and 1.4±0.6% in Rh D-positive and -negative blood cells, respectively (Fig. 5), suggesting that flow cytometry is a sensitive way to detect D antigen expression with an ∼1% nonspecific background signal.

Each clone was induced for differentiation towards erythrocytes for 4 days and subjected to flow cytometry. Most of the undifferentiated and differentiated HiDEP-1 cells were glycophorin A^+^ (Supplementary Fig. 8). Among the glycophorin A^+^ cells, the majority of cells in the parental and monoallelic mutant clone populations showed significant D antigen expression (Fig. 5; Parental clone: 93±0.9%, monoallelic mutant clone: 73±2%). However, the percentage of cells expressing D antigen in the monoallelic mutant clone was significantly lower than that in the parental clone (*P*<0.001, analysis of variance (ANOVA) followed by Bonferroni's multiple comparison), indicating the monoallelic mutation reduced D antigen expression. The D antigen expression in the parental clone was slightly, but significantly, lower than that in the Rh D-positive blood cells (*P*<0.01, ANOVA followed by Bonferroni's multiple comparison). The two *RHD* biallelic mutant clones expressed D antigen at a level that was similar to the nonspecific background level (E1_B, 1.8±0.2%; E4_B, 1.4±0.1%), suggesting the absence of D antigen expression in these TALEN-induced *RHD*-biallelic mutant clones.

### Agglutination test of the *RHD*-knockout clones

**Figure 6:**
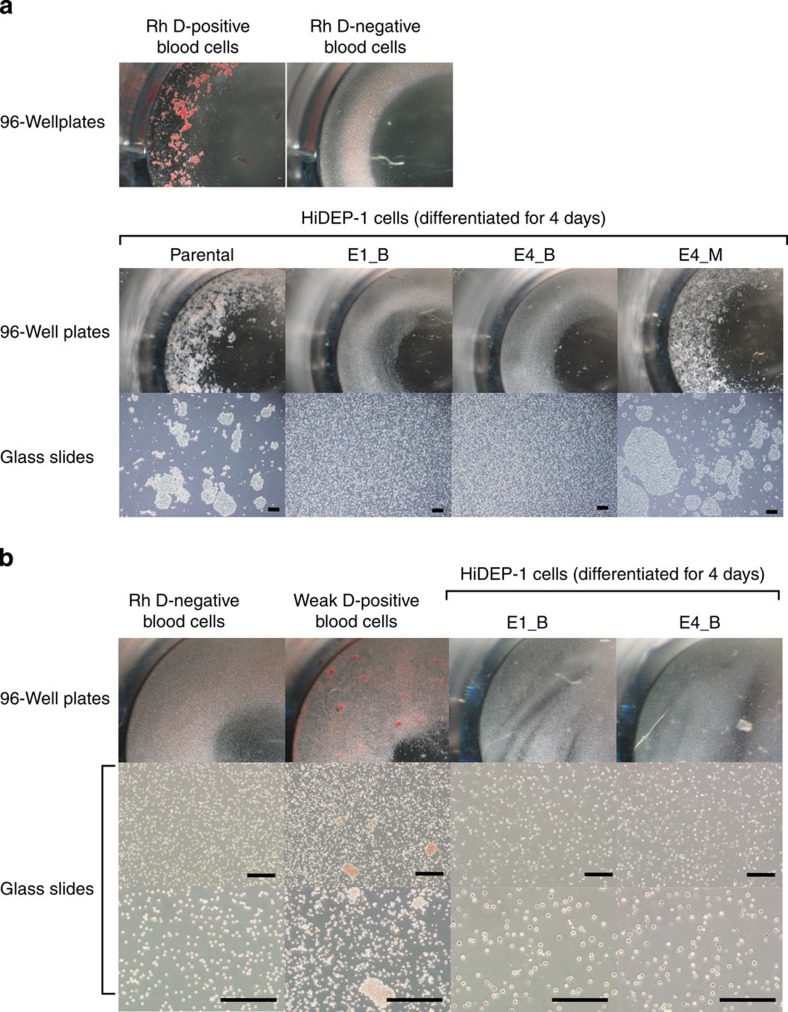
Absence of D antigen-mediated agglutination in *RHD*-knockout cell lines. Parental, *RHD*-knockout (E1_B, E4_B) and *RHD*-monoallelic mutant (E4_M) HiDEP-1 cells were induced for differentiation for 4 days and subjected to an agglutination test using anti-D blood grouping reagents (**a**) and a weak D test using anti-D blood grouping reagents and Coombs' reagent (Anti-IgG, -C3d) (**b**) in 96-well plates and on glass slides. Rh D-positive, D-negative and weak D-positive human peripheral blood cells were used as the controls. A photograph and photomicrographs of each cell line are shown. Scale bar, 500 μm.

We next performed an agglutination test, which is used for blood group typing. Parental, *RHD*-knockout (E1_B, E4_B) and *RHD*-monoallelic mutant (E4_M) HiDEP-1 cells were induced for differentiation for 4 days and subjected to the agglutination test using anti-D blood grouping reagents. The mixture of cells and anti-D reagent was incubated for 15 min at 37 °C, shaken with pipet tips and observed in 96-well plates and on glass slides. As positive and negative controls, we used Rh D-positive and -negative blood cells, which showed agglutination and no agglutination, respectively (Fig. 6a, Supplementary Fig. 9). Parental and E4_M, but not E1_B and E4_B, showed agglutination, suggesting that E1_B and E4_B are phenotypically Rh D-negative.

Weak D-positive cells, like D-negative cells, can also show ‘no agglutination' by the conventional agglutination testing described above. To rule out the possibility of weak D, we next performed a weak D detection test using E1_B and E4_B including Rh D-negative and weak D-positive blood cells as negative and positive controls, respectively. This test revealed no agglutination in either clone (Fig. 6b), demonstrating that the two *RHD*-knockout clones are D-negative rather than being weak D-positive.

### Differentiation and function of the *RHD*-knockout clones

**Figure 7:**
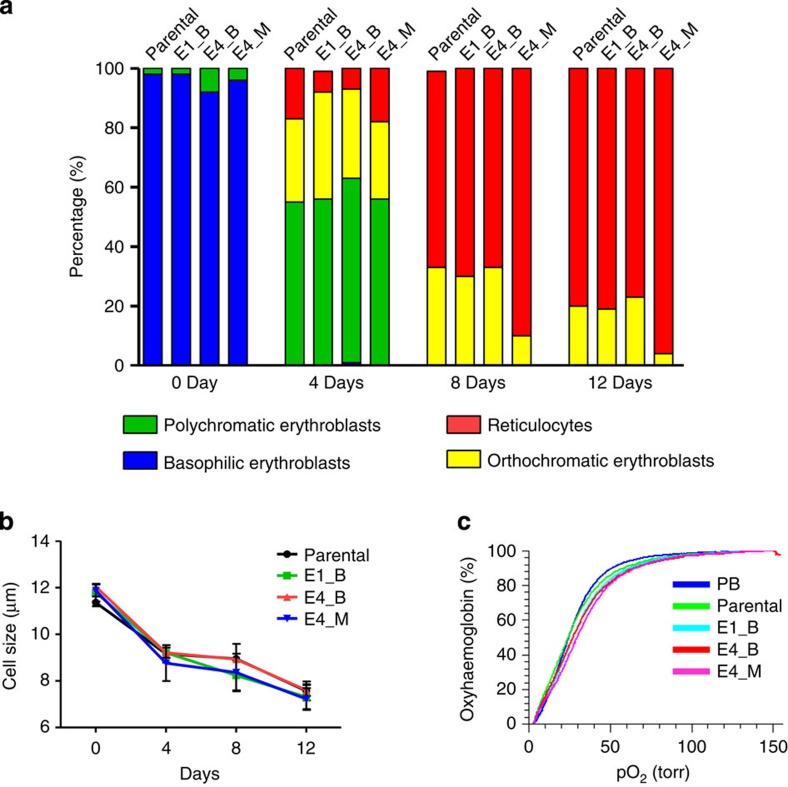
The *RHD* mutation does not affect the differentiation and function of HiDEP-1. (**a**,**b**) At the indicated times after the induction of erythrocyte differentiation, differentiation of each HiDEP-1 cell line was quantified using microscopy after Wright–Giemsa staining. (**a**) Morphological analysis of differentiation. The sum of two independent experiments is shown. (**b**) Cell size during differentiation. ANOVA showed that the cell sizes were the same in the parental and *RHD*-mutant clones. Error bars represent the s.e.m. *n*=3. (**c**) Oxygen equilibrium curves of parental and *RHD*-mutant clones (E1_B, E4_B and E4_M). Human adult peripheral blood (PB) cells were used as the control.

We evaluated whether TALEN-induced *RHD* knockout affects erythrocyte generation from the erythroid progenitor cells or the oxygen-carrying functions. Parental and E1_B cells were induced for differentiation into erythrocytes as previously reported by our group^19^. Morphological analysis showed that erythrocyte differentiation was comparable among parental, E1_B, E4_B and E4_M clones (Fig. 7a), suggesting that TALEN-induced *RHD* knockout does not affect this process. Furthermore, the average cell size during differentiation was similar among the parental and three mutant clones (Fig. 7b), supporting that *RHD* knockout does not influence differentiation. Moreover, flow cytometry showed that, after the induction of differentiation, the expression levels of erythroblast markers such as glycophorin A and CD71 were similar between the parental and three mutant clones (Supplementary Fig.10), corroborating that *RHD* knockout does not affect differentiation. For a functional comparison between the parental line and three mutant clones, we measured their oxygen-carrying abilities. The oxygen binding and dissociation curves obtained were similar for each of these cell types (Fig. 7c), indicating that the TALEN-induced *RHD* mutation does not affect red blood cell function. Taken together, these results indicate that erythrocyte differentiation and oxygen-carrying functions are not affected by the TALEN-induced *RHD* mutation.

## Discussion

Conversion of other blood groups into the O Rh D-negative group has been studied in transfusion medicine for many years because universal donor blood cells can be transfused into patients when blood group-matched donor cells are not available or in an emergency situation when blood group determination cannot be readily achieved. The conversion of the A or B blood group into O has been accomplished using enzymatic reactions because ABO blood group determination depends on glycosyltransferase activity, the results of which can be reversed using available exoglycosidases^1^,^2^,^3^,^4^. However, similar enzymatic manipulations are not applicable to Rh D blood group conversion because the Rh D blood group is determined by antigenic RHD protein expression, not by the activity of certain enzymes. Here we showed that TALEN-based disruption of *RHD* can convert homozygous dominant Rh D-positive cells into D-negative cells.

The main achievement of programmable nuclease-induced gene knockout to date has been the determination of the functions of genes^24^,^25^,^26^ and noncoding elements^27^,^28^,^29^ in cells or organisms. In addition, such gene disruption has also been utilized to treat human immunodeficiency virus infection^30^,^31^,^32^. Here, for the first time, we expanded the application of programmable nuclease-mediated gene disruption to blood group conversion.

Given that the different types of programmable nucleases share genome-editing abilities and mechanisms^13^, we can assume that conversion into Rh D-negative cells could be achieved using other programmable nucleases such as ZFNs and RGENs. Furthermore, our study also raises the possibility that programmable nuclease-mediated gene editing could lead to blood group conversion in other blood group systems as well. For example, as the ABO blood group is determined by *ABO*, programmable nuclease-mediated knockout of *ABO* could lead to the generation of O blood-type cells from A, B or AB blood-type cells. Programmable nuclease-directed double knockout of *ABO* and *RHD* in erythroid progenitor cells or haematopoietic stem cells would enable the generation of O Rh D-negative blood type, universal donor blood cells.

In the E1_B and E4_B clones, the absence of D antigen expression was validated using flow cytometry and agglutination assays. For E1_B, given that a significant amount of *RHD* mRNA was expressed and that the mRNAs contain PTCs in exon 1, the absence of D antigen would be attributable to PTC-induced translational termination or nonsense-mediated translational repression^33^,^34^. This lack of D antigen expression in the presence of a significant level of *RHD* mRNA in E1_B is in contrast to the significant D antigen expression and reduced mRNA level in E4_M. The reduction of the *RHD* mRNA level in E4_M would be attributable to a lack of mRNA expression from the mutated allele, given that mRNA containing the mutation in exon 4 was not detected using mRNA sequencing. As the mechanism underlying this lack of mRNA from the mutated allele, we cannot rule out nonsense-mediated decay, although the mutation-induced PTC was 41 nucleotides upstream of the exon 4 terminus, which does not satisfy the ‘50–55 nucleotide rule'^35^,^36^,^37^,^38^,^39^. In E4_B, aberrant splicing and a decrease in mRNA expression was observed. The aberrant splicing might be caused by mutation themselves or by nonsense-mediated alternative splicing, which was induced by PTCs that were generated by mutations. Given that the PTCs generated by the two mutations in E4_B cells satisfy the ‘50–55 nucleotide rule', the decrease in mRNA could be attributable to nonsense-mediated decay.

Although the D antigen was not detected using flow cytometry and agglutination assays in this study, we cannot rule out the possibility that our approach could lead to partial RHD protein expression. This partially expressed protein could still be antigenic, which could cause clinical problems, but might be hard to detect using antibody-mediated analyses such as flow cytometry and agglutination assays. In some cases, nuclease-induced mutations could create new epitopes as reported in previous studies in which mutations led to partial D expression^40^,^41^. Regarding such partial protein expression, genetic analysis or genomic DNA sequencing can reduce the false-negative rate of serological analysis^42^. If PTCs are in the transmembrane domain encoded by the *RHD* exon 1, expression of the extracellular region would be absent and, thus, the resulting protein would not cause clinical problems. In clone E1_B of this study, the PTCs are in the extracellular domain encoded by exon 1 and the resulting expected extracellular domain is only two and six-amino acids in length (Supplementary Fig. 6); such short regions have a low, if not zero, possibility of serving as antigens. For clone E4_B, we cannot rule out the possibility of potentially problematic partial expression of the first to third RHD extracellular domains because the PTCs are in exons 4 and 5. Thus, the possibility of problematic partial protein expression may be reduced by the proper selection of clones, in this case by choosing clone E1_B instead of E4_B.

Compared with the previously reported enzymatic digestion of antigenic carbohydrate as a method for converting other blood groups into group O^1^,^2^,^3^,^4^, our approach has advantages and disadvantages. Our approach can be applied to progenitor or stem cells that can give rise to numerous erythrocytes. *In vivo* or *ex vivo* application of programmable nucleases to haematopoietic stem cells would lead to the generation of an almost unlimited number of erythrocytes with a converted blood group. However, such gene editing cannot be applied to enucleated mature erythrocytes, in contrast to enzymatic digestion. Given that the mechanisms for blood group conversion are different between the two approaches, they could potentially be combined to create a very effective system for converting blood groups.

The absence or extremely suppressed expression of Rh antigens can lead to Rh_null_ or Rh_mod_ disease, which is characterized with a mild to moderate haemolytic anaemia associated with stomatocytosis and plasma membrane abnormalities^22^,^43^. Because most of the differentiated cells in this study were reticulocytes, we could not determine whether *RHD* mutations cause stomatocytosis, although we did not observe any stomatocytes in the differentiated parental and mutant clones. Rh_null_ or Rh_mod_ disease can be caused by *RHAG* mutations alone or *RHCE* mutations combined with an *RHD* deletion^22^. Using the two TALEN pairs in this study, the possibility of generating the *RHAG* mutation would be extremely low because potential off-target sites were not found in *RHAG*. In the *RHCE* gene, potential off-target sites were found for both TALEN pairs. However, biallelic mutations in *RHCE* were found in only one clone out of the three *RHD*-mutant clones. These biallelic mutations were both in-frame, leading to minor changes (that is, one and two amino acid deletions) in Rh C, e, E or e antigens. Taken together, these observations suggest that the possibility of Rh_null_ or Rh_mod_ disease being caused by our approach would be low, although we cannot rule it out. To prevent such a disease, we can select *RHD*-mutant clones that do not have mutations in *RHCE* or use programmable nucleases that do not have off-target effects on *RHCE*.

Here we used Rh D-positive cells as a source for erythroid progenitor cells. Instead, one could use cells derived from an Rh D-negative donor as the source for erythroid progenitor cells, which would not necessitate the programmable nuclease-mediated blood group conversion. However, for some ethnic groups, the availability of Rh D-negative donors matched for other minor antigens could be much lower than that of Rh D-positive donors matched for other minor antigens. Furthermore, the genetic diversity of the RH locus exceeds all estimates predicted by serology^44^, and Rh serologic phenotype-matched transfusion from minority donors does not prevent all cases of Rh alloimmunization in patients with sickle cell disease^45^. Thus, our approach of Rh antigen knockout would be a useful means of preventing alloimmunization.

In summary, here we showed that TALENs can induce the conversion of D-positive cells into D-negative cells by disrupting *RHD*, expanding the application of programmable nucleases into blood group conversion. This nuclease-based blood group gene editing opens new avenues for the development of methods for converting blood groups in transfusion medicine.

## Methods

### Cell culture

The Rh-positive blood group (homozygous dominant (DD)) erythroid progenitor cell lines HiDEP-1 and HUDEP-2 were derived from human-induced pluripotent stem cells and human umblical cord blood cells, respectively^19^, and are available from Dr Yukio Nakamura yukionak@brc.riken.jp) on request. We maintained these cells in a serum-free StemSpan SFEM medium (StemCell Technologies, Vancouver, BC, Canada), without feeder cells in the presence of doxycycline (1 μg ml^−1^; Sigma, St Louis, MO, USA), erythropoietin (EPO; 3 IU ml^−1^; CJ Phama, Seoul, South Korea) and dexamethasone (10^−6^ M; Sigma). Cord blood was collected from normal full-term deliveries in bags containing 24.5 ml of citrate phosphate dextrose A (Green Cross Corp., Yong-in, Korea) under the approval of the Severance Hospital Institutional Review Board. Red blood cells were removed using Ficoll-PaqueTM PLUS (density, 1.077; GE Healthcare, Uppsala, Sweden) according to the manufacturer's instructions. CD34^+^ cells were isolated using a CD34 MicroBead Kit (Miltenyi Biotech, Bergisch Gladbach, Germany) according to the manufacturer's instructions. Cord blood CD34^+^ cells were induced to differentiate into the erythroid lineage for 17 days as follows^23^. From day 0 to 7, sorted CD34^+^ cells were continually cultured in serum-free culture medium supplemented with 100 ng ml^−1^ stem cell factor (Peprotech, Rehovot, Israel), 10 ng ml^−1^ interleukin-3 (Peprotech) and 6 IU ml^−1^ recombinant EPO (Recormon Epoetin beta, Roche Diagnostic) with a half-volume medium change twice a week. Serum-free culture medium consisted of StemPro-34 SFM Complete Medium supplemented with 1% bovine serum albumin (Sigma), 150 μg ml^−1^ iron-saturated human transferrin (Sigma), 50 μg ml^−1^ insulin (Sigma), 90 ng ml^−1^ ferrous nitrate (Sigma), 2 mM l-glutamine (Sigma), 1.6 × 10^−4^ M monothioglycerol (Sigma), 30.8 μM vitamin C (Sigma), 2 μg ml^−1^ cholesterol (Sigma) and 1% penicillin–streptomycin solution (Gibco). In the second 7-day period of culture, the medium was replaced with serum-free culture medium with 3 IU ml^−1^ recombinant EPO, 50 ng ml^−1^ stem cell factor and 10 ng ml^−1^ interleukin-3 to induce cell expansion and differentiation. During days 15 to 17 of culture, only one cytokine (EPO, at 2 IU ml^−1^) was used for erythrocyte differentiation, and poloxamer 188 (Pluronic F68 (F68), Sigma; MW 8,400) was added at a concentration of 0.05%. HEK293T (human embryonic kidney cell line) cells were cultured in DMEM (Gibco-BRL) supplemented with 10% fetal bovine serum (FBS, Gibco-BRL). Rh D-positive and Rh D-negative peripheral blood cells were supplied by Severance Hospital Blood Bank (Seoul, South Korea).

### TALENs targeting *RHD*

TALENs targeting exons 1 and 4 of the *RHD* gene were designed to include a 5′ thymine as the starting point of the TALE-binding sites^26^ (http://www.talenlibrary.net/); the plasmids encoding these TALENs were obtained from ToolGen (Seoul, South Korea). The maps and sequences of the plasmids are shown in Supplementary Note 1. These plasmids are available from Addgene, TALEN library resource (http://www.talenlibrary.net) and ToolGen (Order number H172187, H172190). The repeat variable diresidues used were NI, NN, NG and HD for binding to adenine, guanine, thymine and cytosine in the target sequence, respectively. The magnetic reporters were prepared as previously described^15^. To evaluate TALEN activity, 3.0 μg of TALEN plasmids (a plasmid encoding one TALEN and a plasmid encoding the other TALEN, each 1.5 μg) were transfected into 293T cells using FuGENE HD Transfection Reagent (Roche Diagnostics, Basel, Switzerland). After 72 h of transfection, cells were harvested and subjected to the T7E1 assay.

### Generation of *RHD*-knockout clones

HiDEP-1 cells were transfected with a total 4.5 μg of TALEN plasmids and reporters (1.5 μg plasmid encoding one TALEN, 1.5 μg plasmid encoding the other TALEN and 1.5 μg magnetic reporter plasmid) using a Human CD34 cell nucleofector kit (Lonza, Basel, Switzerland) from the Amaxa system. After incubation for 3 days at 37 °C, the transfected HiDEP-1 cells were mixed with magnetic bead-conjugated antibody against H-2K^k^ (MACSelect K^k^ microbeads; Miltenyi Biotech) and incubated for 20 min at 4 °C. H-2K^k+^ cells were separated on a column (MACS LS column; Miltenyi Biotech) according to the manufacturer's instructions and seeded into 96-well plates at 0.25 cell per well. The cells were cultured for 2 weeks and each obtained clone was transferred into 24-well plates for further expansion. *RHD*-knockout clones were identified using the T7E1 assay and sequencing.

### T7E1 assay

Genomic DNA was isolated using a Genomic DNA purification Kit (Promega, Fitchburg, WI, USA) according to the manufacturer's instructions. Owing to the high homology between *RHD* and *RHCE*, we performed nested PCR using primers that bind specifically to *RHD* but not *RHCE* (Supplementary Table 1). The PCR amplicons were denatured by heating, annealed to form heteroduplex DNA, treated with 5 units of mismatch-sensitive T7 endonuclease 1 (New England Biolabs, Hitchin, UK) for 20 min at 37 °C and analysed by 2% agarose gel electrophoresis. Mutation frequencies were calculated as previously described on the basis of the band intensities using the Image J software and the following equation^46^: mutation frequency (%)=100 × [1−(1−fraction cleaved)^1/2^], where the fraction cleaved is the total relative density of the cleavage bands divided by the sum of the relative density of the cleavage bands and uncut bands. Uncropped images of gels shown in Figs 1b,c and 2b and Supplementary Figs 3 and 6a,c are shown in Supplementary Figs 11a,b,12,13 and 14a,b, respectively.

### Sequencing analysis

PCR amplicons were purified using a Fragment DNA Purification Kit (iNtRON biotechnology, Seongnam, South Korea) and cloned into the pGEM-T vector. Cloned plasmids were sequenced using a T7 primer (Supplementary Table 1).

### RT–PCR of *RHD* mRNA

Total RNA was extracted from cells using TRIzol reagent (Invitrogen, Carlsbad, CA, USA) according to the manufacturer's instructions and subjected to reverse transcription using Oligo-dT primer (Qiagen, Hilden, Germany) and AccuPower RT PreMix (Bioneer, Daejeon, South Korea) according to the manufacturer's instructions. The synthesized cDNA was subjected to PCR using a pair of primers (Supplementary Table 1). The PCR amplicons were analysed with 1% agarose gel electrophoresis and sequenced as described above. Uncropped images of gels shown in Fig. 4a are shown in Supplementary Fig. 15.

### Induction of differentiation into erythrocytes

Differentiation into erythrocytes was induced by culturing the HiDEP-1 cells at 1 × 10^6^ cells ml^−1^ in erythroid differentiation medium; IMDM (Gibco-BRL) containing 10% human AB serum (Sigma), α-tocopherol (20 ng ml^−1^; Sigma), linoleic acid (4 ng ml^−1^; Sigma), cholesterol (200 ng ml^−1^; Sigma), sodium selenite (2 ng ml^−1^; Sigma), holo-transferrin (200 ng ml^−1^; Sigma), human insulin (10 μg ml^−1^; Sigma), D-mannitol (14.57 mg ml^−1^; Sigma), mifepristone (an antagonist of glucocorticoid receptor, 1 μM; Sigma), and EPO (5 IU ml^−1^)^19^.

### Flow cytometry

Harvested cells were incubated with anti-D blood grouping reagent (human IgG/IgM monoclonal; Millipore, Billerica, MA, USA; dilution 1:10), antigen-presenting cell-conjugated anti-human-CD235a (BD Biocsciences, Franklin Lakes, NJ, USA; dilution 1:100) and phycoerythrin-conjugated anti-human IgG (Fc γ-specific; eBioscience, San Diego, CA, USA; dilution 1:100) in 100 μl of staining medium (PBS (Sigma) containing 2% FBS) for 20 min at room temperature. Stained cells were analysed using FACS Cantos II (BD Biosciences) and FlowJo (version X.0.7).

### Morphological analysis

Cells (1 × 10^5^ cells per slide) were centrifuged on slides using cytocentrifuge (Cytospin 4, Thermo Scientific, Waltham, MA, USA; 113 g (1,000 r.p.m.), 5 min), stained with Wright–Giemsa dye (Sigma) and observed using a microscope (BX51; OLYMPUS, Tokyo, Japan) and a camera (DP71; OLYMPUS).

### Agglutination assay

The differentiated HiDEP-1 cells and control red blood cells were plated into 96-well plates at 1 × 10^6^ cells per well in 10 μl of PBS. Then, we added 10 μl of anti-D blood grouping reagent to each well and incubated the cells for 15 min at 37 °C. Incubated cells were stirred using pipette tips and observed using a stereo zoom microscope (SZ61; OLYMPUS) and a camera (G10; Canon, Tokyo, Japan). For observation at higher magnification, the cell mixture was transferred to glass slides and observed using an inverted microscope (IX71; OLYMPUS) and a camera (DP70; OLYMPUS).

Clones that do not agglutinate in the presence of anti-D blood grouping reagents were subjected to a weak D test. We added 10 μl of anti-D blood grouping reagent to Eppendorf tubes containing 2 × 10^6^ cells in 10 μl PBS and incubated the mixture for 15 min at 37 °C. After washing three times with PBS, the cells were mixed with 10 μl anti-human IgG, -C3d (Ortho-Clinical Diagnostics, High Wycombe, UK), plated into 96-well plate, centrifuged for 30 s at 198*g* (1,000 r.p.m.) and observed as described above.

### Functional analysis of haemoglobin

After washing with PBS, 5 × 10^7^ cells per group were subjected to the analysis. Oxygen equilibrium curves were determined using a Hemox-Analyzer (TSC scientific, New Hope, PA, USA) according to the manufacturer's instructions^23^. Human adult peripheral blood cells were used as the control.

### Statistical analysis

All data were expressed as means±s.e.'s. Statistical analysis was conducted using IBM SPSS Statistics (version 21; IBM corporation, Armonk, NY, USA). ANOVA followed by multiple comparison with Bonferroni's method was performed. A *P* value<0.05 was considered statistically significant.

## Additional information

**How to cite this article:** Kim, Y.-H. *et al.* Rh D blood group conversion using transcription activator-like effector nucleases. *Nat. Commun.* 6:7451 doi: 10.1038/ncomms8451 (2015).

## Supplementary Material

Supplementary Information Supplementary Figures 1-15, Supplementary Table 1, Supplementary Note 1 and Supplementary Reference
