# Corrigendum: Rh D blood group conversion using transcription activator-like effector nucleases

**DOI:** 10.1038/ncomms9678

**Published:** 2015-10-22

**Authors:** Young-Hoon Kim, Hyun O. Kim, Eun J. Baek, Ryo Kurita, Hyuk-Jin Cha, Yukio Nakamura, Hyongbum Kim

*Nature Communications* 6: Article number: 745110.1038/ncomms8451 (2015); Published: 06162015; Updated: 10222015

The original version of the Supplementary Information attached to this Article contained errors in the sequences of plasmids presented in Supplementary Note 1. The HTML has now been updated to include a corrected version of the Supplementary Information.

## 

## Supplementary Material

Supplementary Information Supplementary Figures 1-15, Supplementary Table 1, Supplementary Note 1 and Supplementary Reference.

