# Supplementary material for: Corrigendum: Rh D blood group conversion using transcription activator-like effector nucleases
Source: Nat Commun. 2015 Oct 22;6:8678. doi: 10.1038/ncomms9678 (PMC4644981; doi:10.1038/ncomms9678)
Supplement: Supplementary file 1 [file ncomms9678-s1.pdf]

Supplementary information

Rh D blood group conversion using transcription activator-like effector nucleases

Young-Hoon Kim[1,2], Hyun Ok Kim[3], Eun Jung Baek[4], Ryo Kurita[5], Hyuk-Jin Cha[6], Yukio Nakamura[7], Hyongbum Kim[1,2]

[1]Graduate School of Biomedical Science and Engineering/college of Medicine, Hanyang University, Seoul, 133-791, South Korea, [2]Department of Pharmacology, Brain Korea 21 Plus Project for Medical Sciences, Yonsei University College of Medicine, Seoul, 120-752, South Korea, [3]Department of Laboratory Medicine, Yonsei University College of Medicine, Seoul, 120-752, South Korea, [4]Department of Laboratory Medicine, Hanyang University College of Medicine, Seoul, 133-791, South Korea, [5]Department of Research and Development, Central Blood Institute, Japanese Red Cross Society, Tokyo,135-8521, Japan, [6]Department of Life Sciences, College of Natural Sciences, Sogang University, Seoul, 121-742, Korea, [7]Cell Engineering Division, RIKEN BioResource Center, Tsukuba, Ibaraki, 305-0074, Japan.

Supplementary information:

Supplementary Figures 1 – 15

Supplementary Table 1

Supplementary Note 1

Supplementary Reference

Legend for Supplementary Dataset 1

Supplementary Figure 1. The reported transcript variants of the *RHD* gene

The transcript variants of *RHD* reported in NCBI and Ensembl are shown with their accession numbers and transcript IDs, respectively, on the right. All of the variants have the start codon in exon 1 except variant 3, which has the start codon in exon 4. Blue circles and black boxes indicate exons and coding sequence (CDS), respectively.

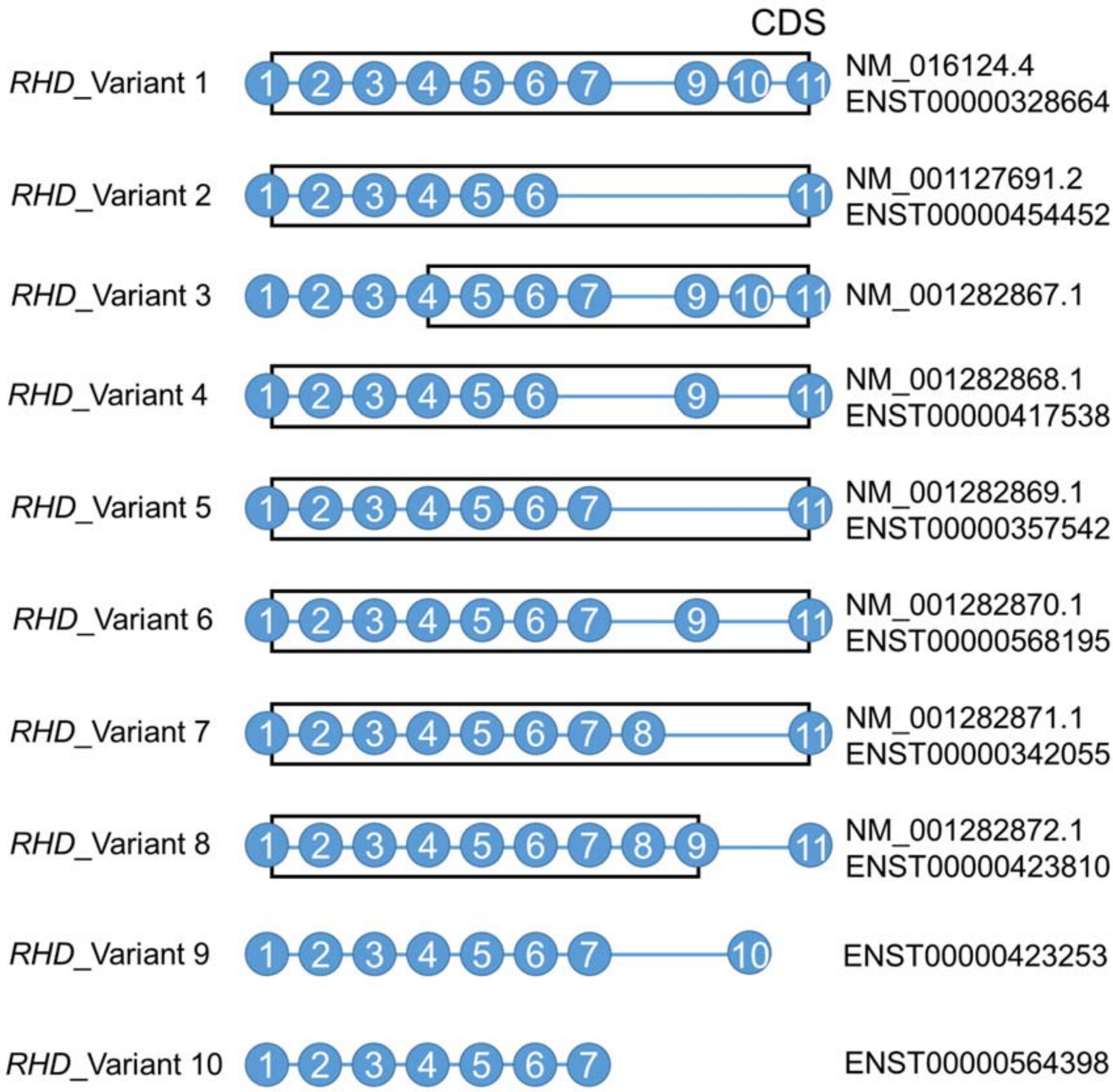

Supplementary Figure 2. The structure and working mechanism of the episomal reporters

mRFP is constitutively expressed by the CMV promoter (P_{CMV}), whereas eGFP and H-2K^k are not expressed without the activity of TALENs because i) the sequences are out of frame and ii) there is a stop codon before the eGFP gene. When a double-strand break is introduced into the target sequence by TALENs, the break is repaired by nonhomologous end-joining (NHEJ) which often results in indels. Indel generation can cause frame shifts, which can render eGFP and H-2K^k genes in frame and lead to the expression of eGFP and H-2K^k.

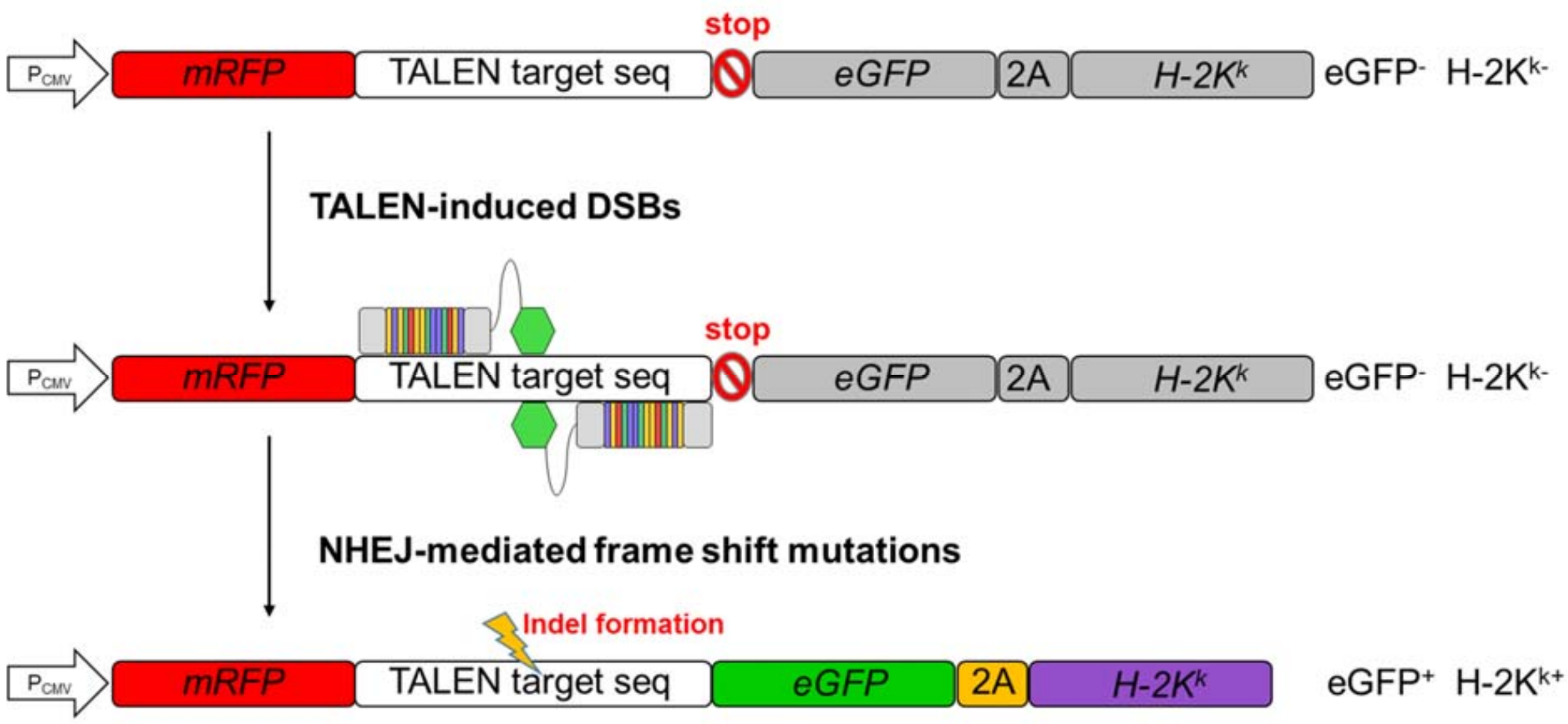

Supplementary Figure 3. T7E1 assay to screen mutant clones

Clonal culture of HiDEP-1 erythroid progenitor cells was initiated three days after transfection with plasmids encoding TALENs that target *RHD*. Genomic DNA from each clone was analyzed 17 days after the initiation of clonal culture. Genomic DNA from four clones was mixed at a 1:1:1:1 weight ratio and subjected to the T7E1 assay to determine whether at least one of the four clones contains mutations. The identities of the clones in each DNA mixture are shown. Arrows indicate the expected position of DNA bands cleaved by T7E1. Genomic DNA mixtures containing mutations at the target sites are marked with red clone numbers. Untransfected cells and a cell population transfected with the TALENs were used as the negative control (NC) and positive control (PC), respectively. The sizes of marker (M) bands are shown on the left (Kbp, kilobase pairs).

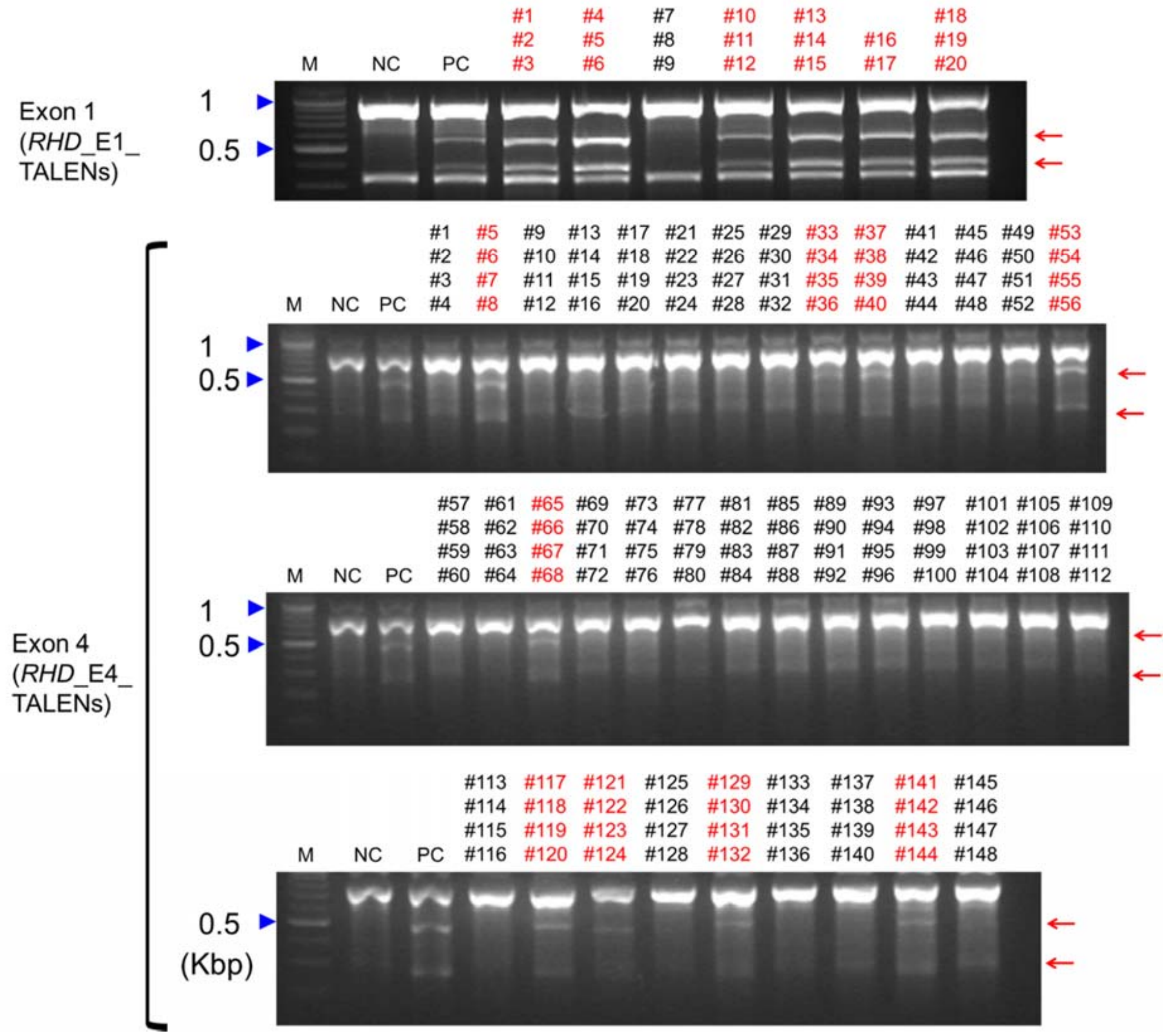

Supplementary Figure 4. DNA sequences of *RHD*-mutated clones

HiDEP-1 cells were co-transfected with the reporter plasmid and plasmids encoding TALENs that target *RHD* exon 1 (a, b) or exon 4 (c). After magnetic separation, cells were subjected to clonal culture. Genomic DNA was isolated from each clone, PCR-amplified, and cloned into T vectors; individual cloned amplicons were then subjected to sequencing. The *RHD* gene DNA sequences in the wild-type cells and selected clones (#number) are shown with TALE binding sequences in a red font. Clone #7 (a) was subjected to another cycle of transfection, magnetic separation, and clonal culture; subclones derived from clone #7 are indicated by a number following #7 (b). Deleted bases are indicated by dashes and inserted bases are shown in a blue font. The number of occurrences is shown in parentheses (e.g., x2 and x4 indicate the number of each sequence). Clones that were selected for further experiments are indicated with the clone name in a red color.

a Exon 1

```
    GGGCCCTAACACTGGAAGCAGCTCTCATTCTCCTCTTCTATTTTTTTACCCACTATGACGCTTC (Wild type)

#1  GGGCCCTAACACTGGAAGCAGCTCTCATTCTCCTCTTCTATTTTTTTACCCACTATGACGCTTC (x2)  ← Wild type
    GGGCCCTAACACTGGAAGCAGCTCTCAT--TCCTCTTCTATTTTTTTACCCACTATGACGCTTC (x3)  ← 2nt deletion; out of frame

#3  GGGCCCTAACACTGGAAGCAGCTCTCATTCTCCTCTTCTATTTTTTTACCCACTATGACGCTTC (x3)  ← Wild type
    GGGCCCTAACACTGGAAGCAGCTCTCATTCT--TCTTCTATTTTTTTACCCACTATGACGCTTC (x2)  ← 2nt deletion; out of frame

#4  GGGCCCTAACACTGGAAGCAGCTCTCATTCTCC------ATTTTTTTACCCACTATGACGCTTC (x3)  ← 6nt deletion; inframe
    GGGCCCTAACACTGGAAGCAGCTCTCATTCTC--CTTCTATTTTTTTACCCACTATGACGCTTC (x2)  ← 2nt deletion; out of frame

#6  GGGCCCTAACACTGGAAGCAGCTCTCATTCTCCTCTTCTATTTTTTTACCCACTATGACGCTTC (x1)  ← Wild type
    GGGCCCTAACACTGGAAGCAGCTCTCAT-----TCTTCTATTTTTTTACCCACTATGACGCTTC (x11) ← 5nt deletion; out of frame

#11 GGGCCCTAACACTGGAAGCAGCTCTCATTCTCCTCTTCTATTTTTTTACCCACTATGACGCTTC (x2)  ← Wild type
    GGGCCCTAACACTGGAAGCAGCTCTCAT-----TCTTCTATTTTTTTACCCACTATGACGCTTC (x3)  ← 5nt deletion; out of frame

#15 GGGCCCTAACACTGGAAGCAGCTCTCATTCTCCTCTTCTATTTTTTTACCCACTATGACGCTTC (x3)  ← Wild type
    GGGCCCTAACACTGGAAGCAGCTCTCATTCTCC---TCTATTTTTTTACCCACTATGACGCTTC (x2)  ← 3nt deletion; inframe

#17 GGGCCCTAACACTGGAAGCAGCTCTCATTCTCCTCTTCTATTTTTTTACCCACTATGACGCTTC (x1)  ← Wild type
    GGGCCCTAACACTGGAAGCAGCTCTCATTCTC--CTTCTATTTTTTTACCCACTATGACGCTTC (x4)  ← 2nt deletion; out of frame

#19 GGGCCCTAACACTGGAAGCAGCTCTCATTCTCCTCTTCTATTTTTTTACCCACTATGACGCTTC (x2)  ← Wild type
    GGGCCCTAACACTGGAAGCAGCTCTCATTCTCCTC--CTATTTTTTTACCCACTATGACGCTTC (x2)  ← 2nt deletion; out of frame

#20 GGGCCCTAACACTGGAAGCAGCTCTCATTCTCCTCTTCTATTTTTTTACCCACTATGACGCTTC (x3)  ← Wild type
    GGGCCCTAACACTGGAAGCAGCTCTCA--------TTCTATTTTTTTACCCACTATGACGCTTC (x1)  ← 8nt deletion; out of frame
```

b Exon 1

```
GGGCCCTAACACTGGAAGCAGCTCTCATTCTCCTCTTCTATTTTTTTACCCACTATGACGCTTC (Wild type)

#17-1
GGGCCCTAACACTGGAAGCAGCTCTCATTCTCCTCTTCTATTTTTTTACCCACTATGACGCTTC (x2) ← Wild type
GGGCCCTAACACTGGAAGCAGCTCTCATTCTC--CTTCTATTTTTTTACCCACTATGACGCTTC (x4) ← 2nt deletion; out of frame

#17-2
GGGCCCTAACACTGGAAGCAGCTCTCATTCTCCTCTTCTATTTTTTTACCCACTATGACGCTTC (x2) ← Wild type
GGGCCCTAACACTGGAAGCAGCTCTCATTCTC--CTTCTATTTTTTTACCCACTATGACGCTTC (x3) ← 2nt deletion; out of frame

#17-3 (E1_B)
GGGCCCTAACACTGGAAGCAGCTCTCATTCTCCT-TTCTATTTTTTTACCCACTATGACGCTTC (x7) ← 1nt deletion; out of frame
GGGCCCTAACACTGGAAGCAGCTCTCATTCTC--CTTCTATTTTTTTACCCACTATGACGCTTC (x5) ← 2nt deletion; out of frame

#17-4
GGGCCCTAACACTGGAAGCAGCTCTCATTCTCCTCTTCTATTTTTTTACCCACTATGACGCTTC (x1) ← Wild type
GGGCCCTAACACTGGAAGCAGCTCTCATTCTC--CTTCTATTTTTTTACCCACTATGACGCTTC (x4) ← 2nt deletion; out of frame

#17-5
GGGCCCTAACACTGGAAGCAGCTCTCATTCTCCTCTTCTATTTTTTTACCCACTATGACGCTTC (x2) ← Wild type
GGGCCCTAACACTGGAAGCAGCTCTCATTCTC--CTTCTATTTTTTTACCCACTATGACGCTTC (x4) ← 2nt deletion; out of frame

#17-6
GGGCCCTAACACTGGAAGCAGCTCTCATTCTCCTCTTCTATTTTTTTACCCACTATGACGCTTC (x3) ← Wild type
GGGCCCTAACACTGGAAGCAGCTCTCATTCTC--CTTCTATTTTTTTACCCACTATGACGCTTC (x3) ← 2nt deletion; out of frame

#17-7
GGGCCCTAACACTGGAAGCAGCTCTCATTCTCCTCTTCTATTTTTTTACCCACTATGACGCTTC (x2) ← Wild type
GGGCCCTAACACTGGAAGCAGCTCTCATTCTC--CTTCTATTTTTTTACCCACTATGACGCTTC (x4) ← 2nt deletion; out of frame
```

c Exon 4

```
TTGGGCTGTCTGTGGCCTGGTGCCTGCCAAAGCCTCTACCCGAGGGAACGGAGGATAAAGATCA (Wild type)

#5 (E4_B)
TTTCCTTCCACTCCACACACCCCTAAGACTCTGGATCCTGCGAGGGAACGGAGGATAAAGATCA (x2) ← 31nt deletion, 236nt insertion; out of frame
TTGGGCTGTCTGTGGCCTGGTGCCTGCCAAAG-CTCTACCCGAGGGAACGGAGGATAAAGATCA (x17) ← 1nt deletion; out of frame

#8 (E4_M)
TTGGGCTGTCTGTGGCCTGGTGCCTGCCAAAGCCTCTACCCGAGGGAACGGAGGATAAAGATCA (x1) ← Wild type
TTGGGCTGTCTGTGGCCT-----------------CTACCCAAGGGAACGGAGGATAAAGATCA (x5) ← 24nt deletion, 7nt insertion; out of frame

#33
TTGGGCTGTCTGTGGCCTGGTGCCTGCCAAAGCCTCTACCCGAGGGAACGGAGGATAAAGATCA (x2) ← Wild type
TTGGGCTGTCTGTGGCCTGGT---------------------------CGGAGGATAAAGATCA (x4) ← 27nt deletion; inframe

#37
TTGGGCTGTCTGTGGCCTGGTGCCTGCCAAAGCCTCTACCCGAGGGAACGGAGGATAAAGATCA (x3) ← Wild type
TTGGGCTGTCTGTGGCCTGGTGCCTGCCAAAGC------------GAACGGAGGATAAAGATCA (x3) ← 12nt deletion; inframe

#53
TTGGGCTGTCTGTGGCCTGGTGCCTGCCAAAGCCTCTACCCGAGGGAACGGAGGATAAAGATCA (x3) ← Wild type
TTGGGCTGTCTGTGGCCTGGTGCCTGCCAA---CTCTACCCGAGGGAACGGAGGATAAAGATCA (x3) ← 3nt deletion; inframe

#118
TTGGGCTGTCTGTGGCCTGGTGC-----------TCTACCCGAGGGAACGGAGGATAAAGATCA (x9) ← 11nt deletion; out of frame
TTGGGCTGTCTGTGGCCTGGTGCCTGCCAAAG-CTCTACCCGAGGGAACGGAGGATAAAGATCA (x4) ← 1nt deletion; out of frame

#122
TTGGGCTGTCTGTGGCCTGGTGCCTGCCAAAGCCTCTACCCGAGGGAACGGAGGATAAAGATCA (x1) ← Wild type
---------------------------------------------GAACGGAGGATAAAGATCA (x5) ← 54nt deletion; inframe
```

Supplementary Figure 5. Two dimensional representation of the RHD protein structure

A two dimensional structure of the RHD protein translated from transcript variant 1 is shown. The positions of premature termination codons (PTCs) observed in E1_B and E4_B are indicated with arrows. The picture was re-drawn based on a previous presentation[47] (http://www.uniulm.de/~wflegel/RH/SympDGTI2004/4WagnerDGTI2004MA).

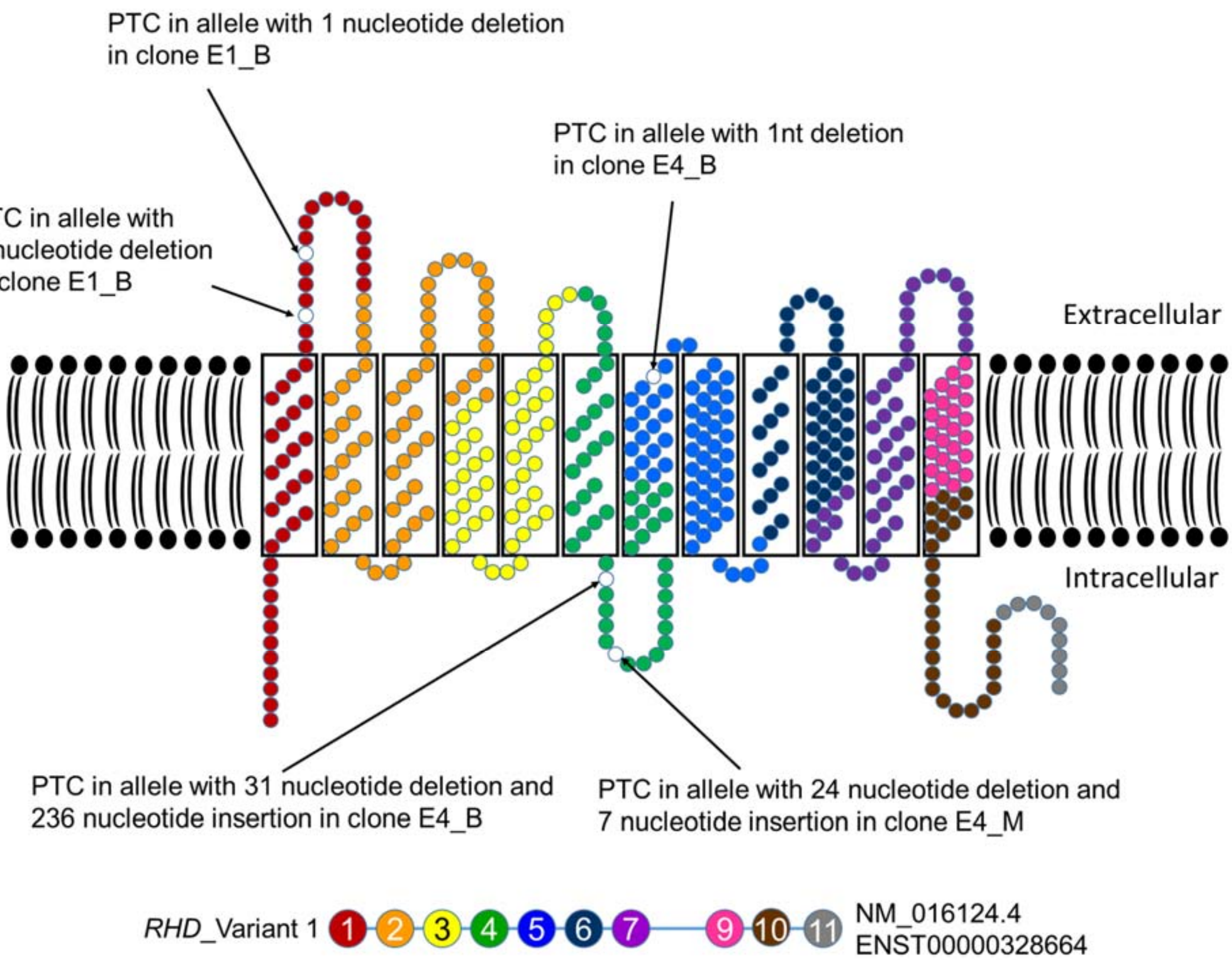

Supplementary Figure 6. Off-target mutations in the *RHD* mutant clones.

(a) Two potential off-target sites for *RHD*_E1_TALENs (left) and results from the T7E1 assay analyzing these two sites in the parental and the biallelic *RHD* mutant E1_B clone (right). Arrows indicate the expected positions of DNA bands cleaved by T7E1. The sizes of marker (M) bands are shown on the left (kbp, kilobase pairs). (b) DNA sequences at the off-target site (*RHCE)* in the clone E1_B. TALE binding sites are in a red font and spacer regions are indicated with green boxes. Deleted bases are indicated by dashes and inserted bases are shown in a blue font. The number of occurrences is shown in parentheses (e.g., x6 and x4 indicate the number of each sequence). The sequence and sequencing chromatogram for each allele are shown. nt, nucleotide. (c) Three potential off-target sites for *RHD*_E1_TALENs (left) and results from the T7E1 assay analyzing these three sites in the parental, E4_B, and E4_M clones (right). Arrows indicate the expected positions of DNA bands cleaved by T7E1. (d) DNA sequences of the off-target site (*RHCE)* in the clones E4_B and E4_M. Please refer to the legend to (b) for further details.

a

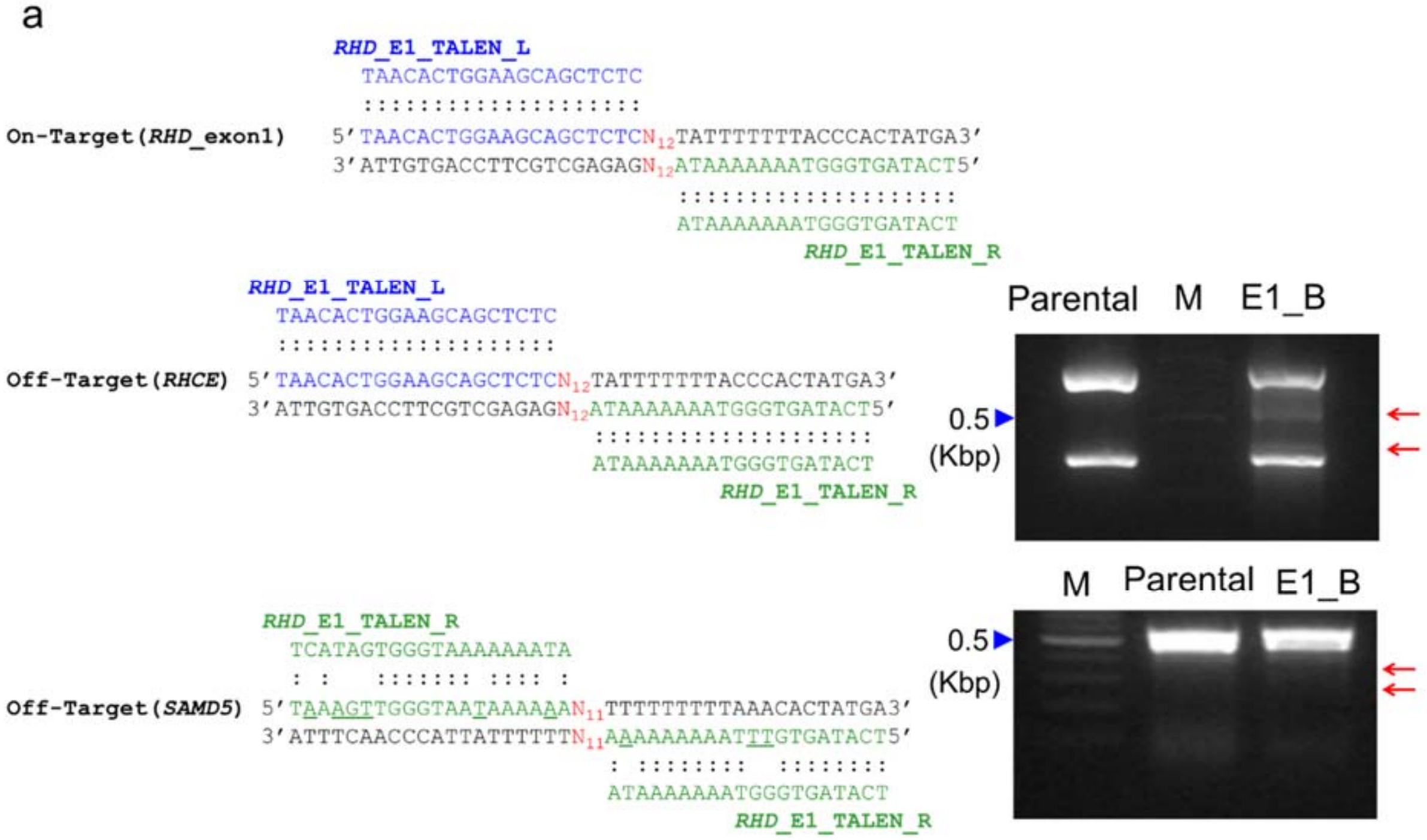

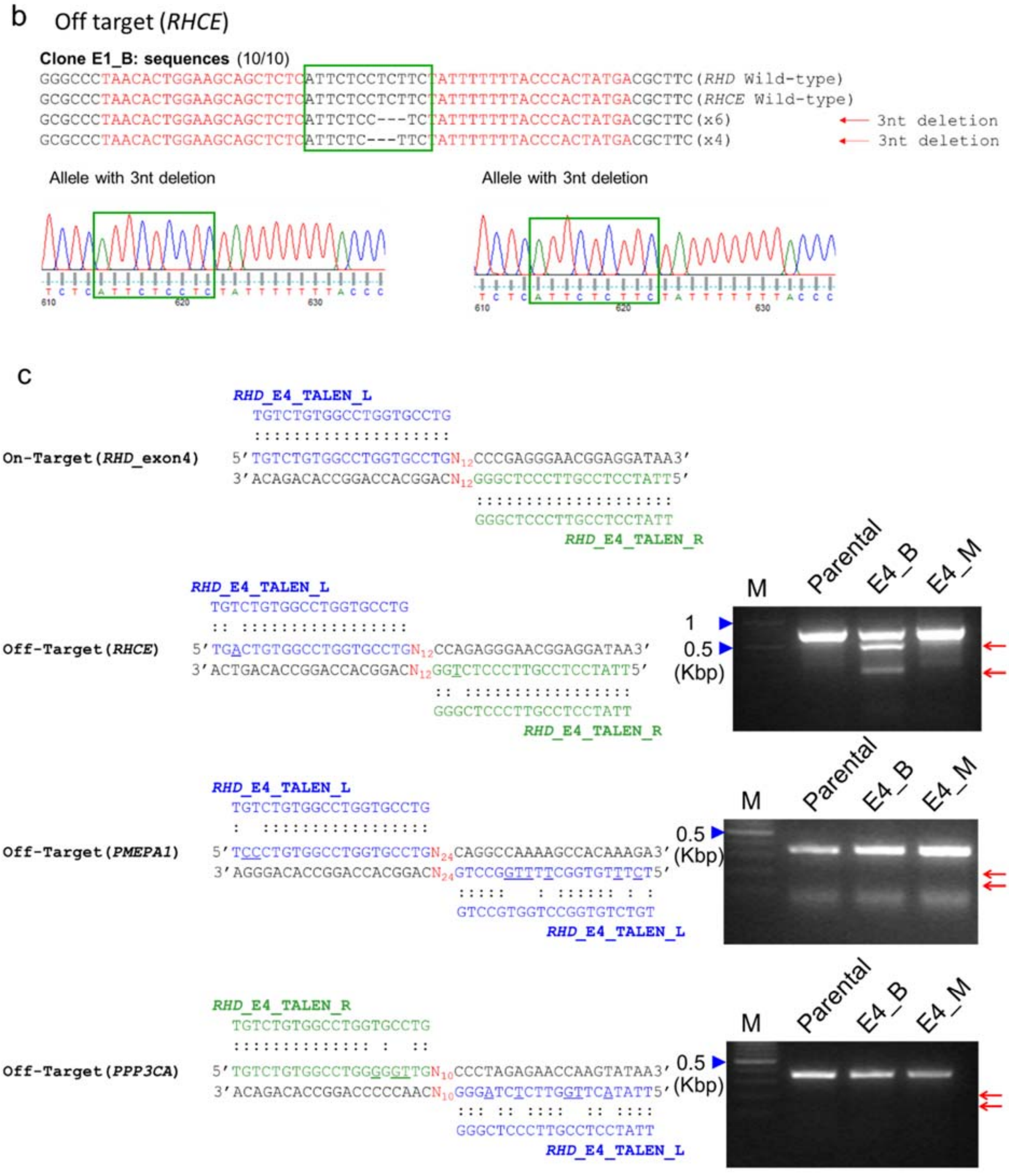
b Off target (RHCE)
Clone E1_B: sequences (10/10)
GGGCCCTAACACTGGAAGCAGCTCTCATTCTCCTCTTCTATTTTTTTACCCACTATGACGCTTC (RHD Wild-type)
GCGCCCTAACACTGGAAGCAGCTCTCATTCTCCTCTTCTATTTTTTTACCCACTATGACGCTTC (RHCE Wild-type)
GCGCCCTAACACTGGAAGCAGCTCTCATTCTCC---TCTATTTTTTTACCCACTATGACGCTTC (x6)
GCGCCCTAACACTGGAAGCAGCTCTCATTCTC---TTCTATTTTTTTACCCACTATGACGCTTC (x4)
3nt deletion
3nt deletion
Allele with 3nt deletion
Allele with 3nt deletion
610
620
630
c
RHD_E4_TALEN_L
TGTCTGTGGCCTGGTGCCTG
On-Target(RHD_exon4)
5’TGTCTGTGGCCTGGTGCCTGN12CCCGAGGGAACGGAGGATAA3’
3’ACAGACACCGGACCACGGACN12GGGCTCCCTTGCCTCCTATT5’
GGGCTCCCTTGCCTCCTATT
RHD_E4_TALEN_R
RHD_E4_TALEN_L
TGTCTGTGGCCTGGTGCCTG
Off-Target(RHCE)
5’TGACTGTGGCCTGGTGCCTGN12CCAGAGGGAACGGAGGATAA3’
3’ACTGACACCGGACCACGGACN12GGTCTCCCTTGCCTCCTATT5’
GGGCTCCCTTGCCTCCTATT
RHD_E4_TALEN_R
M
Parental
E4_B
E4_M
1
0.5
(Kbp)
RHD_E4_TALEN_L
TGTCTGTGGCCTGGTGCCTG
Off-Target(PMEPA1)
5’TCCCTGTGGCCTGGTGCCTGN24CAGGCCAAAAGCCACAAAGA3’
3’AGGGACACCGGACCACGGACN24GTCCGGTTTTCGGTGTTTCT5’
GTCCGTGGTCCGGTGTCTGT
RHD_E4_TALEN_L
M
Parental
E4_B
E4_M
0.5
(Kbp)
RHD_E4_TALEN_R
TGTCTGTGGCCTGGTGCCTG
Off-Target(PPP3CA)
5’TGTCTGTGGCCTGGGGGTTGN10CCCTAGAGAACCAAGTATAA3’
3’ACAGACACCGGACCCCCAACN10GGGATCTCTTGGTTCATATT5’
GGGCTCCCTTGCCTCCTATT
RHD_E4_TALEN_L
M
Parental
E4_B
E4_M
0.5
(Kbp)

d Off target (*RHCE*)

Clone E4_B: sequences (10/10)

```
TTGGGCTGTCTGTGGCCTGGTGCCTGCCAAAGCCTCTACCCGAGGGAACGGAGGATAAAGATCA(RHD Wild-type)
TTGGGCTGACTGTGGCCTGGTGCCTGCCAAAGCCTCTACCCAAGGGAACGGAGGATAATGATCA(RHCE Wild-type)
TTGGGCTGACTGTGGCCTGGTGCCTGCCA----------------GAACGGAGGATAATGATCA(x5)   <-- 16nt deletion
TTGGGCTGACTGTGGCCTGGTGCCTGCCAAAGCCTCTACCCAAGGGAACGGAGGATAATGATCA(x5)
```

Allele with 3nt deletion

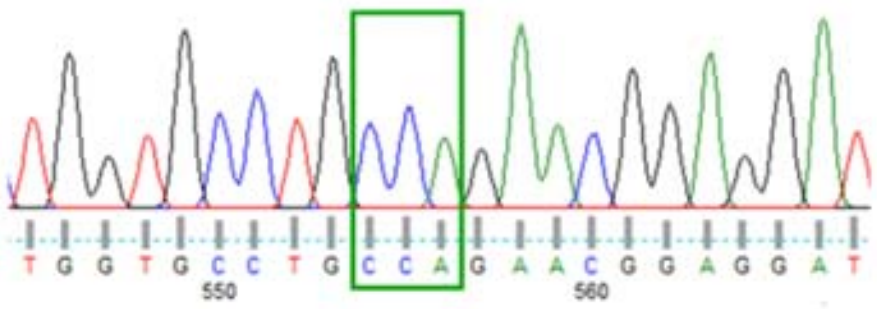

Allele with Wild type sequence

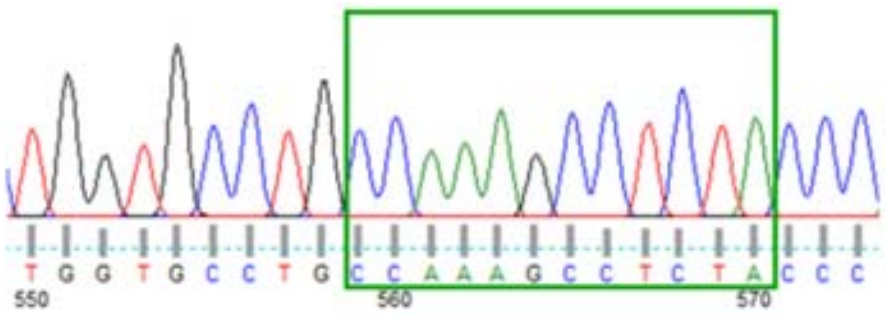

Clone E4_M: sequences (10/10)

```
TTGGGCTGTCTGTGGCCTGGTGCCTGCCAAAGCCTCTACCCGAGGGAACGGAGGATAAAGATCA(RHD Wild-type)
TTGGGCTGACTGTGGCCTGGTGCCTGCCAAAGCCTCTACCCAAGGGAACGGAGGATAATGATCA(RHCE Wild-type)
TTGGGCTGACTGTGGCCTGGTGCCTGCCAAAGCCTCTACCCAAGGGAACGGAGGATAATGATCA(x10)
```

Allele with Wild type sequence

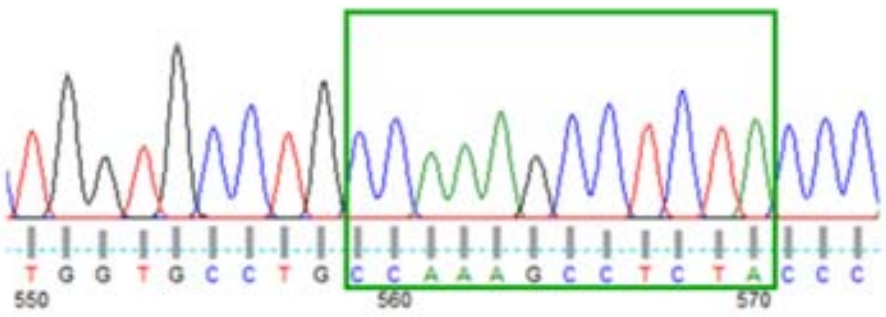

Allele with Wild type sequence

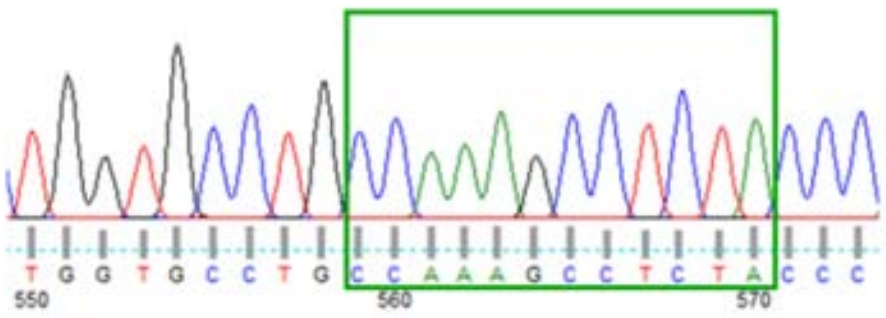

Supplementary Figure 7. Schematic representation of *RHD* mRNA variants in erythroid cells derived from cord blood CD34⁺ cells and HUDEP-2 cells.

RT-PCR was performed to detect *RHD* mRNA in each cell type; the amplicons were then subjected to sequencing. The number of occurrences is shown on the right of each transcript. The blue circles indicate exons.

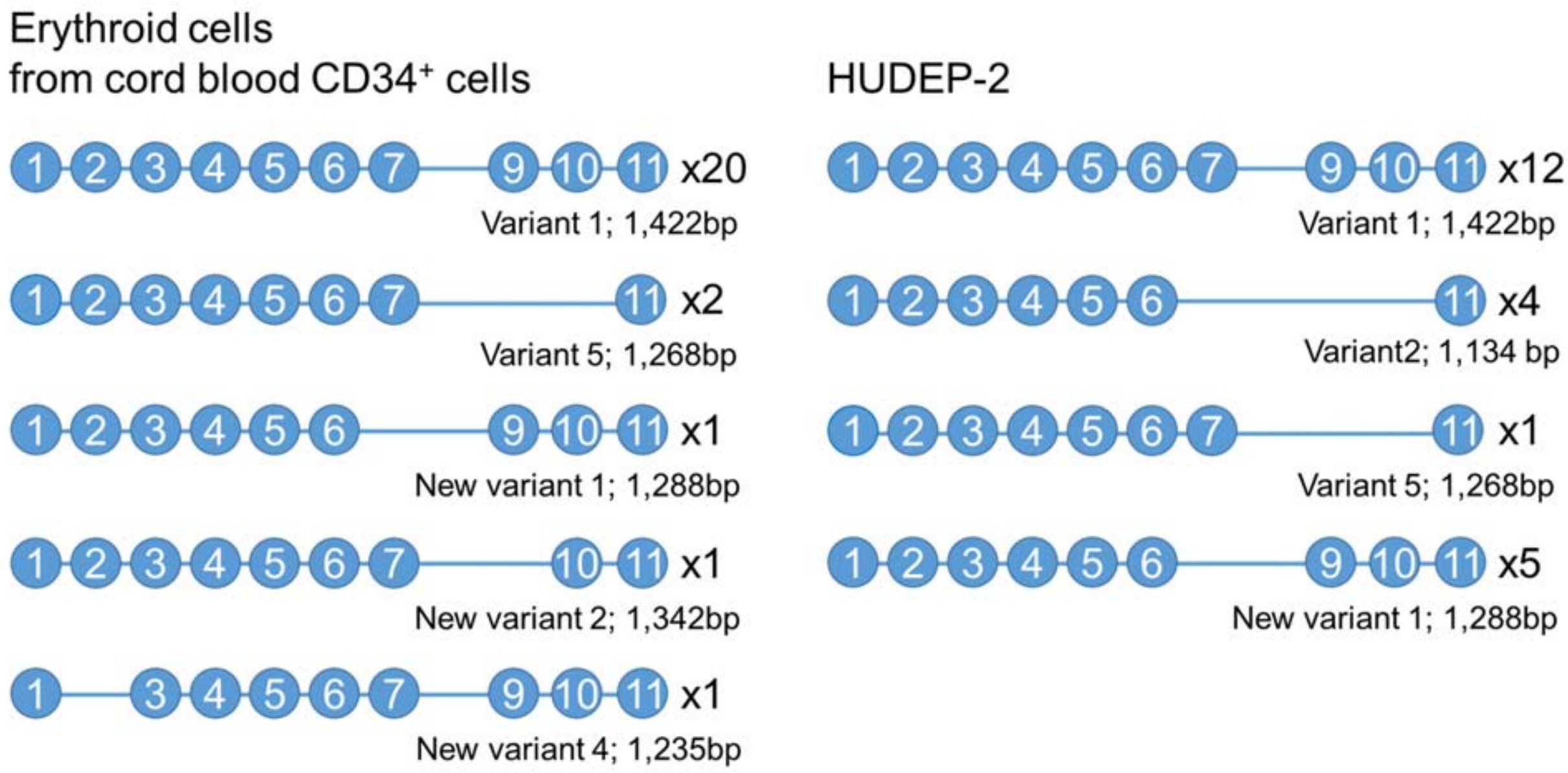

Supplementary Figure 8. Flow cytometric analysis of D antigen expression in mutated cells

Parental and *RHD*-mutated (biallelic, E1_B, E4_B; monoallelic, E4_M) HiDEP-1 cells were induced for differentiation for four days and subjected to flow cytometry. Dot plots show that D antigen is expressed in parental and monoallelic mutated cells, but not in biallelic mutated cells. Rh D-positive and D-negative peripheral blood cells were used as positive and negative controls, respectively.

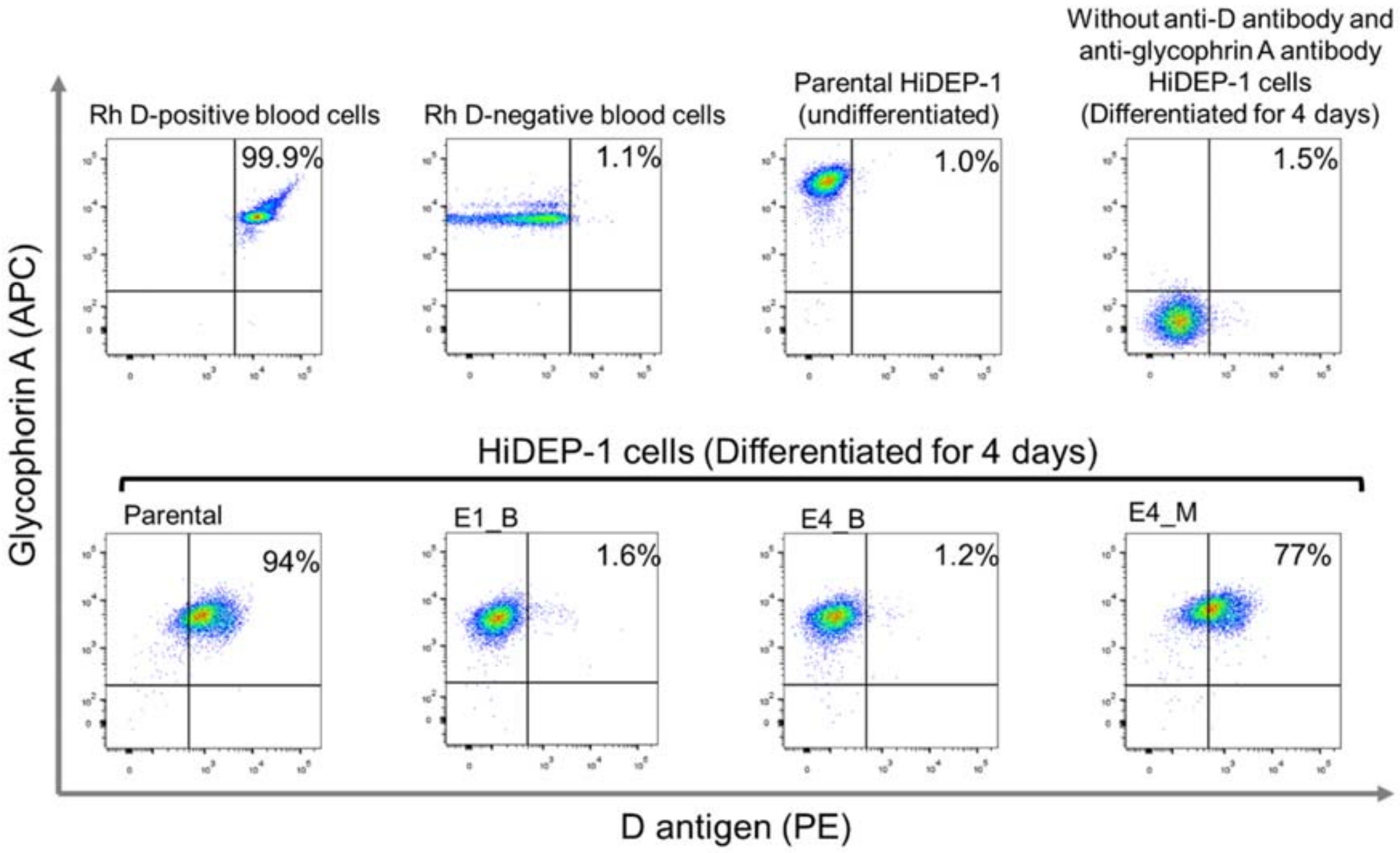

Supplementary Figure 9. Absence of Rh D antigen-mediated agglutination in *RHD*-knockout cell lines

Parental, *RHD*-knockout (E1_B, E4_B), and *RHD*-monoallelic mutant (E4_M) HiDEP-1 cells were induced for differentiation for four days and subjected to an agglutination test using anti-D blood grouping reagents in 96 well plates. Rh D-positive and D-negative blood cells were used as positive and negative controls, respectively. Pictures of the agglutination test covering whole test wells are shown.

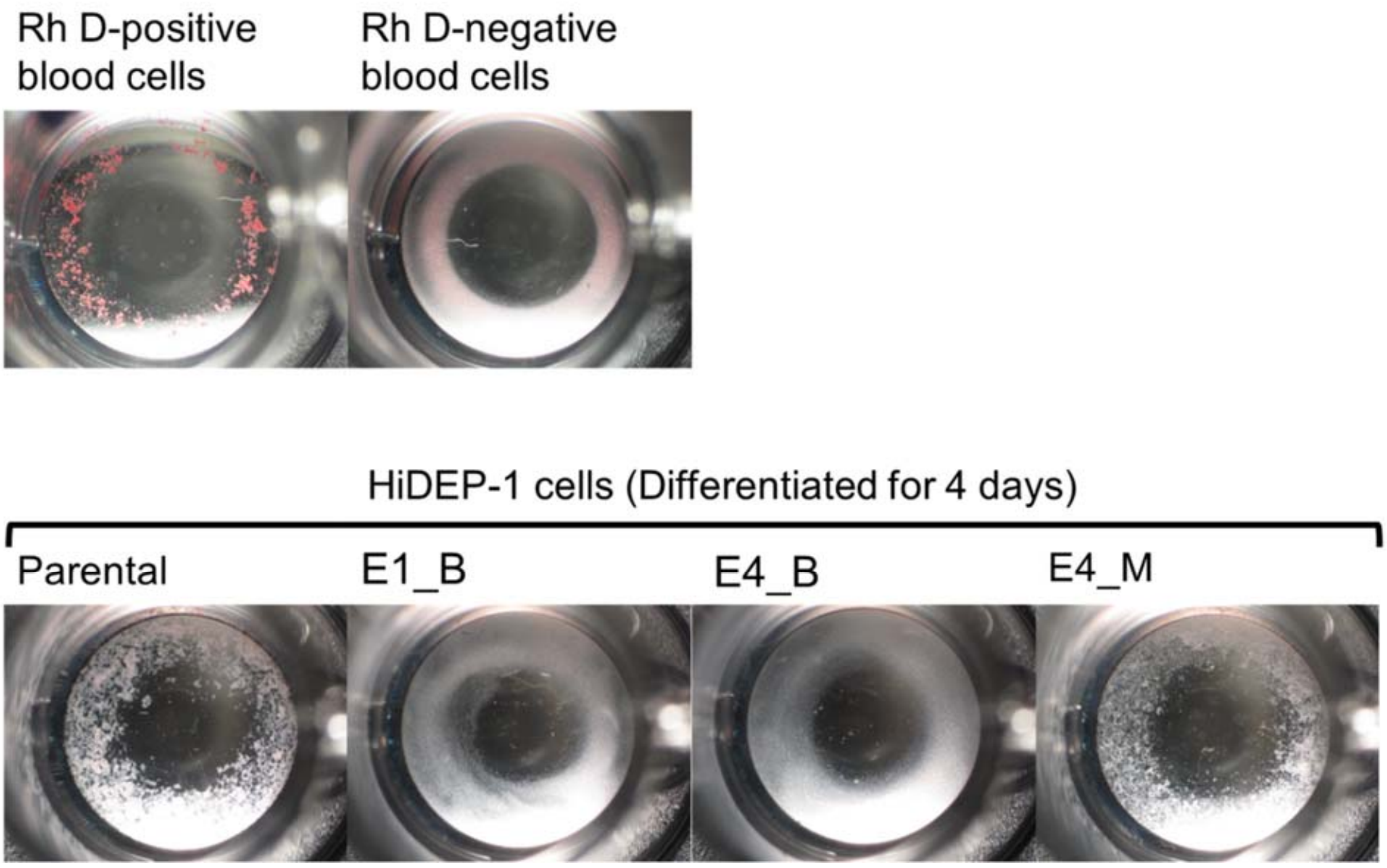

Supplementary Figure 10. Expression of erythroblast specific markers in parental and mutant cells.

Parental and *RHD*-mutated (biallelic, E1_B, E4_B; monoallelic, E4_M) HiDEP-1 cells were induced for differentiation for four days and subjected to flow cytometry to evaluate glycophorin A and CD71 expression. (a) Representative flow cytometry. (b) The expression levels of glycophorin A and CD71. The percentage of glycophorin A^{+}CD71^{+} cells was the same in the parental and *RHD*-mutant clones (n = 4, ANOVA was performed). Error bars represent the standard error of the mean (S.E.M.).

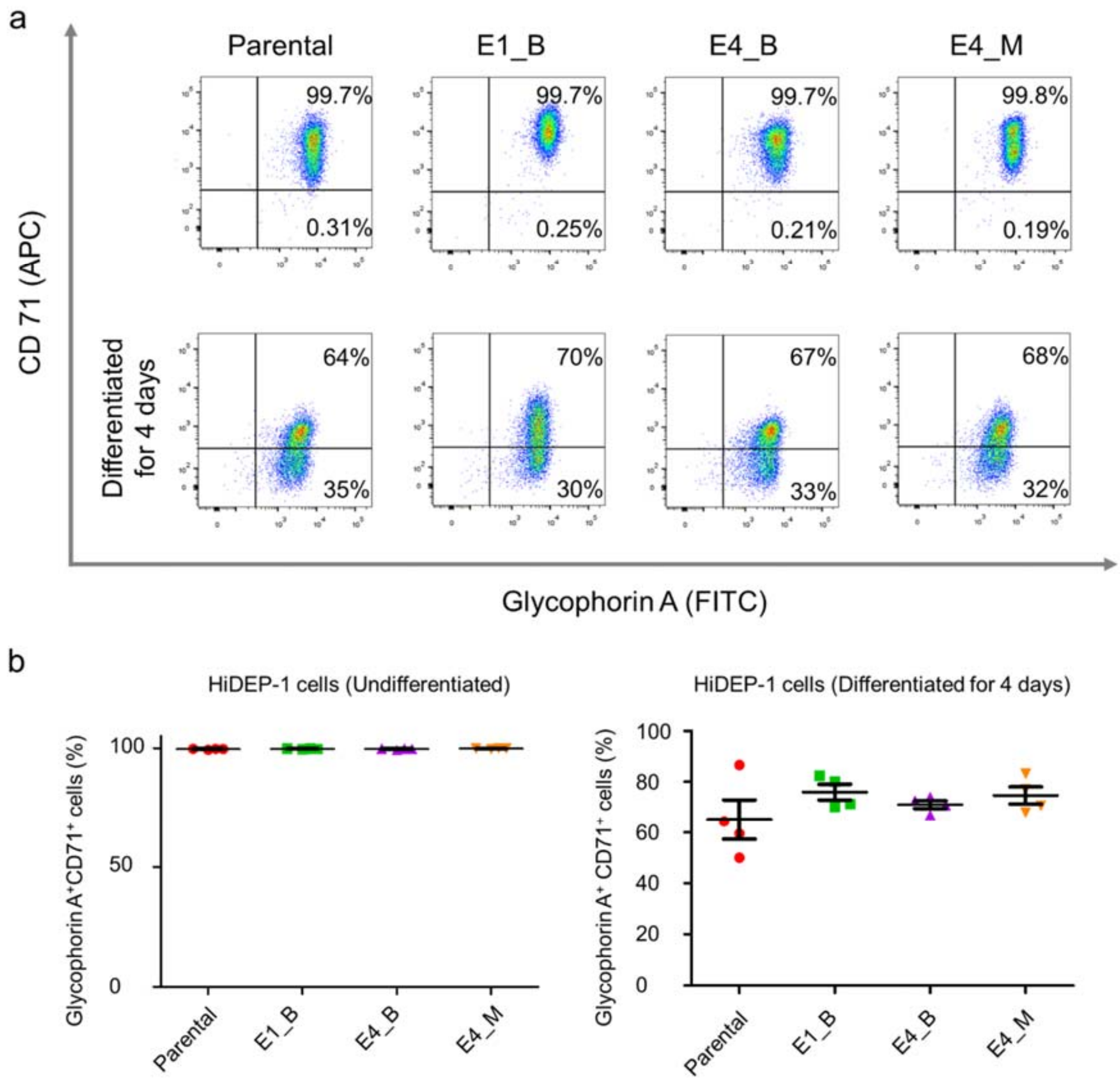

Supplementary Figure 11. Uncropped images of gels shown in Fig. 1b (a) and 1c (b).

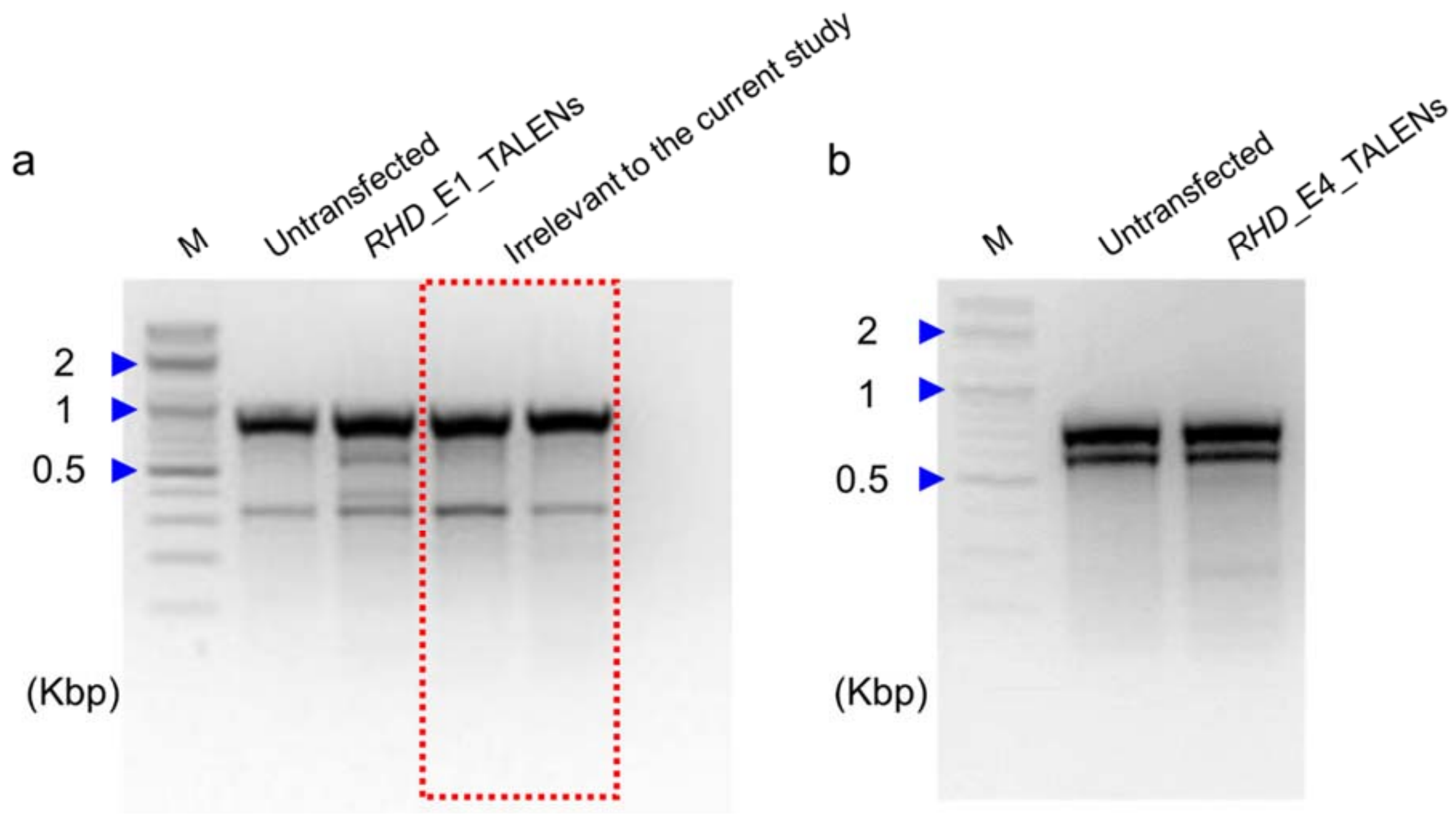

Supplementary Figure 12. Uncropped images of gels shown in Fig. 2b.

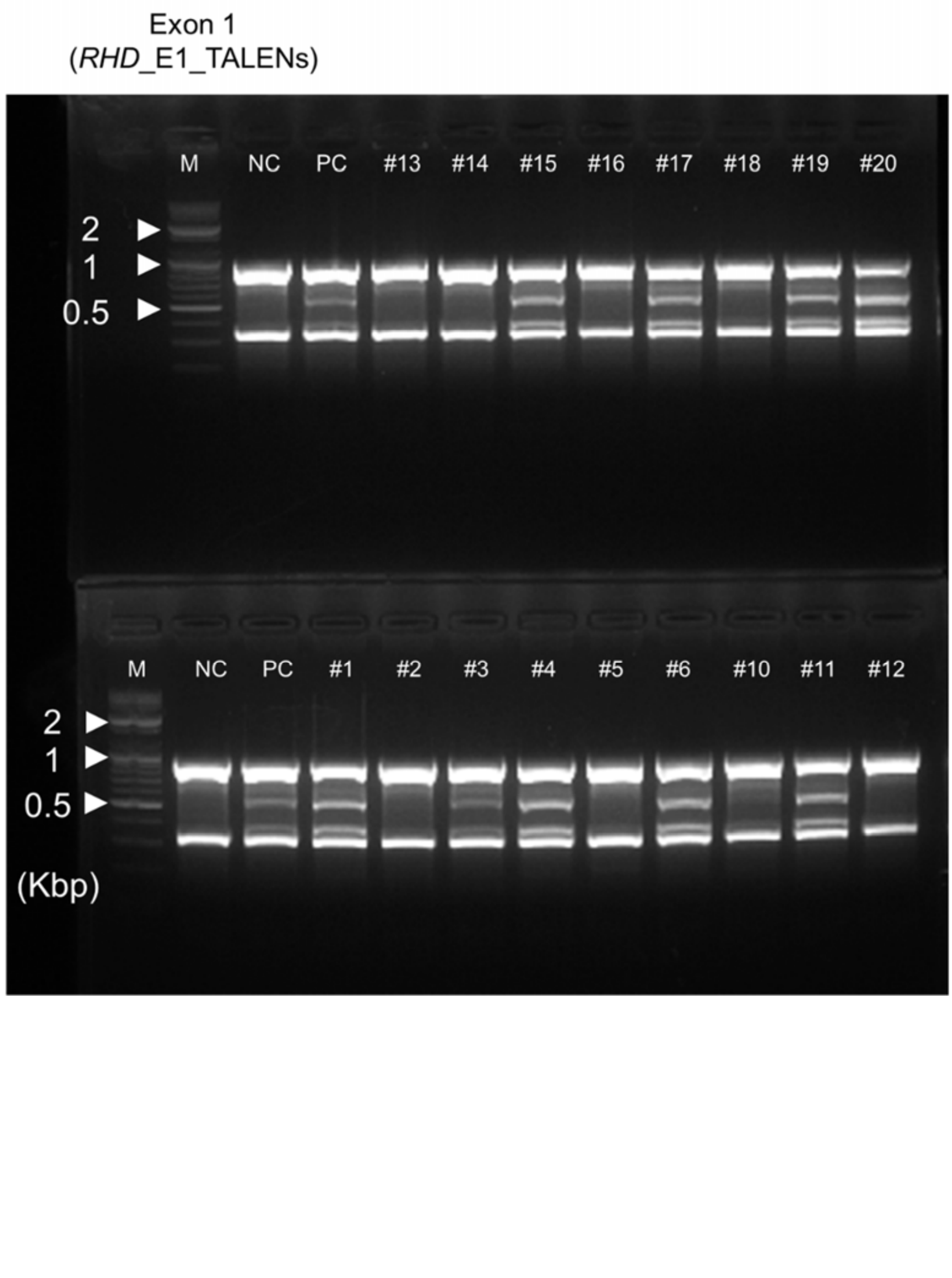

Exon 4
(*RHD*_E4_TALENs)

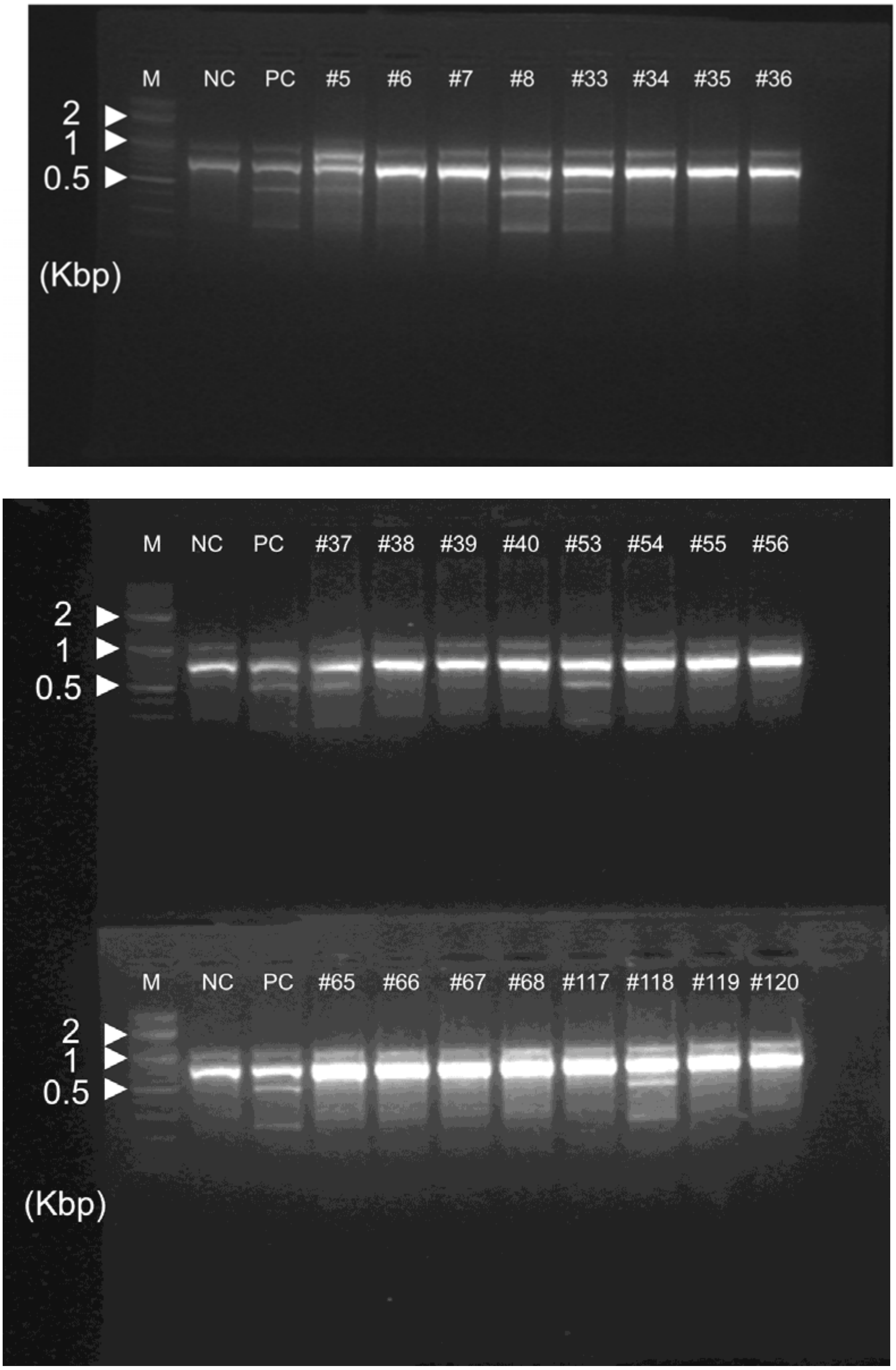

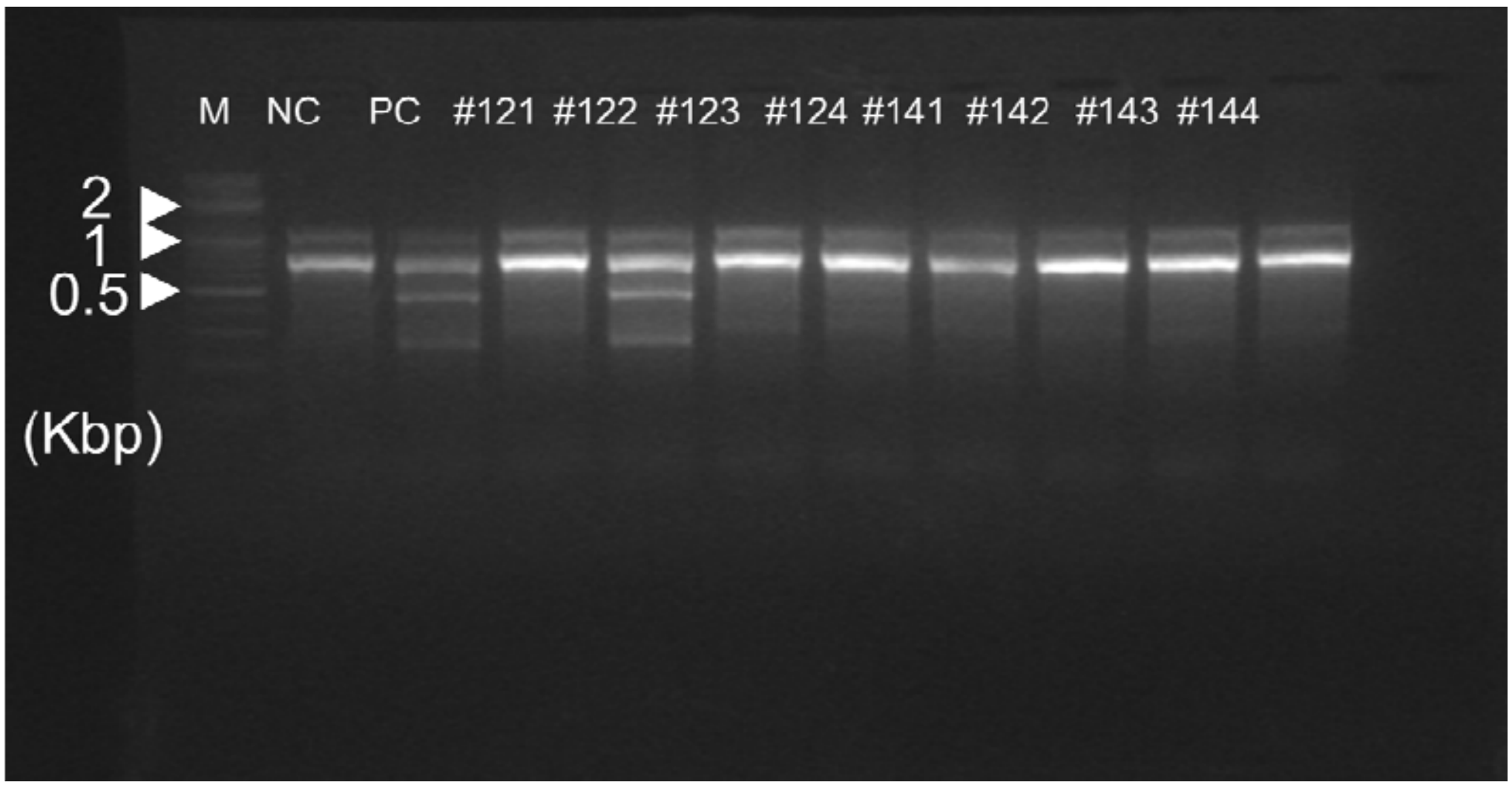
M NC PC #121 #122 #123 #124 #141 #142 #143 #144
2
1
0.5
(Kbp)

Supplementary Figure 13. Uncropped images of gels shown in Supplementary Fig 3.

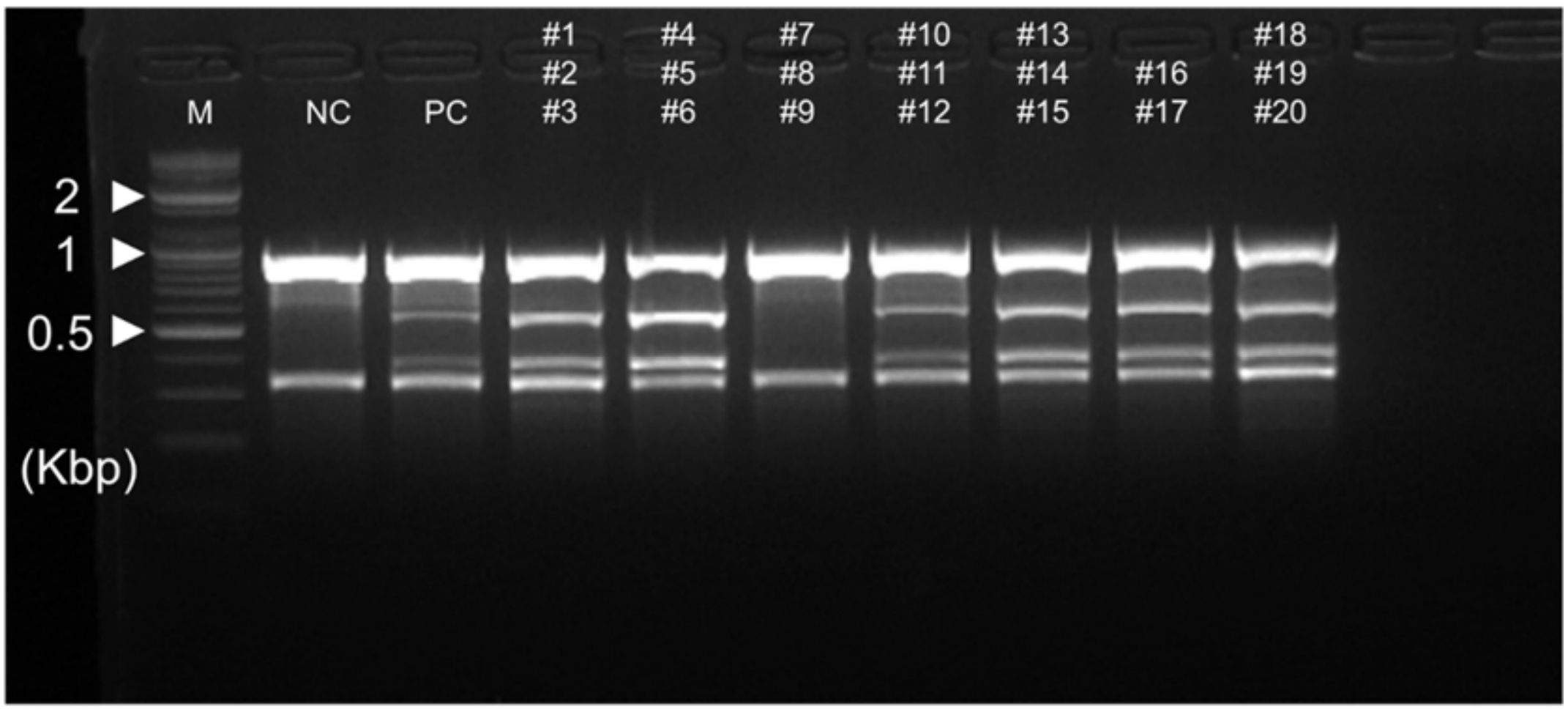

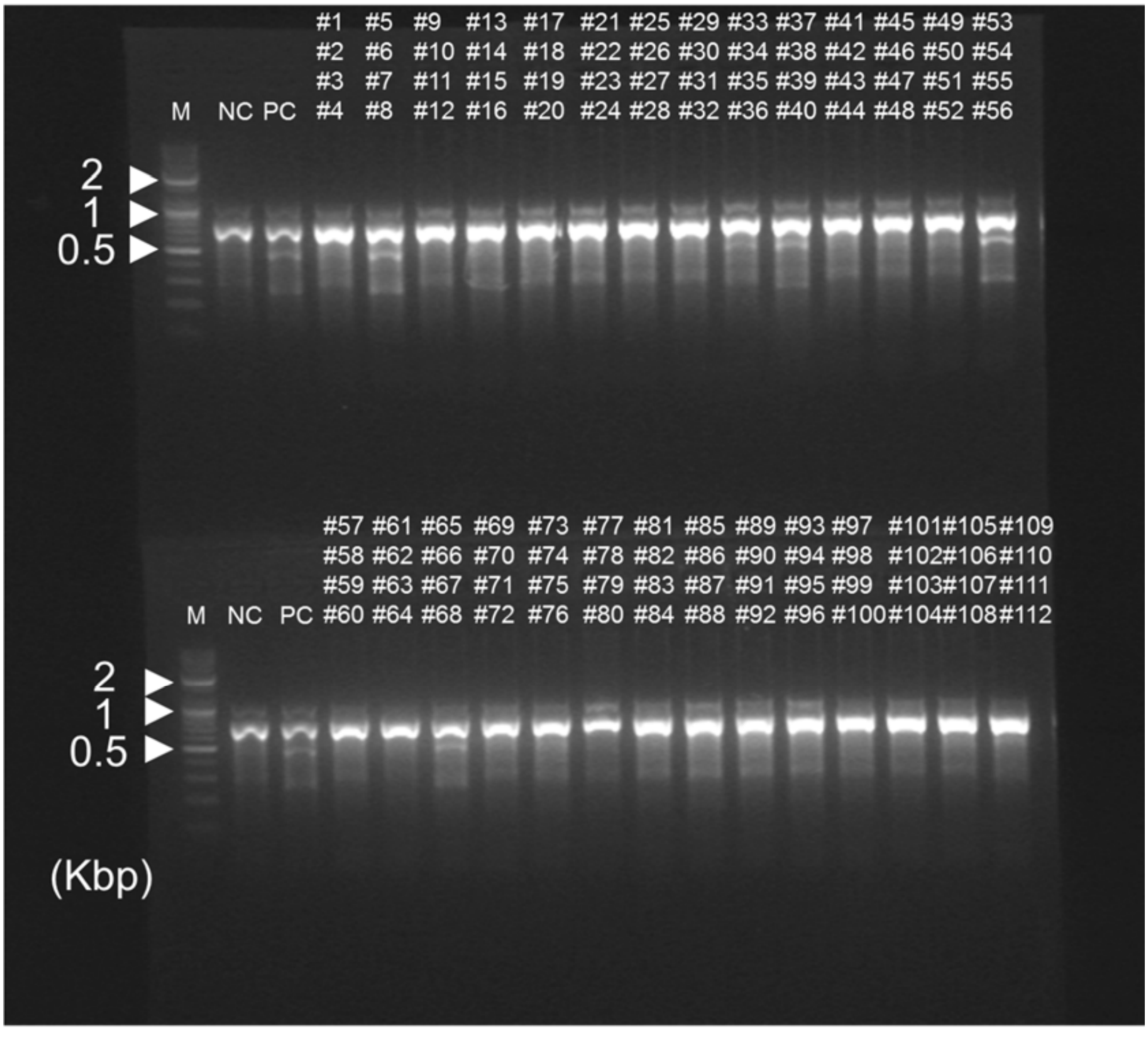

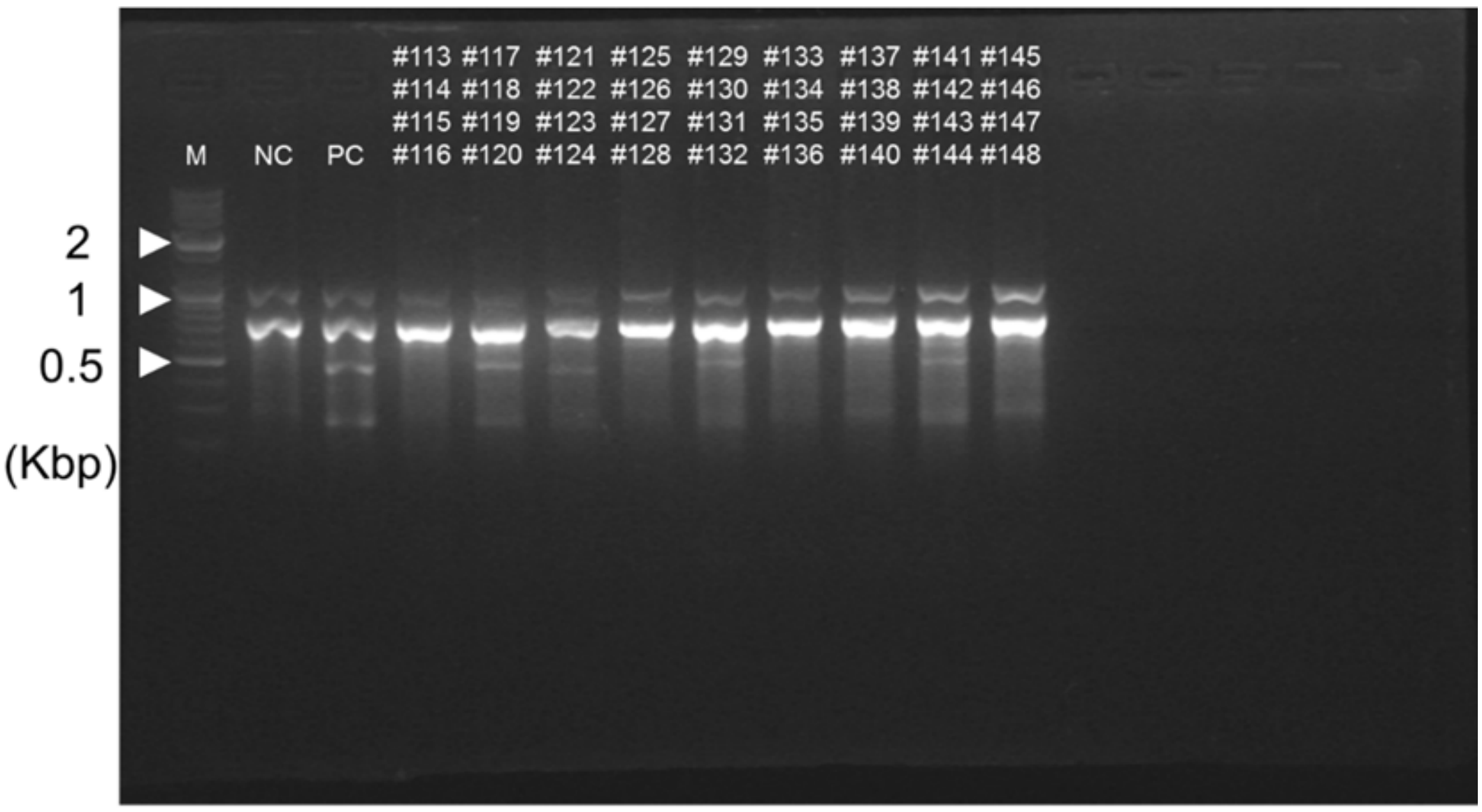
2
1
0.5
(Kbp)
M
NC
PC
#113 #114 #115 #116
#117 #118 #119 #120
#121 #122 #123 #124
#125 #126 #127 #128
#129 #130 #131 #132
#133 #134 #135 #136
#137 #138 #139 #140
#141 #142 #143 #144
#145 #146 #147 #148

Supplementary Figure 14. Uncropped images of gels shown in Supplementary Fig. 6a (a) and 6c (b).

a

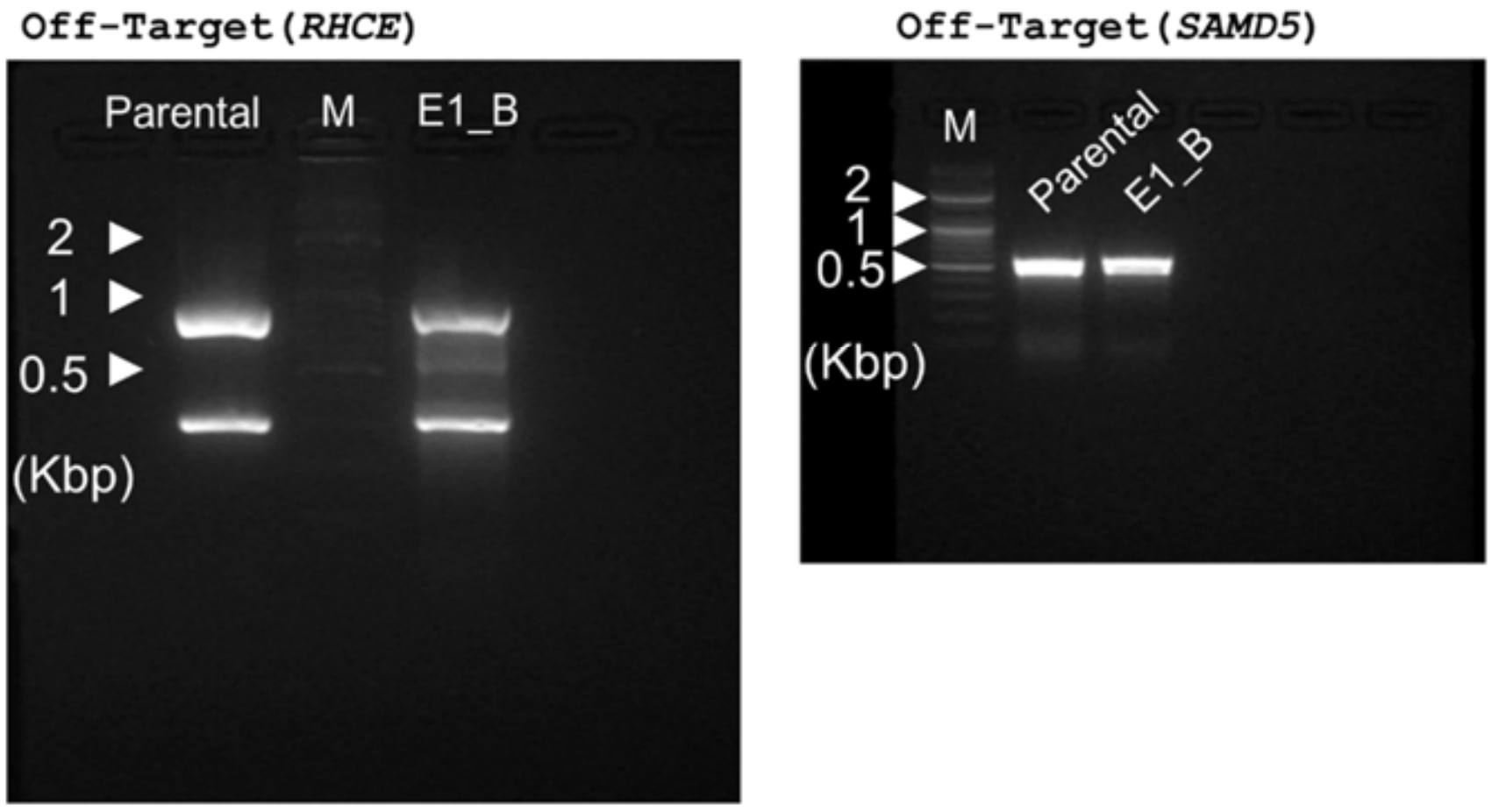

b

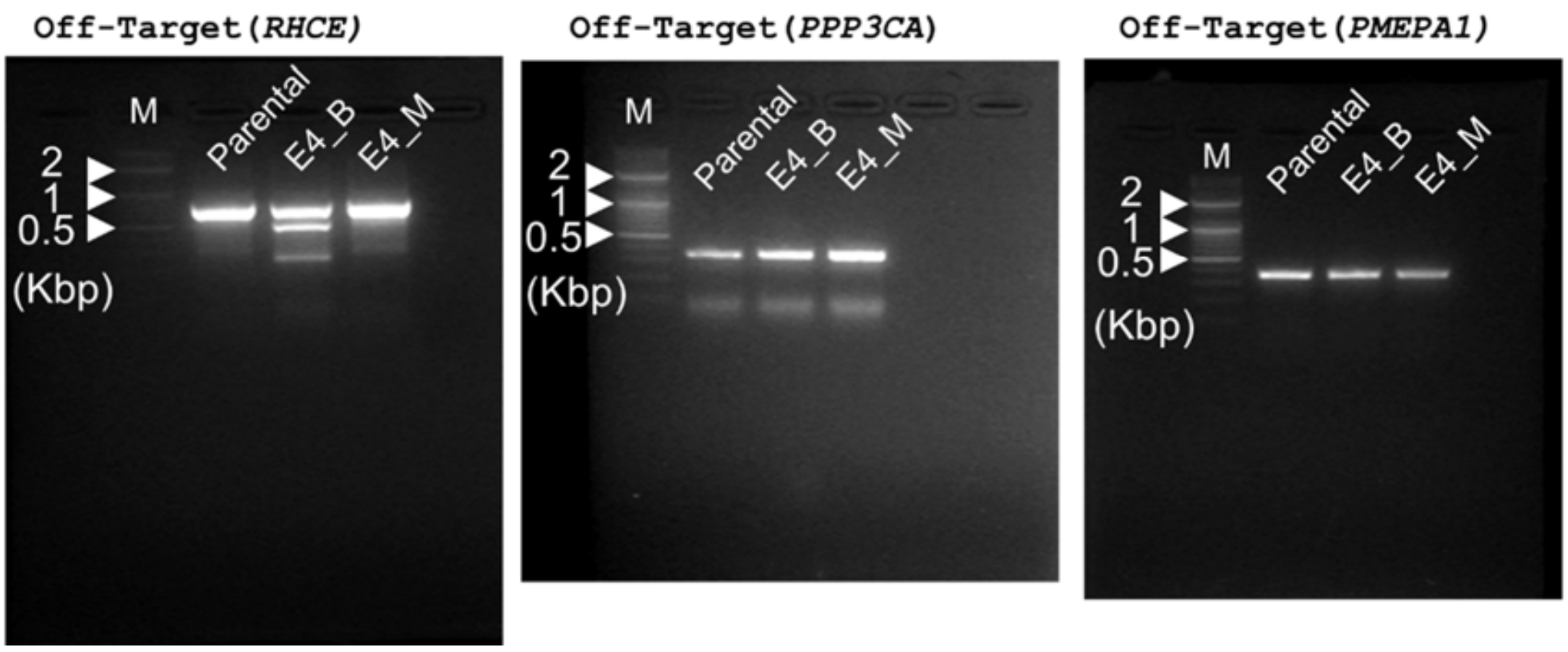

Supplementary Figure 15. Uncropped images of gels shown in Fig. 4a.

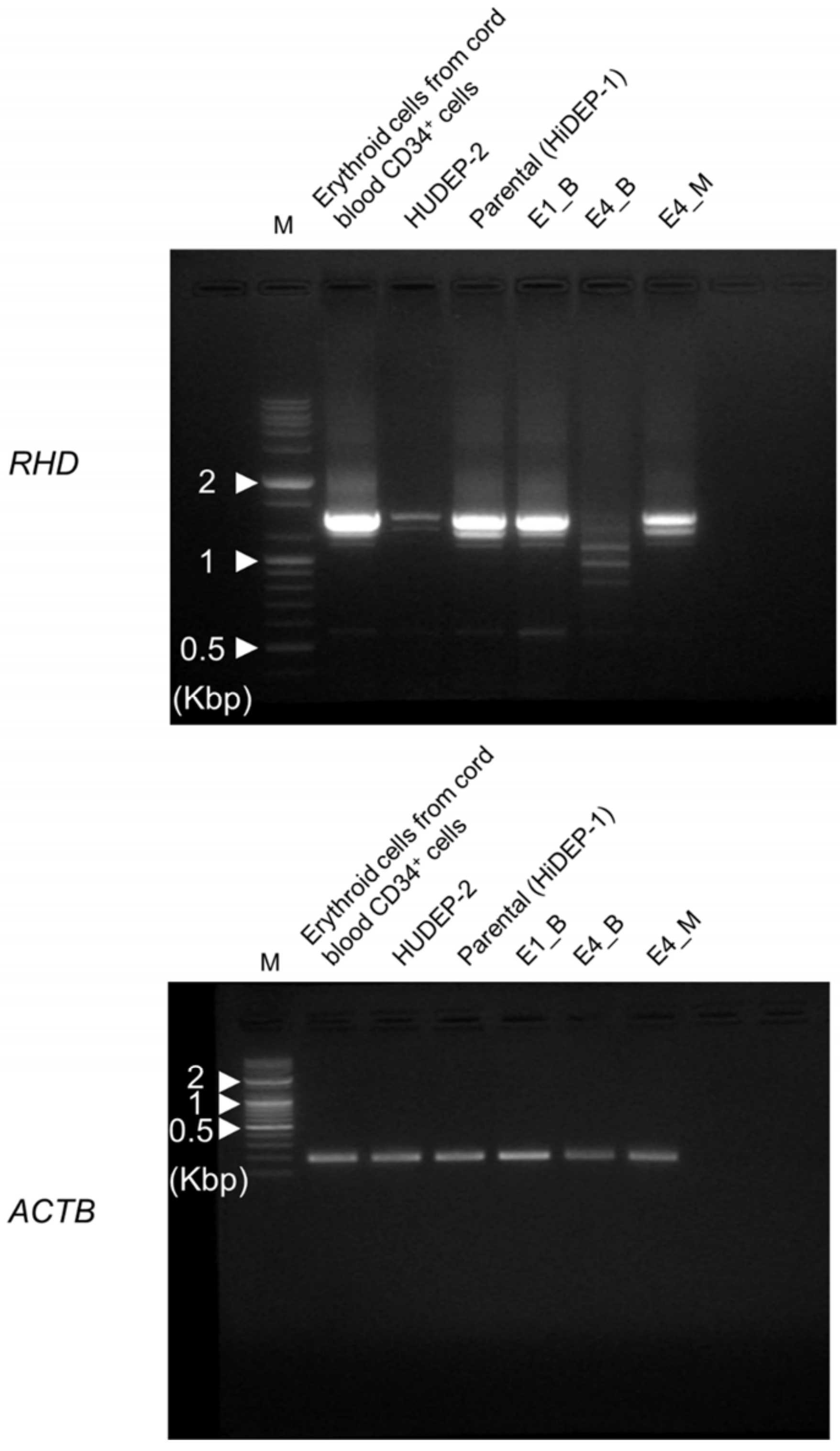

Supplementary Table 1. The sequences of primers used in this study.

primer	sequence (5'-3')
*RHD*_exon1_1st_forward	ATG GGA GCA CAG GGG AAG TT
*RHD*_exon1_1st_reverse	AAA AAT TAG CCA GGC ATG GT
*RHD*_exon1_2nd_forward	TTA AGA GCT CAC TGG GTG CC
*RHD*_exon1_2nd_reverse	GGG GAA GCA GAG AAG CAG AG
*RHD*_exon4_1st_forward	GGT CAA AAG CAT ATA AGA GCT ACT G
*RHD*_exon4_1st_reverse	ACT CCC CGT TAA GCA CTT TAC AT
*RHD*_exon4_2nd_forward	GCA GTG GCT CAT GCC TGT AAT
*RHD*_exon4_2nd_reverse	CCT GCT CTG TGA AGT GCT TAA TTC
sequencing_T7 primer	TAA TAC GAC TCA CTA TAG GG
RT-PCR_*RHD*_forward	ACA CAG GAT GAG CTC TAA GT
RT-PCR_*RHD*_reverse	GTG GCA GAG AAA GGA TTC AAC TCC
off-target_*RHCE*_exon1_1st_forward	GGA TGG GAG CAC AGT TCC GG
off-target_*RHCE*_exon1_1st_reverse	AAT TTA GCC AGG CAT GGT GGT G
off-target_*RHCE*_exon1_2nd_forward	TTA AGA GCT CAC TGG GTG CC
off-target_*RHCE*_exon1_2nd_reverse	GGG GAA GCA GAG AAG CAG AG
off-target_*RHCE*_exon4_1st_forward	CTT TCT CCA GTA GCC CAC TAA A
off-target_*RHCE*_exon4_1st_reverse	TCA CTT GTC CGT TTC CCT CC
off-target_*RHCE*_exon4_2nd_forward	CAC AGT GGC TCA TGC CTG TA
off-target_*RHCE*_exon4_2nd_reverse	GGT CCC TAA AAG GAA GTG CTT
off-target_*SAMD5*_forward	CAA TGA GTT CCA GGC TAC CAG AAG
off-target_*SAMD5*_reverse	GCC ATT TAT TGA GGT TTT CCC CCC
off-target_*PMEPA1*_forward	GGG CTT TTT CTC TGA TGA GTG CTG
off-target_*PMEPA1*_reverse	AAA AGA ACA CTC CAA GTA CCC CCG
off-target_*PPP3CA*_forward	CTT TTG ATA TCC CTG GGG GAT TGG
off-target_*PPP3CA*_reverse	GTG CTT GCA CTC CTA TGA GAG TC

Supplementary Note 1. Map and sequences of the TALEN-encoding plasmids used in this study.

***RHD*_Exon1_TALEN_L**

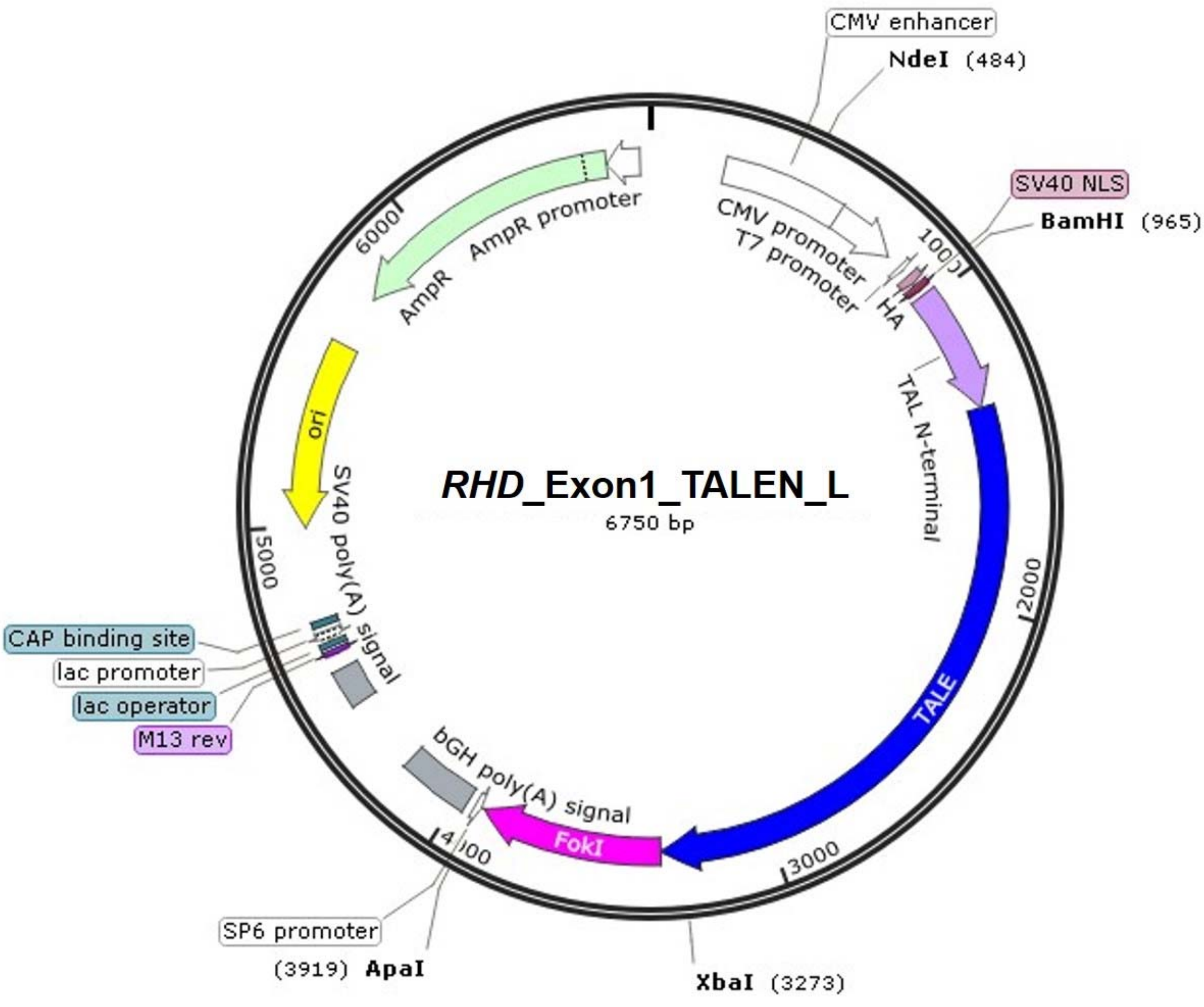

CMV promoter
T7 Promoter
Star codon-HA tag-NLS
TAL N-terminal domain
Target Specific TALE array
Fok I (DAS(Left)/RR(Right) – heterodimer-specific FokI variant)

gacggatcgggagatctcccgatcccctatggtcgactctcagtacaatctgctctgatgccgcatagttaagccag
tatctgctccctgcttgtgtgttggaggtcgctgagtagtgcgcgagcaaaatttaagctacaacaaggcaaggctt
gaccgacaattgcatgaagaatctgcttagggttaggcgttttgcgctgcttcgcgatgtacgggccagatatacgc
gttgacattgattattgactagttattaatagtaatcaattacggggtcattagttcatagcccatatatggagttc
cgcgttacataacttacggtaaatggcccgcctggctgaccgcccaacgacccccgcccattgacgtcaataatgac
gtatgttcccatagtaacgccaatagggactttccattgacgtcaatgggtggactatttacggtaaactgcccact
tggcagtacatcaagtgtatcatatgccaagtacgccccctattgacgtcaatgacggtaaatggcccgcctggcat
tatgcccagtacatgaccttatgggactttcctacttggcagtacatctacgtattagtcatcgctattaccatggt
gatgcggttttggcagtacatcaatgggcgtggatagcggtttgactcacggggatttccaagtctccaccccattg
acgtcaatgggagtttgttttggcaccaaaatcaacgggactttccaaaatgtcgtaacaactccgccccattgacg
caaatgggcggtaggcgtgtacggtgggaggtctatataagcagagctctctggctaactagagaacccactgctta
ctggcttatcgaaattaatacgactcactatagggagatgcaagcttgccaccatggtgtacccctacgacgtgccc
gactacgccgaattgcctccaaaaaagaagagaaaggtagggatccgaattcaagatctacgcacgctcggctacag
ccagcagcaacaggagaagatcaaaccgaaggttcgttcgacagtggcgcagcaccacgaggcactggtcggccatg
ggtttacacacgcgcacatcgttgcgctcagccaacacccggcagcgttagggaccgtcgctgtcaagtatcaggac
atgatcgcagcgttgccagaggcgacacacgaagcgatcgttggcgtcggcaaacagtggtccggcgcacgcgctct
ggaggccttgctcacggtggcgggagagttgagaggtccaccgttacagttggacacaggccaacttctcaagattg
caaaacgtggcggcgtgaccgcagtggaggcagtgcatgcatggcgcaatgcactgacgggtgcccccctgaACCTC
ACTCCAGATCAGGTGGTGGCTATCGCTAGTAATATTGGAGGAAAGCAAGCCCTCGAAACCGTTCAACGGCTGTTGCC
AGTGCTCTGTCAGGCCCATGGTCTTACCCCCGACCAGGTTGTGGCAATTGCTTCCAACATCGGCGGGAAGCAAGCAC
TCGAGACAGTGCAAAGACTTCTTCCCGTTCTCTGTCAAGCTCACGGATTGACCCCTGCACAAGTCGTCGCCATTGCC
AGCCATGACGGGGGTAAACAAGCCCTCGAGACTGTCCAACGCTTGCTCCCTGTGTTGTGCCAGGCACATGGgttgAC
CCCTGAGCAAGTGGTTGCCATTGCCAGTAATATCGGCGGCAAGCAGGCTTTGGAAACTGTTCAGAGATTGCTGCCCG
TTCTTTGCCAAGCACATGGCTTGACACCCGATCAAGTTGTTGCTATCGCTAGCCATGATGGAGGGAAACAAGCCCTT
GAGACTGTGCAACGGCTGCTTCCAGTGTTGTGCCAAGCTCATGGACTTACTCCCGATCAGGTCGTGGCTATTGCATC
AAATGGTGGTGGCAAACAAGCACTGGAAACCGTTCAAAGGTTGCTTCCTGTTCTGTGTCAGGACCACGGATTGACac
caGCACAGGTTGTCGCAATCGCATCCAACAATGGAGGAAAACAAGCACTCGAAACCGTTCAGCGCCTGCTTCCTGTC
TTGTGTCAGGCACACGGACTGACTCCAGACCAGGTGGTTGCTATCGCCAGCAATAACGGAGGTAAACAAGCCTTGGA
GACTGTCCAAAGGTTGTTGCCCGTTCTCTGCCAAGACCATGGTTTGACACCAGCTCAAGTGGTTGCCATCGCTTCTA
ATATCGGGGGTAAGCAGGCACTCGAGACAGTCCAGAGACTCCTGCCTGTTCTCTGCcaagCCCACGGCCTCACACCA
GCTCAGGTTGTTGCCATCGCTAGCAATATTGGTGGGAAACAGGCTCTTGAAACCGTTCAACGCCTCCTTCCTGTGCT
CTGTCAAGACCATGGTCTTACACCCGATCAAGTCGTGGCTATTGCTTCAAACAACGGAGGAAAGCAAGCACTTGAGA
CTGTCCAACGCCTTCTGCCAGTCCTCTGCCAGGATCATGGCCTGACTCCAGAACAAGTTGTGGCAATCGCATCTCAC
GATGGCGGGAAACAGGCATTGGAAACCGTTCAAAGGTTGCTGCCAGTCCTTTGTCAGGCCCACGGCCTgactCCAGC
TCAGGTTGTTGCCATCGCTAGCAATATTGGTGGGAAACAGGCTCTTGAAACCGTTCAACGCCTCCTTCCTGTGCTCT
GTCAAGACCATGGTCTTACACCCGATCAAGTCGTGGCTATTGCTTCAAACAACGGAGGAAAGCAAGCACTTGAGACT
GTCCAACGCCTTCTGCCAGTCCTCTGCCAGGATCATGGCCTGACTCCAGAACAAGTTGTGGCAATCGCATCTCACGA
TGGCGGGAAACAGGCATTGGAAACCGTTCAAAGGTTGCTGCCAGTCCTTTGTCAGGCtcatGGGCTGACCCCTGAGC
AGGTTGTCGCTATCGCTTCCAATGGCGGTGGCAAACAGGCATTGGAGACAGTCCAAAGACTCTTGCCCGTCTTGTGT
CAGGCACACGGGCTTACACCAGATCAGGTGGTCGCCATCGCCAGTCATGACGGCGGAAAACAGGCACTGGAGACTGT
GCAACGCTTGCTTCCTGTTCTTTGTCAAGATCACGGCTTGACTCCCGACCAGGTCGTGGCCATCGCCTCAAATGGGG
GAGGGAAGCAAGCACTTGAAACTGTTCAACGGCTTCTCCCAGTGCTGTGTCAGGCTCAcggtctgactccggaacag
gtggtggcgattgcaagccatgatggcggcaaacaggctctagagagcattgttgcccagctctccagacctgatcc
ggcgctagccgcgttgctagtcaaaagtgaaCtcgaggagaagaaatctgaacttcgtcataaattgaaatatgtgc
ctcatgaatatattgaattaattgaaattgccagaaatcccactcaggatagaattcttgaaatgaaggtaatggaa
tttttatgaaagtttatggatatagaggtgagcatttgggtggatcaaggaaaccggacggagcaatttatactgt
cggatctcctattgattacggtgtgatcgtggatactaaagcttatagcggaggttataatctgccaattggccaag
cagatgccatgcaaagctatgtcgaagaaaatcaaacacgaaacaaacatatcaaccctaatgaatggtggaaagtc
tatccatcttctgtaacggaatttaagttttatttgtgagtggtcactttaaaggaaactacaaagctcagcttac
acgattaaatcatatcactaattgtaatggagctgttcttagtgtagaagagcttttaattggtggagaaatgatta

aagccggcacattaaccttagaggaagtgagacggaaatttaataacggcgagataaacttttagggccctattcta
tagtgtcacctaaatgctagagctcgctgatcagcctcgactgtgccttctagttgccagccatctgttgtttgccc
ctcccccgtgccttccttgaccctggaaggtgccactcccactgtcctttcctaataaaatgaggaaattgcatcgc
attgtctgagtaggtgtcattctattctggggggtggggtggggcaggacagcaagggggaggattgggaagacaat
agcaggcatgctggggatgcggtgggctctatggcttctgaggcggaaagaaccagctggggctctagggggtatcc
ccacgctgcagtgagcgggactctggggttcgaaatgaccgaccaagcgacgcccaacctgccatcacgagatttcg
attccaccgccgccttctatgaaaggttgggcttcggaatcgttttccgggacgccggctggatgatcctccagcgc
ggggatctcatgctggagttcttcgcccaccccaacttgtttattgcagcttataatggttacaaataaagcaatag
catcacaaatttcacaaataaagcatttttttcactgcattctagttgtggtttgtccaaactcatcaatgtatctt
atcatgtctgtataccgtcgacctctagctagagcttggcgtaatcatggtcatagctgtttcctgtgtgaaattgt
tatccgctcacaattccacacaacatacgagccggaagcataaagtgtaaagcctggggtgcctaatgagtgagcta
actcacattaattgcgttgcgctcactgcccgctttccagtcgggaaacctgtcgtgccagctgcattaatgaatcg
gccaacgcgcggggagaggcggtttgcgtattgggcgctcttccgcttcctcgctcactgactcgctgcgctcggtc
gttcggctgcggcgagcggtatcagctcactcaaaggcggtaatacggttatccacagaatcaggggataacgcagg
aaagaacatgtgagcaaaaggccagcaaaaggccaggaaccgtaaaaaggccgcgttgctggcgtttttccataggc
tccgcccccctgacgagcatcacaaaaatcgacgctcaagtcagaggtggcgaaacccgacaggactataaagatac
caggcgtttccccctggaagctccctcgtgcgctctcctgttccgaccctgccgcttaccggatacctgtccgcctt
tctcccttcgggaagcgtggcgctttctcaatgctcacgctgtaggtatctcagttcggtgtaggtcgttcgctcca
agctgggctgtgtgcacgaaccccccgttcagcccgaccgctgcgccttatccggtaactatcgtcttgagtccaac
ccggtaagacacgacttatcgccactggcagcagccactggtaacaggattagcagagcgaggtatgtaggcggtgc
tacagagttcttgaagtggtggcctaactacggctacactagaaggacagtatttggtatctgcgctctgctgaagc
cagttaccttcggaaaaagagttggtagctcttgatccggcaaacaaaccaccgctggtagcggtggtttttttgtt
tgcaagcagcagattacgcgcagaaaaaaaggatctcaagaagatcctttgatcttttctacggggtctgacgctca
gtggaacgaaaactcacgttaagggattttggtcatgagattatcaaaaaggatcttcacctagatccttttaaatt
aaaaatgaagttttaaatcaatctaaagtatatatgagtaaacttggtctgacagttaccaatgcttaatcagtgag
gcacctatctcagcgatctgtctatttcgttcatccatagttgcctgactccccgtcgtgtagataactacgatacg
ggagggcttaccatctggccccagtgctgcaatgataccgcgagatccacgctcaccggctccagatttatcagcaa
taaaccagccagccggaagggccgagcgcagaagtggtcctgcaactttatccgcctccatccagtctattaattgt
tgccgggaagctagagtaagtagttcgccagttaatagtttgcgcaacgttgttgccattgctacaggcatcgtggt
gtcacgctcgtcgtttggtatggcttcattcagctccggttcccaacgatcaaggcgagttacatgatcccccatgt
tgtgcaaaaaagcggttagctccttcggtcctccgatcgttgtcagaagtaagttggccgcagtgttatcactcatg
gttatggcagcactgcataattctcttactgtcatgccatccgtaagatgcttttctgtgactggtgagtactcaac
caagtcattctgagaatagtgtatgcggcgaccgagttgctcttgcccggcgtcaatacgggataataccgcgccac
atagcagaactttaaaagtgctcatcattggaaaacgttcttcggggcgaaaactctcaaggatcttaccgctgttg
agatccagttcgatgtaacccactcgtgcacccaactgatcttcagcatcttttactttcaccagcgtttctgggtg
agcaaaaacaggaaggcaaaatgccgcaaaaaagggaataagggcgacacggaaatgttgaatactcatactcttcc
tttttcaatattattgaagcatttatcagggttattgtctcatgagcggatacatatttgaatgtatttagaaaaat
aaacaaataggggttccgcgcacatttccccgaaaagtgccacctgacgtc

*RHD*_Exon1_TALEN_R

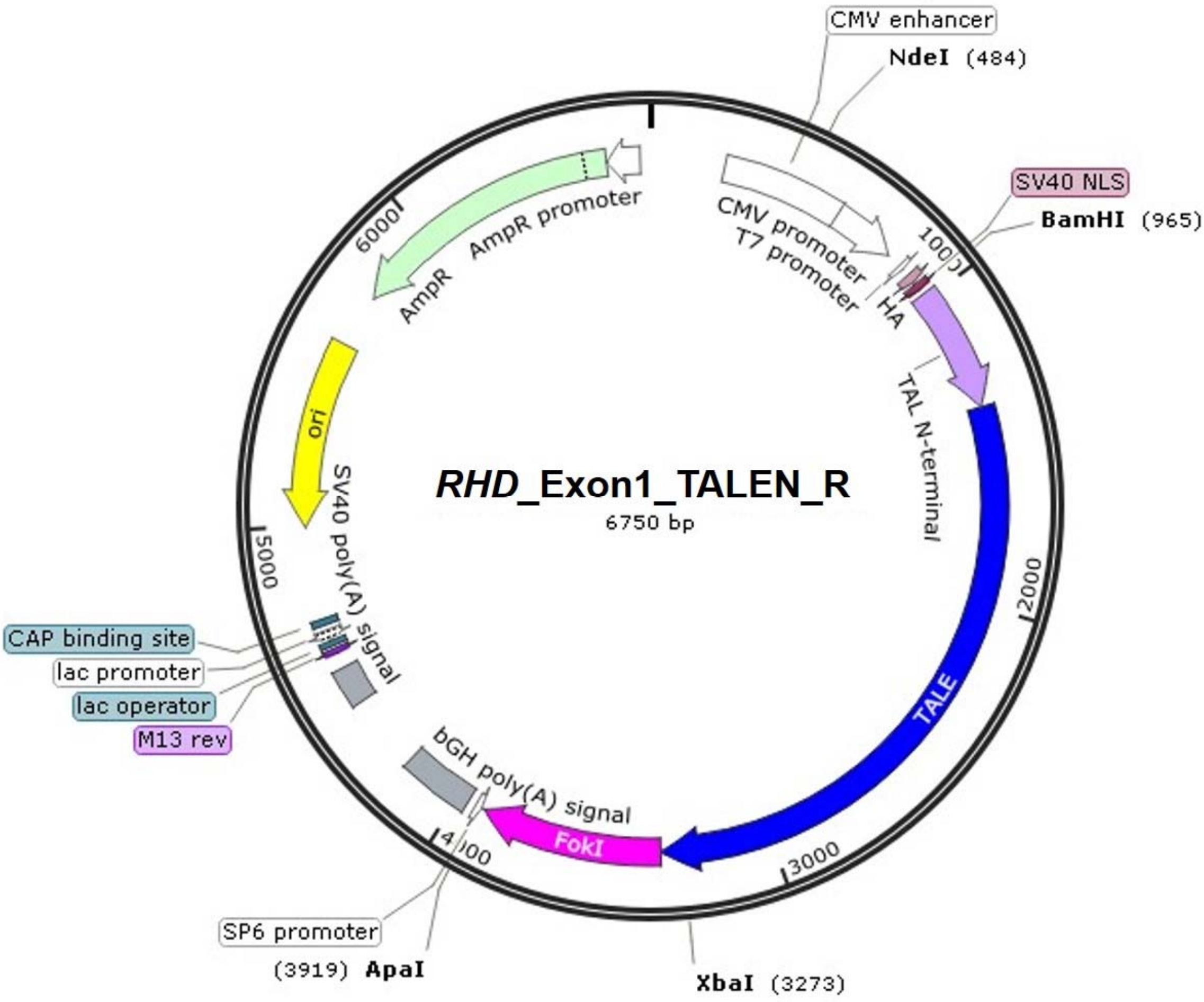

CMV promoter
T7 Promoter
Star codon-HA tag-NLS
TAL N-terminal domain
Target Specific TALE array
Fok I (DAS(Left)/RR(Right) – heterodimer-specific FokI variant)

gacggatcgggagatctcccgatcccctatggtcgactctcagtacaatctgctctgatgccgcatagttaagccag
tatctgctccctgcttgtgtgttggaggtcgctgagtagtgcgcgagcaaaatttaagctacaacaaggcaaggctt
gaccgacaattgcatgaagaatctgcttagggttaggcgttttgcgctgcttcg**cgatgtacgggccagatatacgc**
gttgacattgattattgactagttattaatagtaatcaattacggggtcattagttcatagcccatatatggagttc
cgcgttacataacttacggtaaatggcccgcctggctgaccgcccaacgacccccgcccattgacgtcaataatgac
gtatgttcccatagtaacgccaatagggactttccattgacgtcaatgggtggactatttacggtaaactgcccact
tggcagtacatcaagtgtatcatatgccaagtacgccccctattgacgtcaatgacggtaaatggcccgcctggcat
tatgcccagtacatgaccttatgggactttcctacttggcagtacatctacgtattagtcatcgctattaccatggt

```
gatgcggttttggcagtacatcaatgggcgtggatagcggtttgactcacggggatttccaagtctccaccccattg
acgtcaatgggagtttgttttggcaccaaaatcaacgggactttccaaaatgtcgtaacaactccgccccattgacg
caaatgggcggtaggcgtgtacggtgggaggtctatataagcagagctctctggctaactagagaacccactgctta
ctggcttatcgaaattaatacgactcactatagggagatgcaagcttgccaccatggtgtaccccctacgacgtgccc
gactacgccgaattgcctccaaaaaagaagagaaaggtagggatccgaattcaagatctacgcacgctcggctacag
ccagcagcaacaggagaagatcaaaccgaaggttcgttcgacagtggcgcagcaccacgaggcactggtcggccatg
ggtttacacacgcgcacatcgttgcgctcagccaacacccggcagcgttagggaccgtcgctgtcaagtatcaggac
atgatcgcagcgttgccagaggcgacacacgaagcgatcgttggcgtcggcaaacagtggtccggcgcacgcgctct
ggaggccttgctcacggtggcgggagagttgagaggtccaccgttacagttggacacaggccaacttctcaagattg
caaaacgtggcggcgtgaccgcagtggaggcagtgcatgcatggcgcaatgcactgacgggtgcccccctgaACCTC
ACTCCTGCCCAGGTCGTTGCCATTGCAAGTCATGACGGCGGCAAGCAAGCATTGGAGACAGTGCAGAGACTCTTGCC
CGTCCTGTGTCAAGACCATGGGTTGACTCCTGATCAGGTCGTCGCAATTGCCTCCAATATCGGGGGCAAACAGGCCT
TGGAGACAGTCCAACGCTTGCTCCCAGTTCTTTGCCAAGATCACGGCCTTACACCCGCACAGGTGGTCGCCATCGCT
AGTAATGGTGGGGGCAAACAGGCTCTCGAGACTGTTCAACGGCTTCTCCCAGTTTTGTGTCAAGCCCATGGgttgAC
TCCTGCTCAGGTCGTCGCCATTGCATCTAATATCGGGGGCAAGCAAGCTCTTGAGACTGTCCAAAGACTGCTGCCAG
TGCTGTGCCAAGCCCACGGCCTCACACCAGAGCAGGTGGTGGCCATCGCCAGTAACAATGGCGGTAAACAAGCCTTG
GAAACTGTTCAGAGGCTCCTTCCAGTTCTGTGCCAGGCCCATGGACTTACCCCAGATCAAGTTGTTGCTATTGCTAG
CAACGGGGGCGGAAAACAGGCTCTCGAAACAGTTCAGCGCCTGTTGCCTGTGTTGTGTCAGGATCATGGACTTACac
caGCTCAGGTTGTGGCTATCGCAAGTAACAACGGTGGAAAACAGGCCCTTGAAACTGTCCAACGCCTTCTTCCCGTT
CTCTGCCAGGCTCACGGCTTGACACCCGACCAGGTCGTTGCTATTGCCTCAAACAATGGAGGAAAACAGGCACTCGA
AACCGTCCAGCGGCTGCTCCCCGTCTTGTGCCAGGACCATGGACTGACACCAGAGCAAGTGGTTGCCATCGCCTCCA
ACAACGGTGGTAAGCAAGCACTTGAGACTGTGCAGAGGCTTTTGCCTGTGCTTTGTcaagCCCATGGCCTCACCCCT
GAGCAGGTCGTCGCCATTGCAAGTAATGGAGGAGGGAAACAGGCACTTGAGACAGTCCAAAGGCTCCTTCCAGTTCT
GTGTCAAGCTCATGGACTCACACCAGCCCAGGTGGTCGCAATTGCTTCAAACATTGGGGGAAAGCAGGCTTTGGAGA
CAGTGCAGCGCTTGCTCCCAGTTTTGTGTCAAGACCATGGGTTGACTCCTGCTCAAGTCGTGGCTATCGCCTCAAAT
ATAGGTGGCAAACAGGCCCTCGAAACAGTGCAAAGATTGCTTCCTGTGCTTTGTCAAGCCCATGGCCTgactCCTGA
TCAGGTCGTCGCCATCGCTAGTAACATTGGCGGTAAACAGGCCTTGGAGACAGTCCAGAGGTTGCTCCCCGTGCTCT
GCCAAGCACATGGCCTGACTCCCGCTCAGGTTGTGGCTATTGCCTCCAATATCGGGGGGAAGCAAGCACTTGAAACC
GTCCAGAGGCTGCTCCCTGTGTTGTGTCAAGATCACGGTCTTACCCCAGCTCAAGTGGTCGCTATTGCAAGCAACAT
AGGGGGAAAACAAGCACTGGAAACAGTGCAACGCCTTCTTCCTGTCCTTTGTCAGGCtcatGGACTCACCCCCGATC
AGGTGGTTGCCATCGCATCAAATATCGGTGGGAAACAAGCACTTGAGACTGTCCAACGGTTGTTGCCTGTCCTCTGC
CAGGCTCATGGATTGACTCCCGACCAGGTTGTCGCTATTGCTTCAAACATTGGAGGAAAGCAGGCCTTGGAGACTGT
GCAGCGGCTTCTTCCTGTCCTTTGTCAAGACCATGGCCTGACCCCCGACCAGGTGGTGGCCATCGCCAGCAACGGAG
GCGGCAAGCAGGCCCTGGAAACCGTGCAGAGACTGCTGCCCGTGCTGTGCCAGGCCCAcggtctgactccggaacag
gtggtggcgattgcaagcaatattggcggcaaacaggctctagagagcattgttgcccagctctccagacctgatcc
ggcgctagccgcgttgctagtcaaaagtgaaCtcgaggagaagaaatctgaacttcgtcataaattgaaatatgtgc
ctcatgaatatattgaattaattgaaattgccagaaatcccactcaggatagaattcttgaaatgaaggtaatggaa
tttttatgaaagtttatggatatagaggtgagcatttgggtggatcaaggaaaccggacggagcaatttatactgt
cggatctcctattgattacggtgtgatcgtggatactaaagcttatagcggaggttataatctgccaattggccaag
cacgagaaatgcaacgatatgtcgaagaaaatcaaacacgaaacaaacatatcaaccctaatgaatggtggaaagtc
tatccatcttctgtaacggaatttaagtttttatttgtgagtggtcactttaaaggaaactacaaagctcagcttac
acgattaaatcatatcactaattgtaatggagctgttcttagtgtagaagagcttttaattggtggagaaatgatta
aagccggcacattaaccttagaggaagtgagacggaaatttaataacggcgagataaacttttagggccctattcta
tagtgtcacctaaatgctagagctcgctgatcagcctcgactgtgccttctagttgccagccatctgttgtttgccc
ctcccccgtgccttccttgaccctggaaggtgccactcccactgtcctttcctaataaaatgaggaaattgcatcgc
attgtctgagtaggtgtcattctattctggggggtggggtggggcaggacagcaagggggaggattgggaagacaat
agcaggcatgctggggatgcggtgggctctatggcttctgaggcggaaagaaccagctggggctctagggggtatcc
ccacgctgcagtgagcgggactctggggttcgaaatgaccgaccaagcgacgcccaacctgccatcacgagatttcg
attccaccgccgccttctatgaaaggttgggcttcggaatcgttttccgggacgccggctggatgatcctccagcgc
ggggatctcatgctggagttcttcgcccaccccaacttgtttattgcagcttataatggttacaaataaagcaatag
catcacaaatttcacaaataaagcatttttttcactgcattctagttgtggtttgtccaaactcatcaatgtatctt
atcatgtctgtataccgtcgacctctagctagagcttggcgtaatcatggtcatagctgtttcctgtgtgaaattgt
```

tatccgctcacaattccacacaacatacgagccggaagcataaagtgtaaagcctggggtgcctaatgagtgagcta
actcacattaattgcgttgcgctcactgcccgctttccagtcgggaaacctgtcgtgccagctgcattaatgaatcg
gccaacgcgcggggagaggcggtttgcgtattgggcgctcttccgcttcctcgctcactgactcgctgcgctcggtc
gttcggctgcggcgagcggtatcagctcactcaaaggcggtaatacggttatccacagaatcaggggataacgcagg
aaagaacatgtgagcaaaaggccagcaaaaggccaggaaccgtaaaaaggccgcgttgctggcgtttttccataggc
tccgcccccctgacgagcatcacaaaaatcgacgctcaagtcagaggtggcgaaacccgacaggactataaagatac
caggcgtttccccctggaagctccctcgtgcgctctcctgttccgaccctgccgcttaccggatacctgtccgcctt
tctcccttcgggaagcgtggcgctttctcaatgctcacgctgtaggtatctcagttcggtgtaggtcgttcgctcca
agctgggctgtgtgcacgaaccccccgttcagcccgaccgctgcgccttatccggtaactatcgtcttgagtccaac
ccggtaagacacgacttatcgccactggcagcagccactggtaacaggattagcagagcgaggtatgtaggcggtgc
tacagagttcttgaagtggtggcctaactacggctacactagaaggacagtatttggtatctgcgctctgctgaagc
cagttaccttcggaaaaagagttggtagctcttgatccggcaaacaaaccaccgctggtagcggtggtttttttgtt
tgcaagcagcagattacgcgcagaaaaaaaggatctcaagaagatcctttgatcttttctacggggtctgacgctca
gtggaacgaaaactcacgttaagggattttggtcatgagattatcaaaaaggatcttcacctagatccttttaaatt
aaaaatgaagttttaaatcaatctaaagtatatatgagtaaacttggtctgacagttaccaatgcttaatcagtgag
gcacctatctcagcgatctgtctatttcgttcatccatagttgcctgactccccgtcgtgtagataactacgatacg
ggagggcttaccatctggccccagtgctgcaatgataccgcgagatccacgctcaccggctccagatttatcagcaa
taaaccagccagccggaagggccgagcgcagaagtggtcctgcaactttatccgcctccatccagtctattaattgt
tgccgggaagctagagtaagtagttcgccagttaatagtttgcgcaacgttgttgccattgctacaggcatcgtggt
gtcacgctcgtcgtttggtatggcttcattcagctccggttcccaacgatcaaggcgagttacatgatcccccatgt
tgtgcaaaaaagcggttagctccttcggtcctccgatcgttgtcagaagtaagttggccgcagtgttatcactcatg
gttatggcagcactgcataattctcttactgtcatgccatccgtaagatgcttttctgtgactggtgagtactcaac
caagtcattctgagaatagtgtatgcggcgaccgagttgctcttgcccggcgtcaatacgggataataccgcgccac
atagcagaactttaaaagtgctcatcattggaaaacgttcttcggggcgaaaactctcaaggatcttaccgctgttg
agatccagttcgatgtaacccactcgtgcacccaactgatcttcagcatcttttactttcaccagcgtttctgggtg
agcaaaaacaggaaggcaaaatgccgcaaaaaagggaataagggcgacacggaaatgttgaatactcatactcttcc
tttttcaatattattgaagcatttatcagggttattgtctcatgagcggatacatatttgaatgtatttagaaaaat
aaacaaataggggttccgcgcacatttccccgaaaagtgccacctgacgtc

***RHD*_Exon4_TALEN_L**

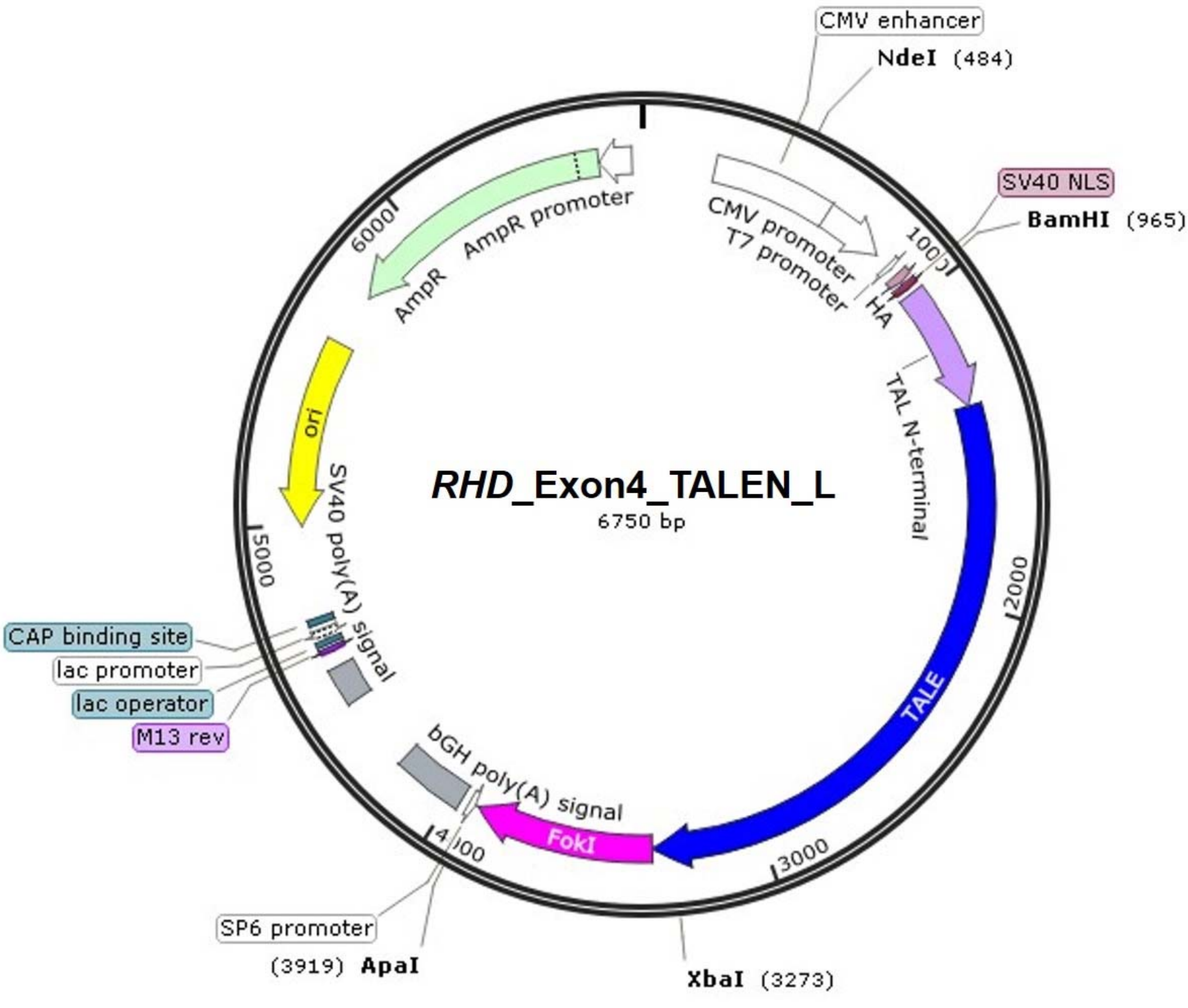

CMV promoter
T7 Promoter
Star codon-HA tag-NLS
TAL N-terminal domain
Target Specific TALE array
Fok I (DAS(Left)/RR(Right) – heterodimer-specific FokI variant)

gacggatcgggagatctcccgatcccctatggtcgactctcagtacaatctgctctgatgccgcatagttaagccag
tatctgctccctgcttgtgtgttggaggtcgctgagtagtgcgcgagcaaaatttaagctacaacaaggcaaggctt
gaccgacaattgcatgaagaatctgcttagggttaggcgttttgcgctgcttcg**cgatgtacgggccagatatacgc**
gttgacattgattattgactagttattaatagtaatcaattacggggtcattagttcatagcccatatatggagttc
cgcgttacataacttacggtaaatggcccgcctggctgaccgcccaacgacccccgcccattgacgtcaataatgac
gtatgttcccatagtaacgccaatagggactttccattgacgtcaatgggtggactatttacggtaaactgcccact
tggcagtacatcaagtgtatcatatgccaagtacgccccctattgacgtcaatgacggtaaatggcccgcctggcat
tatgcccagtacatgacctTatgggactttcctacttggcagtacatctacgtattagtcatcgctattaccatggt
gatgcggttttggcagtacatcaatgggcgtggatagcggtttgactcacggggatttccaagtctccaccccattg
acgtcaatgggagtttgttttggcaccaaaatcaacgggactttccaaaatgtcgtaacaactccgccccattgacg

caaatgggcggtaggcgtgtacggtgggaggtctatataagcagagctctctggctaactagagaacccactgctta
ctggcttatcgaaattaatacgactcactatagggagatgcaagcttgccaccatggtgtacccctacgacgtgccc
gactacgccgaattgcctccaaaaaagaagagaaaggtagggatccgaattcaagatctacgcacgctcggctacag
ccagcagcaacaggagaagatcaaaccgaaggttcgttcgacagtggcgcagcaccacgaggcactggtcggccatg
ggtttacacacgcgcacatcgttgcgctcagccaacacccggcagcgttagggaccgtcgctgtcaagtatcaggac
atgatcgcagcgttgccagaggcgacacacgaagcgatcgttggcgtcggcaaacagtggtccggcgcacgcgctct
ggaggccttgctcacggtggcgggagagttgagaggtccaccgttacagttggacacaggccaacttctcaagattg
caaaacgtggcggcgtgaccgcagtggaggcagtgcatgcatggcgcaatgcactgacgggtgcccccctgaACCTG
ACTCCAGCACAGGTGGTGGCTATTGCCTCCAATAATGGGGGAAAACAGGCTCTCGAAACAGTTCAAAGACTTCTGCC
TGTCTTGTGCCAAGATCATGGACTTACACCCGCACAAGTCGTTGCAATCGCTAGCAATGGCGGTGGGAAGCAAGCCC
TGGAGACAGTTCAAAGGCTCCTTCCAGTCCTGTGTCAGGCACACGGGCTTACCCCTGATCAAGTGGTCGCAATTGCC
TCACATGACGGCGGGAAACAGGCATTGGAAACTGTGCAGCGCCTGCTCCCCGTTCTTTGCCAGGATCATGGgttgAC
ACCTGCACAAGTTGTTGCAATTGCAAGCAACGGCGGCGGAAAACAGGCACTGGAAACTGTGCAAAGACTCCTGCCAG
TTTTGTGCCAGGCTCATGGCCTGACTCCAGACCAGGTCGTTGCTATTGCCAGCAATAACGGAGGTAAGCAAGCATTG
GAGACTGTTCAGCGGCTCCTCCCTGTCCTCTGTCAGGCCCACGGGCTGACTCCTGCACAGGTGGTGGCCATCGCTTC
AAACGGGGGGGGGAAGCAAGCCCTCGAAACTGTTCAACGGTTGTTGCCTGTTTTGTGTCAGGATCATGGGCTTACac
caGAACAAGTCGTGGCAATCGCTTCAAATAATGGCGGAAAGCAAGCTCTCGAAACCGTCCAGAGACTCTTGCCTGTT
CTTTGCCAGGCCCACGGGTTGACCCCTGCTCAGGTGGTCGCTATTGCCTCTAACAACGGTGGTAAACAGGCCCTCGA
AACCGTCCAAAGGCTTCTGCCCGTGCTCTGTCAAGACCACGGGCTCACACCCGACCAAGTCGTTGCAATCGCTAGTC
ACGACGGAGGGAAGCAGGCATTGGAAACAGTGCAGCGGCTGCTTCCTGTTTTGTGCcaagCACATGGCCTCACACCT
GCCCAAGTCGTCGCAATCGCCTCCCACGACGGCGGCAAGCAGGCATTGGAAACCGTGCAAAGACTTTTGCCAGTGCT
GTGCCAAGCACACGGCCTCACCCCAGCACAAGTGGTGGCTATCGCTAGCAACGGTGGGGGCAAGCAGGCCCTTGAGA
CAGTTCAGAGGCTCCTTCCTGTTCTCTGTCAGGATCATGGTTTGACACCAGATCAGGTGGTCGCTATTGCTTCCAAC
AATGGCGGGAAACAAGCTTTGGAGACTGTGCAACGGTTGCTGCCTGTTCTCTGCCAGGACCACGGGCTgactCCCGA
ACAAGTCGTCGCTATTGCAAGCAACAACGGGGGGAAACAGGCCCTGGAGACTGTCCAGCGGCTGTTGCCTGTTCTCT
GTCAGGCACATGGCCTCACCCCTGACCAAGTGGTGGCTATCGCATCTAACGGCGGTGGCAAGCAGGCCTTGGAAACA
GTGCAAAGGCTTCTGCCAGTCTTGTGCCAGGATCATGGGCTCACACCAGCTCAGGTTGTTGCAATCGCCAGTAATAA
CGGAGGTAAGCAAGCTCTGGAAACCGTGCAGAGGTTGCTGCCTGTTCTGTGTCAAGCtcatGGGCTGACTCCTGACC
AGGTCGTTGCCATTGCTAGTCATGACGGGGGTAAACAGGCTCTCGAAACTGTGCAACGCTTGTTGCCAGTTTTGTGC
CAAGCTCATGGCTTGACACCTGCACAAGTGGTTGCAATCGCTTCACACGACGGAGGAAAGCAAGCCCTCGAAACTGT
GCAGCGGCTCCTCCCCGTGCTGTGTCAAGATCACGGCCTTACACCCGCTCAAGTCGTCGCAATTGCCAGCAATGGAG
GCGGTAAGCAGGCCTTGGAGACTGTTCAGAGGTTGTTGCCCGTGCTTTGCCAAGACCAcggtctgactccggaacag
gtggtggcgattgcaagcaataacggcggcaaacaggctctagagagcattgttgcccagctctccagacctgatcc
ggcgctagccgcgttgctagtcaaaagtgaaCtcgaggagaagaaatctgaacttcgtcataaattgaaatatgtgc
ctcatgaatatattgaattaattgaaattgccagaaatcccactcaggatagaattcttgaaatgaaggtaatggaa
ttttttatgaaagtttatggatatagaggtgagcatttgggtggatcaaggaaaccggacggagcaatttatactgt
cggatctcctattgattacggtgtgatcgtggatactaaagcttatagcggaggttataatctgccaattggccaag
cagatgccatgcaaagctatgtcgaagaaaatcaaacacgaaacaaacatatcaaccctaatgaatggtggaaagtc
tatccatcttctgtaacggaatttaagtttttatttgtgagtggtcactttaaaggaaactacaaagctcagcttac
acgattaaatcatatcactaattgtaatggagctgttcttagtgtagaagagcttttaattggtggagaaatgatta
aagccggcacattaaccttagaggaagtgagacggaaatttaataacggcgagataaacttttagggccctattcta
tagtgtcacctaaatgctagagctcgctgatcagcctcgactgtgccttctagttgccagccatctgttgtttgccc
ctcccccgtgccttccttgaccctggaaggtgccactcccactgtcctttcctaataaaatgaggaaattgcatcgc
attgtctgagtaggtgtcattctattctggggggtggggtggggcaggacagcaagggggaggattgggaagacaat
agcaggcatgctggggatgcggtgggctctatggcttctgaggcggaaagaaccagctggggctctagggggtatcc
ccacgctgcagtgagcgggactctggggttcgaaatgaccgaccaagcgacgcccaacctgccatcacgagatttcg
attccaccgccgccttctatgaaaggttgggcttcggaatcgttttccgggacgccggctggatgatcctccagcgc
ggggatctcatgctggagttcttcgcccaccccaacttgtttattgcagcttataatggttacaaataaagcaatag
catcacaaatttcacaaataaagcatttttttcactgcattctagttgtggtttgtccaaactcatcaatgtatctt
atcatgtctgtataccgtcgacctctagctagagcttggcgtaatcatggtcatagctgtttcctgtgtgaaattgt
tatccgctcacaattccacacaacatacgagccggaagcataaagtgtaaagcctggggtgcctaatgagtgagcta
actcacattaattgcgttgcgctcactgcccgctttccagtcgggaaacctgtcgtgccagctgcattaatgaatcg

gccaacgcgcggggagaggcggtttgcgtattgggcgctcttccgcttcctcgctcactgactcgctgcgctcggtc
gttcggctgcggcgagcggtatcagctcactcaaaggcggtaatacggttatccacagaatcaggggataacgcagg
aaagaacatgtgagcaaaaggccagcaaaaggccaggaaccgtaaaaaggccgcgttgctggcgtttttccataggc
tccgcccccctgacgagcatcacaaaaatcgacgctcaagtcagaggtggcgaaacccgacaggactataaagatac
caggcgtttccccctggaagctccctcgtgcgctctcctgttccgaccctgccgcttaccggatacctgtccgcctt
tctcccttcgggaagcgtggcgctttctcaatgctcacgctgtaggtatctcagttcggtgtaggtcgttcgctcca
agctgggctgtgtgcacgaaccccccgttcagcccgaccgctgcgccttatccggtaactatcgtcttgagtccaac
ccggtaagacacgacttatcgccactggcagcagccactggtaacaggattagcagagcgaggtatgtaggcggtgc
tacagagttcttgaagtggtggcctaactacggctacactagaaggacagtatttggtatctgcgctctgctgaagc
cagttaccttcggaaaaagagttggtagctcttgatccggcaaacaaaccaccgctggtagcggtggtttttttgtt
tgcaagcagcagattacgcgcagaaaaaaaggatctcaagaagatcctttgatcttttctacggggtctgacgctca
gtggaacgaaaactcacgttaagggattttggtcatgagattatcaaaaaggatcttcacctagatccttttaaatt
aaaaatgaagttttaaatcaatctaaagtatatatgagtaaacttggtctgacagttaccaatgcttaatcagtgag
gcacctatctcagcgatctgtctatttcgttcatccatagttgcctgactccccgtcgtgtagataactacgatacg
ggagggcttaccatctggccccagtgctgcaatgataccgcgagatccacgctcaccggctccagatttatcagcaa
taaaccagccagccggaagggccgagcgcagaagtggtcctgcaactttatccgcctccatccagtctattaattgt
tgccgggaagctagagtaagtagttcgccagttaatagtttgcgcaacgttgttgccattgctacaggcatcgtggt
gtcacgctcgtcgtttggtatggcttcattcagctccggttcccaacgatcaaggcgagttacatgatcccccatgt
tgtgcaaaaaagcggttagctccttcggtcctccgatcgttgtcagaagtaagttggccgcagtgttatcactcatg
gttatggcagcactgcataattctcttactgtcatgccatccgtaagatgcttttctgtgactggtgagtactcaac
caagtcattctgagaatagtgtatgcggcgaccgagttgctcttgcccggcgtcaatacgggataataccgcgccac
atagcagaactttaaaagtgctcatcattggaaaacgttcttcggggcgaaaactctcaaggatcttaccgctgttg
agatccagttcgatgtaacccactcgtgcacccaactgatcttcagcatcttttactttcaccagcgtttctgggtg
agcaaaaacaggaaggcaaaatgccgcaaaaaagggaataagggcgacacggaaatgttgaatactcatactcttcc
tttttcaatattattgaagcatttatcagggttattgtctcatgagcggatacatatttgaatgtatttagaaaaat
aaacaaataggggttccgcgcacatttccccgaaaagtgccacctgacgtc

***RHD*_Exon4_TALEN_R**

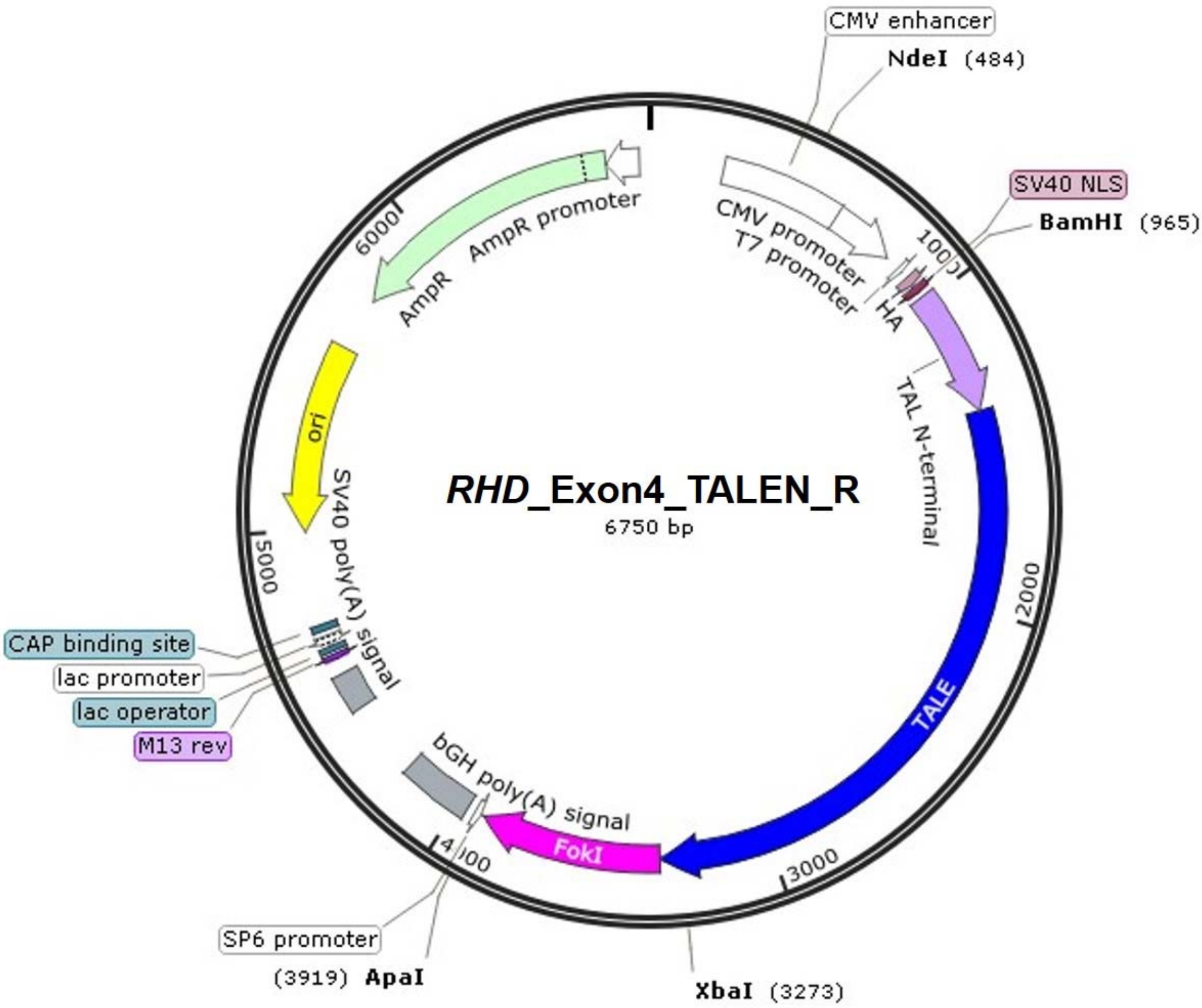

CMV promoter
T7 Promoter
Star codon-HA tag-NLS
TAL N-terminal domain
Target Specific TALE array
Fok I (DAS(Left)/RR(Right) – heterodimer-specific FokI variant)

gacggatcgggagatctcccgatcccctatggtcgactctcagtacaatctgctctgatgccgcatagttaagccag
tatctgctccctgcttgtgtgttggaggtcgctgagtagtgcgcgagcaaaatttaagctacaacaaggcaaggctt
gaccgacaattgcatgaagaatctgcttagggttaggcgttttgcgctgcttcg**cgatgtacgggccagatatacgc**
gttgacattgattattgactagttattaatagtaatcaattacggggtcattagttcatagcccatatatggagttc
cgcgttacataacttacggtaaatggcccgcctggctgaccgcccaacgacccccgcccattgacgtcaataatgac
gtatgttcccatagtaacgccaatagggactttccattgacgtcaatgggtggactatttacggtaaactgcccact
tggcagtacatcaagtgtatcatatgccaagtacgccccctattgacgtcaatgacggtaaatggcccgcctggcat
tatgcccagtacatgaccttatgggactttcctacttggcagtacatctacgtattagtcatcgctattaccatggt

gatgcggttttggcagtacatcaatgggcgtggatagcggtttgactcacggggatttccaagtctccaccccattg
acgtcaatgggagtttgttttggcaccaaaatcaacgggactttccaaaatgtcgtaacaactccgccccattgacg
caaatgggcggtaggcgtgtacggtgggaggtctatataagcagagctctctggctaactagagaacccactgctta
ctggcttatcgaaattaatacgactcactatagggagatgcaagcttgccaccatggtgtacccctacgacgtgccc
gactacgccgaattgcctccaaaaaagaagagaaaggtagggatccgaattcaagatctacgcacgctcggctacag
ccagcagcaacaggagaagatcaaaccgaaggttcgttcgacagtggcgcagcaccacgaggcactggtcggccatg
ggtttacacacgcgcacatcgttgcgctcagccaacacccggcagcgttagggaccgtcgctgtcaagtatcaggac
atgatcgcagcgttgccagaggcgacacacgaagcgatcgttggcgtcggcaaacagtggtccggcgcacgcgctct
ggaggccttgctcacggtggcgggagagttgagaggtccaccgttacagttggacacaggccaacttctcaagattg
caaaacgtggcggcgtgaccgcagtggaggcagtgcatgcatggcgcaatgcactgacgggtgccccctgaACCTC
ACACCTGACCAGGTCGTGGCCATTGCCAGCAATGGAGGAGGGAAGCAAGCTCTTGAGACTGTGCAGCGCTTGCTTCC
CGTCCTCTGCCAAGCACATGGGCTCACACCCGATCAAGTTGTTGCTATTGCAAGCAATATCGGAGGCAAGCAGGCCT
TGGAAACTGTCCAAAGATTGTTGCCAGTCTTGTGCCAGGATCACGGACTCACCCCAGACCAAGTGGTTGCAATTGCT
TCAAATGGCGGGGGCAAACAGGCCCTGGAAACCGTTCAGCGGCTTCTTCCTGTCCTTTGCCAGGCCCATGGgttgAC
TCCTGACCAGGTCGTTGCCATTGCTAGTCATGACGGGGGTAAACAGGCTCTCGAAACTGTGCAACGCTTGTTGCCAG
TTTTGTGCCAAGCTCATGGCTTGACACCTGCACAAGTGGTTGCAATCGCTTCACACGACGGAGGAAAGCAAGCCCTC
GAAACTGTGCAGCGGCTCCTCCCCGTGCTGTGTCAAGATCACGGCCTTACACCCGCTCAAGTCGTCGCAATTGCCAG
CAATGGAGGCGGTAAGCAGGCCTTGGAGACTGTTCAGAGGTTGTTGCCCGTGCTTTGCCAAGACCACGGGCTGACac
caGCTCAGGTTGTTGCTATTGCATCTCACGACGGCGGAAAGCAAGCTCTCGAGACAGTCCAGAGGTTGCTTCCTGTC
CTTTGCCAGGACCACGGTCTTACACCTGACCAAGTTGTCGCCATCGCCTCCCATGATGGTGGGAAACAGGCCCTGGA
AACTGTGCAGCGCCTTCTGCCTGTCCTCTGTCAGGCCCACGGATTGACACCCGAGCAGGTGGTCGCCATTGCTTCTA
ACAACGGGGGCAAGCAGGCACTTGAGACTGTCCAGAGGCTCCTCCCCGTTCTTTGTcaagCCCATGGCTTGACACCT
GACCAAGTCGTTGCAATCGCTTCAAACGGTGGAGGTAAACAAGCTTTGGAAACCGTCCAACGCCTTCTTCCAGTTCT
TTGTCAGGATCATGGTCTTACACCTGAGCAGGTGGTTGCAATTGCCAGCAATGGTGGAGGCAAACAAGCTCTGGAGA
CAGTGCAGAGACTTTTGCCTGTCCTTTGCCAGGCCCACGGATTGACCCCAGACCAGGTTGTCGCTATTGCATCACAT
GACGGTGGCAAGCAAGCTCTCGAAACTGTCCAGAGATTGCTCCCTGTCTTGTGTCAAGCACATGGTTTgactCCTGA
CCAGGTCGTTGCCATTGCTAGTCATGACGGGGGTAAACAGGCTCTCGAAACTGTGCAACGCTTGTTGCCAGTTTTGT
GCCAAGCTCATGGCTTGACACCTGCACAAGTGGTTGCAATCGCTTCACACGACGGAGGAAAGCAAGCCCTCGAAACT
GTGCAGCGGCTCCTCCCCGTGCTGTGTCAAGATCACGGCCTTACACCCGCTCAAGTCGTCGCAATTGCCAGCAATGG
AGGCGGTAAGCAGGCCTTGGAGACTGTTCAGAGGTTGTTGCCCGTGCTTTGCCAAGAtcatGGCCTGACTCCCGAAC
AGGTGGTCGCAATTGCATCACACGACGGAGGGAAGCAGGCCTTGGAAACAGTGCAAAGACTCTTGCCTGTTCTCTGC
CAAGCTCACGGGCTCACTCCTGCTCAAGTTGTGGCCATCGCTAGCAACAATGGTGGTAAACAGGCATTGGAAACAGT
CCAGAGGCTTCTCCCAGTTCTTTGCCAGGATCATGGGTTGACCCCCGACCAAGTGGTTGCCATTGCCTCCAATAACG
GGGGAAAACAGGCTCTGGAAACCGTTCAGCGCCTTCTCCCTGTGTTGTGTCAAGATCAcggtctgactccggaacag
gtggtggcgattgcaagcaataacggcggcaaacaggctctagagagcattgttgcccagctctccagacctgatcc
ggcgctagccgcgttgctagtcaaaagtgaaCtcgaggagaagaaatctgaacttcgtcataaattgaaatatgtgc
ctcatgaatatattgaattaattgaaattgccagaaatcccactcaggatagaattcttgaaatgaaggtaatggaa
tttttatgaaagtttatggatatagaggtgagcatttgggtggatcaaggaaaccggacggagcaatttatactgt
cggatctcctattgattacggtgtgatcgtggatactaaagcttatagcggaggttataatctgccaattggccaag
cacgagaaatgcaacgatatgtcgaagaaaatcaaacacgaaacaaacatatcaaccctaatgaatggtggaaagtc
tatccatcttctgtaacggaatttaagtttttatttgtgagtggtcactttaaaggaaactacaaagctcagcttac
acgattaaatcatatcactaattgtaatggagctgttcttagtgtagaagagcttttaattggtggagaaatgatta
aagccggcacattaaccttagaggaagtgagacggaaatttaataacggcgagataaacttttagggccctattcta
tagtgtcacctaaatgctagagctcgctgatcagcctcgactgtgccttctagttgccagccatctgttgtttgccc
ctcccccgtgccttccttgaccctggaaggtgccactcccactgtcctttcctaataaaatgaggaaattgcatcgc
attgtctgagtaggtgtcattctattctggggggtggggtggggcaggacagcaagggggaggattgggaagacaat
agcaggcatgctggggatgcggtgggctctatggcttctgaggcggaaagaaccagctggggctctagggggtatcc
ccacgctgcagtgagcgggactctggggttcgaaatgaccgaccaagcgacgcccaacctgccatcacgagatttcg
attccaccgccgccttctatgaaaggttgggcttcggaatcgttttccgggacgccggctggatgatcctccagcgc
ggggatctcatgctggagttcttcgcccaccccaacttgtttattgcagcttataatggttacaaataaagcaatag
catcacaaatttcacaaataaagcatttttttcactgcattctagttgtggtttgtccaaactcatcaatgtatctt
atcatgtctgtataccgtcgacctctagctagagcttggcgtaatcatggtcatagctgtttcctgtgtgaaattgt

tatccgctcacaattccacacaacatacgagccggaagcataaagtgtaaagcctggggtgcctaatgagtgagcta
actcacattaattgcgttgcgctcactgcccgctttccagtcgggaaacctgtcgtgccagctgcattaatgaatcg
gccaacgcgcggggagaggcggtttgcgtattgggcgctcttccgcttcctcgctcactgactcgctgcgctcggtc
gttcggctgcggcgagcggtatcagctcactcaaaggcggtaatacggttatccacagaatcaggggataacgcagg
aaagaacatgtgagcaaaaggccagcaaaaggccaggaaccgtaaaaaggccgcgttgctggcgtttttccataggc
tccgcccccctgacgagcatcacaaaaatcgacgctcaagtcagaggtggcgaaacccgacaggactataaagatac
caggcgtttccccctggaagctccctcgtgcgctctcctgttccgaccctgccgcttaccggatacctgtccgcctt
tctcccttcgggaagcgtggcgctttctcaatgctcacgctgtaggtatctcagttcggtgtaggtcgttcgctcca
agctgggctgtgtgcacgaaccccccgttcagcccgaccgctgcgccttatccggtaactatcgtcttgagtccaac
ccggtaagacacgacttatcgccactggcagcagccactggtaacaggattagcagagcgaggtatgtaggcggtgc
tacagagttcttgaagtggtggcctaactacggctacactagaaggacagtatttggtatctgcgctctgctgaagc
cagttaccttcggaaaaagagttggtagctcttgatccggcaaacaaaccaccgctggtagcggtggtttttttgtt
tgcaagcagcagattacgcgcagaaaaaaaggatctcaagaagatcctttgatcttttctacggggtctgacgctca
gtggaacgaaaactcacgttaagggattttggtcatgagattatcaaaaaggatcttcacctagatccttttaaatt
aaaaatgaagttttaaatcaatctaaagtatatatgagtaaacttggtctgacagttaccaatgcttaatcagtgag
gcacctatctcagcgatctgtctatttcgttcatccatagttgcctgactccccgtcgtgtagataactacgatacg
ggagggcttaccatctggccccagtgctgcaatgataccgcgagatccacgctcaccggctccagatttatcagcaa
taaaccagccagccggaagggccgagcgcagaagtggtcctgcaactttatccgcctccatccagtctattaattgt
tgccgggaagctagagtaagtagttcgccagttaatagtttgcgcaacgttgttgccattgctacaggcatcgtggt
gtcacgctcgtcgtttggtatggcttcattcagctccggttcccaacgatcaaggcgagttacatgatcccccatgt
tgtgcaaaaaagcggttagctccttcggtcctccgatcgttgtcagaagtaagttggccgcagtgttatcactcatg
gttatggcagcactgcataattctcttactgtcatgccatccgtaagatgcttttctgtgactggtgagtactcaac
caagtcattctgagaatagtgtatgcggcgaccgagttgctcttgcccggcgtcaatacgggataataccgcgccac
atagcagaactttaaaagtgctcatcattggaaaacgttcttcggggcgaaaactctcaaggatcttaccgctgttg
agatccagttcgatgtaacccactcgtgcacccaactgatcttcagcatcttttactttcaccagcgtttctgggtg
agcaaaaacaggaaggcaaaatgccgcaaaaaagggaataagggcgacacggaaatgttgaatactcatactcttcc
tttttcaatattattgaagcatttatcagggttattgtctcatgagcggatacatatttgaatgtatttagaaaaat
aaacaaataggggttccgcgcacatttccccgaaaagtgccacctgacgtc

Supplementary Reference

47. Flegel, W.A. et al. In-frame triplet deletions in RHD alter the D antigen phenotype. *Transfusion* **46**, 2156-2161 (2006).

Supplementary Dataset 1. Potential off-target sites for RHD_E1_TALENs and RHD_E4_TALENs. Sites indicated with red fonts (ranking 1) represent the on-target sites.
